# Cardiovascular and Renal Outcomes of Renin–Angiotensin System Blockade in Adult Patients with Diabetes Mellitus: A Systematic Review with Network Meta-Analyses

**DOI:** 10.1371/journal.pmed.1001971

**Published:** 2016-03-08

**Authors:** Ferrán Catalá-López, Diego Macías Saint-Gerons, Diana González-Bermejo, Giuseppe M. Rosano, Barry R. Davis, Manuel Ridao, Abel Zaragoza, Dolores Montero-Corominas, Aurelio Tobías, César de la Fuente-Honrubia, Rafael Tabarés-Seisdedos, Brian Hutton

**Affiliations:** 1 Department of Medicine, University of Valencia/INCLIVA Health Research Institute, Valencia, Spain; 2 Division of Pharmacoepidemiology and Pharmacovigilance, Spanish Agency of Medicines and Medical Devices (AEMPS), Madrid, Spain; 3 Clinical Epidemiology Program, Ottawa Hospital Research Institute, Ottawa, Ontario, Canada; 4 Centro de Investigación Biomédica en Red de Salud Mental (CIBERSAM), Instituto de Salud Carlos III, Madrid, Spain; 5 Centre for Clinical and Basic Research, Department of Medical Sciences, Istituto di Ricovero e Cura a Carattere Scientifico San Raffaele Pisana, Rome, Italy; 6 The University of Texas School of Public Health, Houston, Texas, United States of America; 7 Instituto Aragonés de Ciencias de la Salud, Red de Investigación en Servicios de Salud en Enfermedades Crónicas (REDISSEC), Zaragoza, Spain; 8 Fundación para el Fomento de la Investigación Sanitaria y Biomédica de la Comunitat Valenciana (FISABIO–Salud Pública), Valencia, Spain; 9 Spanish Council for Scientific Research (CSIC), Barcelona, Spain; 10 Area of Budgetary Stability, Ministry of Finance and Public Administrations, Madrid, Spain; 11 School of Epidemiology, Public Health and Preventive Medicine, Faculty of Medicine, University of Ottawa, Ottawa, Ontario, Canada; Royal Derby Hospital, UNITED KINGDOM

## Abstract

**Background:**

Medications aimed at inhibiting the renin–angiotensin system (RAS) have been used extensively for preventing cardiovascular and renal complications in patients with diabetes, but data that compare their clinical effectiveness are limited. We aimed to compare the effects of classes of RAS blockers on cardiovascular and renal outcomes in adults with diabetes.

**Methods and Findings:**

Eligible trials were identified by electronic searches in PubMed/MEDLINE and the Cochrane Database of Systematic Reviews (1 January 2004 to 17 July 2014). Interventions of interest were angiotensin-converting enzyme (ACE) inhibitors, angiotensin receptor blockers (ARBs), and direct renin (DR) inhibitors. The primary endpoints were cardiovascular mortality, myocardial infarction, and stroke—singly and as a composite endpoint, major cardiovascular outcome—and end-stage renal disease [ESRD], doubling of serum creatinine, and all-cause mortality—singly and as a composite endpoint, progression of renal disease. Secondary endpoints were angina pectoris and hospitalization for heart failure. In all, 71 trials (103,120 participants), with a total of 14 different regimens, were pooled using network meta-analyses. When compared with ACE inhibitor, no other RAS blocker used in monotherapy and/or combination was associated with a significant reduction in major cardiovascular outcomes: ARB (odds ratio [OR] 1.02; 95% credible interval [CrI] 0.90–1.18), ACE inhibitor plus ARB (0.97; 95% CrI 0.79–1.19), DR inhibitor plus ACE inhibitor (1.32; 95% CrI 0.96–1.81), and DR inhibitor plus ARB (1.00; 95% CrI 0.73–1.38). For the risk of progression of renal disease, no significant differences were detected between ACE inhibitor and each of the remaining therapies: ARB (OR 1.10; 95% CrI 0.90–1.40), ACE inhibitor plus ARB (0.97; 95% CrI 0.72–1.29), DR inhibitor plus ACE inhibitor (0.99; 95% CrI 0.65–1.57), and DR inhibitor plus ARB (1.18; 95% CrI 0.78–1.84). No significant differences were showed between ACE inhibitors and ARBs with respect to all-cause mortality, cardiovascular mortality, myocardial infarction, stroke, angina pectoris, hospitalization for heart failure, ESRD, or doubling serum creatinine. Findings were limited by the clinical and methodological heterogeneity of the included studies. Potential inconsistency was identified in network meta-analyses of stroke and angina pectoris, limiting the conclusiveness of findings for these single endpoints.

**Conclusions:**

In adults with diabetes, comparisons of different RAS blockers showed similar effects of ACE inhibitors and ARBs on major cardiovascular and renal outcomes. Compared with monotherapies, the combination of an ACE inhibitor and an ARB failed to provide significant benefits on major outcomes. Clinicians should discuss the balance between benefits, costs, and potential harms with individual diabetes patients before starting treatment.

**Review registration:**

PROSPERO CRD42014014404

## Abstract 2

In a systematic review with network meta-analyses, Ferrán Catalá-López and colleagues synthesize published and unpublished data from randomized controlled trials of renin-angiotensin system inhibitors.

## Editors' Summary

**Background:**

Chronic high blood pressure can damage blood vessels, heart, and kidneys, and cause cardiovascular and kidney (or renal) disease. Diabetes increases the risk for high blood pressure. An estimated two-thirds of adults with diabetes have high blood pressure or take blood-pressure-reducing drugs (also called antihypertensives). Because diabetes itself increases the risk for heart and kidney diseases, controlling blood pressure is important. Several of the drugs commonly prescribed to patients with diabetes target the renin–angiotensin system (RAS), a hormone system that regulates blood pressure and fluid balance. When renal blood flow is low, kidney cells produce renin and secrete it into the blood stream. Renin in the blood generates a protein called angiotensin I. The angiotensin-converting enzyme (ACE) in the lungs subsequently converts angiotensin I to angiotensin II, and angiotensin II docks with its partner, the angiotensin receptor. This then leads to a signaling cascade that causes blood vessels to constrict and the kidneys to secrete water and salt into the blood, resulting in increased blood pressure.

Drugs that interrupt different steps in the cascade can lower blood pressure and thereby prevent cardiovascular and renal disease. In addition to their blood-pressure-lowering effects, some RAS blockers have also been shown to protect heart and kidney function through other mechanisms. RAS blockers fall into three main classes: ACE inhibitors block the conversion of angiotensin I into angiotensin II, angiotensin receptor blockers (ARBs) prevent angiotensin II from activating the angiotensin receptor, and direct renin inhibitors inhibit the production of angiotensin I.

**Why Was This Study Done?:**

As many adults with diabetes are prescribed RAS blockers and likely need to take them or related drugs for the rest of their lives, the question of which drug (or drug combination) has the best results and the least side effects is important. Data from many randomized controlled trials (RCTs, which are the most rigorous clinical tests) have generated results that address this question. Their combined analysis, however, is complicated because the trials often don’t compare all treatments directly, don’t use the exact same treatments in patients with the exact same conditions, or don’t measure the exact same outcomes. Nonetheless, scientists have developed methods to analyze such complex data, including a method called network meta-analysis, which helps to integrate and synthesize diverse results to determine the relative merits of multiple treatments. Here the researchers apply network meta-analysis to all available data from RCTs in which adults with diabetes were given RAS blockers, alone or in combination with other high blood pressure treatments, and that measured cardiovascular or renal outcomes.

**What Did the Researchers Do and Find?:**

The researchers started with a systematic search of the medical literature for RCTs that tested RAS blockers alone or in combination in adults with diabetes. They also contacted the sponsors of many studies to find out whether they had additional data that were not included in the published reports. They included all trials that had at least 100 participants, followed the participants for at least 12 months, and reported cardiovascular or renal outcomes. In all, 71 trials that tested a total of 14 different treatment regimens in 103,120 participants met these criteria. With network meta-analysis, the researchers were able to summarize the results of all these trials in a meaningful way. They did this by integrating direct comparisons of RAS blockers within the same trial (where those were available) with indirect comparisons across all trials. The primary endpoints in their analysis were major cardiovascular outcome (a composite of cardiovascular mortality, nonfatal heart attack, and nonfatal stroke) and progression of renal disease (a composite of end-stage renal disease, doubling of serum creatinine [a marker of reduced kidney function], and all-cause mortality). They also analyzed the individual cardiovascular or renal outcomes separately, but these results carried more uncertainty because fewer patients had the individual, compared with the combined, outcomes.

The researchers found no significant differences in the risk of major cardiovascular outcome between ACE inhibitors and either ARBs or a combination of an ACE inhibitor plus an ARB. Similarly, for the risk of progression of renal disease, no significant differences were detected between ACE inhibitors and any of the remaining therapies, such as ARBs or a combination of an ACE inhibitor plus an ARB. They also found that no RAS blocker strategy was superior to ACE inhibitors with respect to all-cause mortality, cardiovascular mortality, heart attacks, strokes, end-stage renal disease, or doubling of serum creatinine. Overall, any single ACE inhibitor or ARB was equally effective as any other, and just as effective as any drug combination.

**What Do These Findings Mean?:**

The findings reinforce North American and European guidelines that recommend ACE inhibitors and ARBs for antihypertensive therapy in patients with diabetes. The fact that combinations of two drugs had outcomes no better than those of a single ACE inhibitor or ARB—together with results from other studies that reported increased adverse effects in patients who took drug combinations—cautions against the use of combination therapy. The findings also contradict earlier reports suggesting that ARBs may increase the risk of cardiovascular outcomes. There were not enough data comparing direct renin inhibitors (a drug class that was developed more recently and therefore has been studied less) with ACE inhibitors or ARBs to allow strong conclusions; additional research might therefore be warranted.

The analyses here did not consider the costs of any particular drug, or any side effects that were not relevant to the outcomes measured. These factors, together with the results here and a patient’s specific situation, should be taken into account when doctors and their patients discuss and decide appropriate treatments.

**Additional Information:**

This list of resources contains links that can be accessed when viewing the PDF on a device or via the online version of the article at http://dx.doi.org/10.1371/journal.pmed.1001971.

- The MedlinePlus has links to information on diabetes
- The American Diabetes Association provides information on kidney disease, heart disease, and high blood pressure
- The European Society of Cardiology has guidelines on diabetes, heart disease, and high blood pressure
- The UK National Institute for Health and Care Excellence has guidelines on renin–angiotensin system drugs

## Introduction

Diabetes mellitus has become one of the most challenging public health problems worldwide, affecting approximately 410 million people [1] and accounting for 1.3 million deaths in 2013, twice as many as in 1990 [2]. Complications of diabetes mellitus, especially cardiovascular and renal sequelae, cause substantial premature death and disability [1–4].

Medications aimed at inhibiting the renin–angiotensin system (RAS) have been used extensively for preventing cardiovascular and renal outcomes in patients with diabetes. Blockade of the RAS is a key therapeutic target because RAS controls circulatory volume and electrolyte balance and is an important regulator of hemodynamic stability. Currently, three classes of drugs that interact with the RAS are used to inhibit the effects of angiotensin II: angiotensin-converting enzyme (ACE) inhibitors, angiotensin receptor blockers (ARBs), and direct renin (DR) inhibitors. ACE inhibitors block the conversion of angiotensin I into angiotensin II, ARBs selectively inhibit angiotensin II from activating the angiotensin-specific receptor AT1, and DR inhibitors block the conversion of angiotensinogen into angiotensin I. Although all RAS blockers are intended to inhibit the effects of angiotensin II, there are differences that may distinguish them [5].

Most evidence-based guidelines for the management of hypertension and diabetes have generally recommended the use of ACE inhibitors and ARBs in preference to other antihypertensive agents [6–9]. In these guidelines, any particular RAS blocker (ACE inhibitor or ARB) is preferentially recommended as the treatment of choice. However, current guidelines are based on only a small number of randomized trials comparing the effects of RAS blockade specifically in patients with diabetes. Cardiovascular and renal outcomes with RAS blockers for adults with diabetes have been evaluated in large multicenter randomized controlled trials [10–22] and meta-analyses [23–28]. The task of establishing the comparative effectiveness of RAS blockers has been limited by the very complex array of trials that compare treatments. Results of recent meta-analyses have highlighted potential differences in treatment effects between ACE inhibitors and ARBs [23,24]. Traditionally, meta-analyses of RAS blockers have been limited by not including all the valuable information on the most common serious cardiovascular and renal outcomes [23–28], not exploring effects in the subgroup of patients with diabetes [29–37], and, importantly, omitting large trials with direct comparisons of RAS blockers and competing agents in clinically important subgroups [22,38–41]. Determining whether RAS blockers may be different in terms of their relative benefits and safety is a topic of great interest to patients, clinicians, scientists, guideline developers, and policy-makers. Unlike for previous analyses [23–27,42], many more trials, patients, and outcome data are now available for a comprehensive study to address this clinical question.

Given this knowledge gap, we aimed to examine the comparative effects of classes of RAS blockers in terms of cardiovascular and renal outcomes in the treatment of adult patients with diabetes mellitus. We used network meta-analyses to integrate direct and indirect evidence comparing multiple interventions of interest into unified analyses of all available randomized trials that can serve to guide evidence-based decision-making.

## Methods

This systematic review was conducted and reported in accordance with the reporting guidance provided in the Preferred Reporting Items for Systematic Reviews and Meta-Analyses (PRISMA) statement (S1 Checklist) [43,44]. We developed a systematic review protocol and registered it with PROSPERO (CRD42014014404). Our methods are briefly described here.

### Study Eligibility Criteria

Studies meeting the following selection criteria were included: randomized parallel-group controlled trials of a minimum 1-y duration; participants adults aged 18 y or older with type 1 or 2 diabetes mellitus; reporting at least one of the cardiovascular or renal outcomes of interest as well as the number of participants enrolled in each treatment arm and the number of participants with events in each treatment arm; comparison of RAS blockers in monotherapy and/or combination therapy regimens, including ACE inhibitors, ARBs, and DR inhibitors versus each other or versus placebo or other active antihypertensive treatments (e.g., calcium channel blockers [CCBs], beta blockers [BBs], or diuretics).

### Electronic Literature Search

Based on awareness of a large number of existing reviews and meta-analyses, an unlimited primary search for randomized trials was not conducted. In its place, we used a staged approach to study identification, beginning with a systematic search of relevant trials included in systematic reviews available in PubMed/MEDLINE and the Cochrane Database of Systematic Reviews (1 January 2004 to 17 July 2014) and existing meta-analyses of which we were aware (see S1 Text for details of search terms and S2 Text for references of previous reviews). PubMed/MEDLINE was next searched to identify other additional relevant trials published outside the time frames of previous reviews (1 January 2010 to 5 September 2014; reflects content from PubMed searches last conducted on 1 October 2014). We compiled a list of the unique PubMed/MEDLINE identification numbers of all relevant articles, and performed a related articles search. This technique has been shown to be effective in identifying relevant studies [45], increases efficiency in study identification in the presence of an already large evidence base, and is being used as part of an ongoing network meta-analysis research program [46]. Searches were supplemented by manual searches of clinical trial registers (including www.ClinicalTrials.gov) and review of references of relevant papers. Finally, we contacted authors of primary publications and sponsors of trials for missing outcome data or unclear information. Six drug manufacturers (Boehringer Ingelheim for ONTARGET [Ongoing Telmisartan Alone and in Combination with Ramipril Global Endpoint Trial] [40,41], PRoFESS [Prevention Regimen for Effectively Avoiding Second Strokes] [47], and TRANSCEND [Telmisartan Randomized Assessment Study in ACE-Intolerant Subjects with Cardiovascular Disease] [48,49]; Daiichi Sankio for ROADMAP [Randomized Olmesartan and Diabetes Microalbuminuria Prevention] [50]; Sanofi for IDNT [Irbesartan Diabetic Nephropathy Trial] [16] and IRMA 2 [Irbesartan in Patients with Type *2* Diabetes and Microalbuminuria] [17]; Servier for EUROPA/PERSUADE [Perindopril Substudy in Coronary Artery Disease and Diabetes] [51]; Takeda for DIRECT-Prevent 1 [52,53], DIRECT-Protect 1 [52,53], and DIRECT Protect 2 [53,54]; and Novartis for ALTITUDE [Aliskiren Trial in Type 2 Diabetes Using Cardiovascular and Renal Disease Endpoints] [22], VALUE [Valsartan Antihypertensive Long-Term Use Evaluation] [38], VALIANT [Valsartan in Acute Myocardial Infarction Trial] [39], Val-HeFT [Valsartan Heart Failure Trial] [55], and ASTRONAUT [Aliskiren Trial on Acute Heart Failure Outcomes] [56]) and four independent investigators (for ALLHAT [Antihypertensive and Lipid-Lowering Treatment to Prevent Heart Attack Trial] [20], CASE-J [Candesartan Antihypertensive Survival Evaluation in Japan] [57], COLM [Combination of Olmesartan and CCB or Low Dose Diuretics in High Risk Elderly Hypertensive Patients] [58], and OSCAR [Olmesartan and Calcium Antagonists Randomized] [59]) provided additional information about outcome data.

### Prespecified Outcome Measures

The prespecified primary endpoints were major cardiovascular outcome (composite endpoint including death from cardiovascular causes, myocardial infarction, and stroke) and progression of renal disease (composite endpoint including doubling of baseline serum creatinine level, end-stage renal disease [ESRD] defined as the need for any dialysis or renal transplantation, and all-cause mortality). We also analyzed all of the component endpoints of these two composite endpoints separately. Secondary endpoints were angina pectoris and hospitalization for heart failure. All outcomes were based on the longest follow-up period available for each included study.

### Screening, Data Extraction, and Risk of Bias Assessment

Eligible trials identified from our searching efforts were screened by one reviewer (F. Catalá-López, qualified clinical epidemiologist) and verified independently by two trained reviewers (D. Macías Saint-Gerons and C. de la Fuente-Honrubia, senior pharmacoepidemiologists). Using a predesigned form that was piloted on a small sample (14%) of studies, the same reviewers were also responsible for extraction and verification of data on general participant characteristics (e.g., average age, gender, type and duration of diabetes, level of albuminuria, presence of hypertension, presence of coronary artery disease, mean or median follow-up) and outcome data. The Cochrane risk of bias scale [60]—which considers sequence generation, allocation concealment, blinding, and other aspects of bias—was used to assess each study’s risk of bias. The overall rating of risk of bias for each study was the worst rating for any of the criteria (e.g., if any domain is scored high risk of bias, the study was considered high risk of bias). We used the Grading of Recommendations Assessment, Development, and Evaluation (GRADE) methodology [61,62] to evaluate the quality of evidence for each outcome (high, moderate, low, or very low quality). The approach we used was consistent with previous reporting of network meta-analyses [63]. Risk of bias assessments and application of GRADE methods were performed by one reviewer (F. Catalá-López) and validated by a second reviewer (D. Macías Saint-Gerons, C. de la Fuente-Honrubia. or M. Ridao). Any discrepancies between reviewers for any of the above steps were discussed until consensus was achieved.

### Data Synthesis and Analyses

Our analysis classified RAS blockers used in monotherapy and/or combination as separate treatment nodes irrespective of their doses: ACE inhibitor, ARB, ACE inhibitor combination (e.g., ACE inhibitor plus ARB, ACE inhibitor plus DR inhibitor, ACE inhibitor plus diuretic, ACE inhibitor plus CCB), ARB combination (e.g., ARB plus DR inhibitor, ARB plus diuretic, ARB plus CCB), DR inhibitor combination (e.g., DR inhibitor plus diuretic), and placebo or control (conventional therapy/usual care). In the ASTRONAUT trial [56] (aliskiren versus placebo), more than 95% of the patients with diabetes were receiving a diuretic in both groups; therefore, the diabetes subgroup analysis from this study was regarded as a trial of DR inhibitor plus diuretic versus diuretic. Similarly, the ALTITUDE trial [22] (aliskiren versus placebo) evaluated a strategy of aliskiren in patients with diabetes who were also receiving an ACE inhibitor or an ARB and was therefore regarded as a trial of DR inhibitor plus ACE inhibitor versus DR inhibitor plus ARB versus ACE inhibitor versus ARB.

Whenever possible we used results from intention-to-treat analyses. To calculate direct estimates of treatment effect for each pair of medications, we conducted pairwise random effects meta-analyses. We report the results as odds ratios (ORs) and corresponding 95% confidence intervals (CIs). We evaluated statistical heterogeneity by estimating the variance between studies with Cochran’s Q test and *I* ^2^ statistic [64,65]. The *I* ^2^ statistic is the proportion of total variation observed between the trials attributable to differences between trials rather than to sampling error. We considered *I* ^2^ < 30% as representing low statistical heterogeneity and *I* ^2^ > 75% as representing high statistical heterogeneity. Patient and study characteristics were also empirically assessed by members of the research team to establish that these were comparable across studies and comparisons. For each outcome, we present graphically the geometry of the treatment network of all comparisons using a network graph [66]. Using a Bayesian framework, we did network meta-analyses for each prespecified outcome and treatment. Network meta-analysis [67,68] allows the integration of data from both direct and indirect evidence, increasing precision while randomization is preserved, but can also be used to estimate comparisons between pairs of treatments that have not been compared in individual studies. When performing a network meta-analysis, we relied on the assumptions of transitivity [69] (i.e., if drug B is superior to drug A, and drug C is superior to drug B, it is assumed that drug C is superior to drug A) and consistency (equivalency of treatment effects from direct and indirect evidence). We used random effects network meta-analysis models as recommended elsewhere, which account for correlations in multi-arm trials [70] and which use vague (noninformative) prior distributions for all treatment effects as well as the between-study variance parameter (S3 Text). We report the results as posterior median ORs with corresponding 95% credibility intervals (CrIs), which are the Bayesian analogue of 95% CIs. The ORs reported are relative effects of multiple RAS blocker regimens. We express these using ACE inhibitor as the reference treatment, because ACE inhibitors were the first class to be developed (with the arrival of captopril and enalapril in the early 1980s) and because these agents are frequently prescribed worldwide. All network meta-analyses were based on a total of 100,000 iterations or more, with a burn-in of 50,000 iterations. We assessed convergence on the basis of Brooks–Gelman–Rubin plots [71]. The consistency of results was qualitatively examined by comparing the results obtained via pairwise meta-analyses versus network meta-analyses. Consistency was also examined by fitting both consistency and inconsistency models [72] for network meta-analysis and comparing the deviance information criterion (DIC) between models, with smaller values indicative of a better fit, and a difference of five or more being considered as important. In general, when both models had a similar fit to the data as indicated by their DIC values, we concluded that there was no evidence of inconsistency. We also used the surface under the cumulative ranking curve (SUCRA) to potentially rank the treatments [73].

A priori sensitivity analyses were conducted to explore potentially important effect modifiers. These included separate analyses that involved exclusion of the following: studies with high risk of bias, small studies with fewer than 100 patients, studies in which >50% patients presented type 1 diabetes, and studies in non-hypertensive patients. Other preplanned analyses were the extension of the primary unadjusted network meta-analysis model to include covariates in meta-regression models [74] that considered adjustments for the following: year of publication, mean age of trial participants, percent of male participants, and control group risk rate. Considering that follow-up times varied between the included trials, we also estimated the rate ratios based on patient-years and corresponding 95% CrIs. All pairwise meta-analyses were conducted using Stata 13 (StataCorp, College Station, Texas, US), while Bayesian network meta-analyses were performed using WinBUGS 1.4.3 (MRC Biostatistics Unit, Cambridge, UK).

## Results

### Overview of Study Selection and Study Characteristics

We included 71 studies (described in 88 publications) that satisfied our inclusion criteria (PRISMA flowchart is shown in S1 Fig; details of the included studies are shown in S4 Text and S1–S7 Tables), with 103,120 individuals randomly assigned to one of the study treatments. The median duration of follow-up of the set of included studies was 3.2 y (range 1.0–9.0), and the median sample size was 436 individuals (range 30–13,168), with 55 trials having at least 100 participants and 24 trials having more than 1,000. Fifty-four studies (76%) were two-arm trials, 12 (17%) were three-arm trials involving three active agents or two active agents and placebo, and four (6%) were analyzed as multi-arm trials with four different active comparisons or three active agents and placebo. In terms of methodological quality and potential risk of bias (S2 and S3 Tables), 37 trials (52%) had an unclear risk of bias for at least one criterion, 25 trials (35%) had a low risk of bias, and nine trials (13%) had a high risk of bias. Fifty trials (70%) were funded by drug companies.

The characteristics of patients amongst the included studies are described in S4 Table. At baseline, the median age was 60 y, the median percentage of male participants was 61%, and the median duration of diabetes was 10.3 y. Forty-seven studies (63%) targeted type 2 diabetes, (5%) enrolled patients with type 1 diabetes, and ten studies (32%) contained mixed populations with type 1 or type 2 diabetes. In terms of level of albuminuria, 15 trials enrolled patients with macroalbuminuria, 13 enrolled patients with microalbuminuria, seven enrolled patients with normoalbuminuria, and 25 trials enrolled mixed populations. Approximately half of all studies (37 trials) included patients with hypertension at baseline, 15 trials enrolled normotensive participants, and 19 trials enrolled mixed populations. In terms of history of coronary disease, 58% of all studies (41 trials) were rated as unclear or not reported, and 27 trials were rated as having a mixed population (e.g., 5%–75% of participants with coronary disease at baseline).

**Fig 1:**
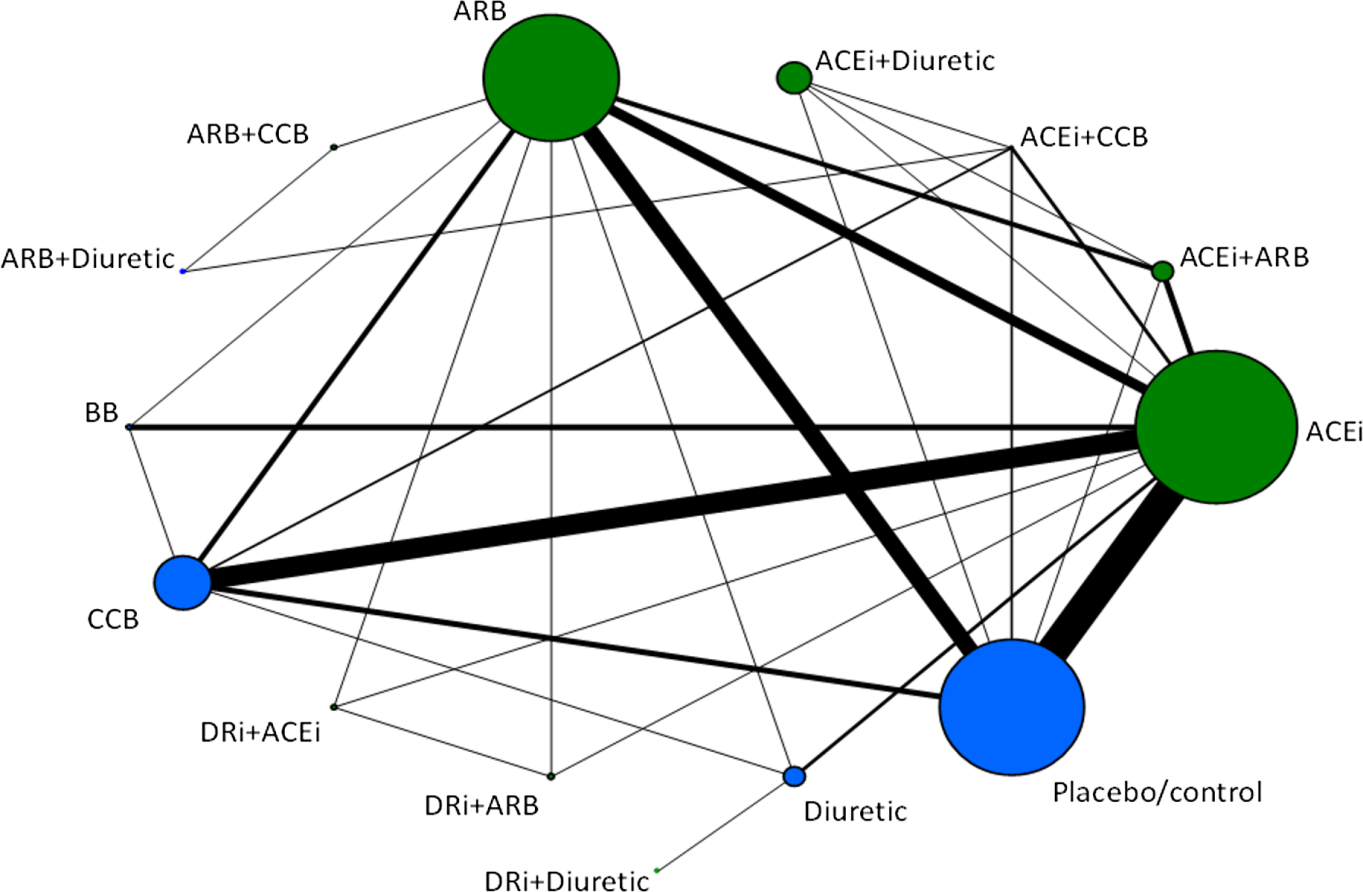
Evidence network of all treatment comparisons for all studies. Lines represent direct comparisons within randomized controlled trials. The size of nodes is proportional to the number of randomized participants (sample size), and the width of the lines is proportional to the number of trials comparing each pair of treatments. Nodes in green represent RAS blockers (in monotherapy and/or combination therapies). Nodes in blue represent other control arms included in the evidence networks to preserve randomization. ACEi, ACE inhibitor; DRi, DR inhibitor.

The structure of the underlying evidence base for each analysis is described in Table 1 and Fig 1. The studies reporting data for each clinical outcome of interest are summarized in Table 1 (and S4–S7 Tables). Figs 1 and S2 show the evidence networks of eligible treatment comparisons for the full network meta-analysis. ACE inhibitors, placebo use or control (conventional therapy/usual care), and ARBs were most investigated (49, 36, and 25 trials, respectively). Treatments of interest in the main analyses were any RAS blocker compared with another RAS blocker or placebo or control. Other antihypertensive control arms (e.g., CCBs, BBs, or diuretics) were included in the evidence networks in order to incorporate additional indirect evidence for the analyses while preserving randomization. The results for these alternative antihypertensive treatments are presented in S8 Table.

**Table 1:** Availability of data for principal analyses.

Treatment	Data Available by Outcome									
	Major Cardiovascular Outcome	Cardiovascular Mortality	Myocardial Infarction	Stroke	Angina Pectoris	Hospitalization for Heart Failure	Progression of Renal Disease	ESRD	Doubling of CrS	All-Cause Mortality
**ACEi**										
Number of trials	17	25	30	23	16	18	7	14	13	39
Number of participants	16,692	17,601	17,730	17,446	14,307	17,382	11,933	13,382	11,818	19,165
Number of events	2,607	1,394	1,153	835	1,340	1,211	1,900	278	429	2,522
**ARB**										
Number of trials	18	18	21	22	13	18	12	9	11	22
Number of participants	21,792	21,788	23,111	23,422	15,738	21,125	14,726	12,276	14,002	23,249
Number of events	2,772	1,335	1,005	1,041	1,125	1,274	1,712	455	633	2,277
**ACEi + ARB**										
Number of trials	3	3	5	5	4	6	3	3	2	6
Number of participants	4,571	4,571	5,322	5,322	5,180	5,930	4,149	4,149	3,425	5,931
Number of events	913	568	413	229	612	679	797	137	166	999
**DRi + ACEi**										
Number of trials	1	1	1	1	0	1	1	1	1	1
Number of participants	1,926	1,926	1,926	1,926	0	1,926	1,926	1,926	1,926	1,926
Number of events	234	136	84	65	0	106	108	53	82	202
**DRi + ARB**										
Number of trials	1	1	1	1	0	1	1	1	1	1
Number of participants	2,353	2,353	2,353	2,353	0	2,353	2,353	2,353	2,353	2,353
Number of events	224	115	64	86	0	103	155	69	134	179
**DRi + diuretic**										
Number of trials	1	1	1	1	1	1	0	0	1	1
Number of participants	319	319	319	319	319	319	0	0	319	319
Number of events	69	62	11	6	16	108	0	0	65	72
**ACEi + diuretic**										
Number of trials	1	1	1	2	0	1	1	1	1	3
Number of participants	5,569	5,569	27	5,595	0	27	5,569	5,569	5,569	5,761
Number of events	480	211	0	288	0	2	488	25	55	411
**ARB + diuretic**										
Number of trials	1	1	2	1	1	1	1	1	1	1
Number of participants	678	678	734	678	678	678	678	678	678	678
Number of events	26	7	9	16	2	5	8	3	6	29
**ACEi + CCB**										
Number of trials	1	3	2	1	0	0	0	0	0	4
Number of participants	104	530	158	104	0	0	0	0	0	696
Number of events	4	1	2	1	0	0	0	0	0	5
**ARB + CCB**										
Number of trials	1	2	2	2	2	2	1	1	1	2
Number of participants	684	1,003	1,003	1,003	1,003	1,003	684	684	684	1,003
Number of events	26	5	6	34	3	10	8	0	8	29
**Placebo/control**										
Number of trials	17	19	19	18	13	14	11	10	17	31
Number of participants	21,798	21,998	16,097	21,813	11,848	14,987	16,118	12,507	16,533	22,785
Number of events	2,580	1,094	747	1,138	806	607	1,880	408	629	1,976

There were also trials involving comparisons with other control arms (diuretics, CCBs, and/or BBs in monotherapy), which were included in the evidence networks to preserve randomization. ACEi, ACE inhibitor; CrS, serum creatinine; DRi, DR inhibitor.

### Findings from Network Meta-Analysis

Summaries from all network meta-analyses are provided below for each outcome.

Major cardiovascular outcome (composite) was reported in 12,328 of 92,469 patients from a total of 33 studies. With placebo use as the reference, ACE inhibitors (OR 0.87; 95% CrI 0.75–0.99; moderate confidence) significantly reduced the risk of major cardiovascular outcome. Compared with ACE inhibitor as the reference treatment, none of the remaining RAS blockers used in monotherapy or combination were associated with significant risk reductions: ARB (1.02; 95% CrI 0.90–1.18; moderate confidence), ACE inhibitor plus ARB (0.97; 95% CrI 0.79–1.19; moderate confidence), DR inhibitor plus ARB (1.00; 95% CrI 0.73–1.38; low confidence), and DR inhibitor plus ACE inhibitor (1.32; 95% CrI 0.96–1.81; low confidence) (Tables 2, 3, and S8).

**Table 2:** Cardiovascular outcomes: comparisons of random effects pairwise meta-analysis with Bayesian network meta-analysis, including confidence of assessments.

Outcome	Treatment	Number of Studies with Direct Comparison to ACEi (Participants)	Pairwise Meta-analysis OR (95% CI); Quality of Evidence	*I* ^2^ (95% CI)	Cochran’s Q *p*-Value	Network Meta-analysis OR (95% CrI); Quality of Evidence
**Major cardiovascular outcome**	ACEi (reference)					
	ARB	5 (13,480)	0.93 (0.77–1.13); Moderate	62.3% (0.0–82.0)	0.03	1.02 (0.90–1.18); Moderate
	ACEi + ARB	3 (9,046)	0.95 (0.85–1.05); Moderate	0.0% (0.0–90.0)	0.85	0.97 (0.79–1.19); Moderate
	DRi + ACEi	1 (3,790)	1.09 (0.90–1.34); Low	—	—	1.32 (0.96–1.81); Low
	DRi + ARB	1 (4,217)	0.83 (0.68–1.02); Low	—	—	1.00 (0.73–1.38); Low
	DRi + diuretic	0 (0)	—	—	—	1.07 (0.63–1.91); Very low
	ACEi + diuretic	0 (0)	—	—	—	1.05 (0.75–1.52); Very low
	ACEi + CCB	1 (206)	0.54 (0.15–1.91); Very low	—	—	0.47 (0.12–1.41); Very low
**Cardiovascular mortality**	ACEi (reference)					
	ARB	5 (13,480)	0.97 (0.78–1.23): Moderate	60.7% (0.0–85.0)	0.04	1.00 (0.82–1.23); Moderate
	ACEi + ARB	3 (9,046)	1.03 (0.86–1.22); Moderate	29.6% (0.0–93.0)	0.24	1.06 (0.79–1.49); Moderate
	DRi + ACEi	1 (3,790)	1.18 (0.91–1.52); Low	—	—	1.45 (0.91–2.35); Low
	DRi + ARB	1 (4,217)	0.80 (0.61–1.04); Low	—	—	0.98 (0.61–1.60); Low
	DRi + diuretic	0 (0)	—	—	—	1.32 (0.63–2.84); Very low
	ACEi + diuretic	0 (0)	—	—	—	0.81 (0.49–1.36); Very low
	ARB + diuretic	0 (0)	—	—	—	0.71 (0.02–14.19); Very low
	ACEi + CCB	1 (206)	0.49 (0.04–5.44); Very low	—	—	0.15 (0.01–0.88); Very low
	ARB + CCB	0 (0)	—	—	—	0.41 (0.02–5.75); Very low
**Myocardial infarction**	ACEi (reference)					
	ARB	5 (13,480)	0.98 (0.78–1.23); Moderate	48.3% (0.0–81.0)	0.10	1.07 (0.89–1.28); Moderate
	ACEi + ARB	3 (9,046)	0.94 (0.81–1.10); Moderate	0.0% (0.0–90.0)	0.69	1.00 (0.78–1.33); Moderate
	DRi + ACEi	1 (3,790)	1.10 (0.80–1.52); Low	—	—	1.35 (0.85–2.12); Low
	DRi + ARB	1 (4,217)	0.68 (0.48–0.95); Low	—	—	0.83 (0.52–1.31); Low
	DRi + diuretic	0 (0)	—	—	—	0.61 (0.24–1.60); Very low
	ARB + diuretic	0 (0)	—	—	—	3.06 (0.37–39.80); Very low
	ACEi + CCB	1 (206)	0.32 (0.03–3.13); Very low	—	—	0.45 (0.06–2.18); Very low
	ARB + CCB	0 (0)	—	—	—	1.45 (0.21–14.40); Very low
**Stroke**	ACEi (reference)					
	ARB	6 (13,522)	0.93 (0.79–1.09); Moderate	0.0% (0.0–75.0)	0.96	1.01 (0.88–1.16); Moderate
	ACEi + ARB	4 (9,100)	0.86 (0.71–1.04); Moderate	0.0% (0.0–85.0)	0.58	0.88 (0.71–1.08); Moderate
	DRi + ACEi	1 (3,790)	1.09 (0.76–1.56); Low	—	—	1.19 (0.82–1.67); Low
	DRi + ARB	1 (4,217)	1.18 (0.84–1.66); Low	—	—	1.28 (0.91–1.78); Low
	DRi + diuretic	0 (0)	—	—	—	0.45 (0.16–1.20); Very low
	ACEi + diuretic	0 (0)	—	—	—	1.05 (0.81–1.41); Very low
	ARB + diuretic	0 (0)	—	—	—	0.75 (0.19–2.90); Very low
	ACEi + CCB	1 (206)	0.32 (0.03–3.13); Very low	—	—	0.31 (0.01–2.30); Very low
	ARB + CCB	0 (0)	—	—	—	1.21 (0.40–3.67); Very low
**Angina pectoris**	ACEi (reference)					
	ARB	3 (9,046)	1.03 (0.80–1.31); Moderate	57.0% (0.0–88.0)	0.10	1.14 (0.98–1.37); Moderate
	ACEi + ARB	4 (10,231)	0.98 (0.79–1.21); Moderate	48.5% (0.0–83.0)	0.12	1.00 (0.82–1.23); Moderate
	DRi + ACEi	0 (0)	—	—	—	NA
	DRi + ARB	0 (0)	—	—	—	NA
	DRi + diuretic	0 (0)	—	—	—	1.32 (0.56–3.17); Very low
	ACEi + diuretic	0 (0)	—	—	—	NA
	ARB + diuretic	0 (0)	—	—	—	9.81 (0.27–564.0); Very low
	ACEi + CCB	0 (0)	—	—	—	NA
	ARB + CCB	0 (0)	—	—	—	3.25 (0.28–89.45); Very low
**Hospitalization for heart failure**	ACEi (reference)					
	ARB	5 (13,480)	0.99 (0.81–1.21); Moderate	48.1% (0.0–81.0)	0.10	0.99 (0.86–1.12); Moderate
	ACEi + ARB	5 (10,284)	0.91 (0.80–1.03); Moderate	0.0% (0.0–79.0)	0.71	0.86 (0.73–1.01); Moderate
	DRi + ACEi	1 (3,790)	0.91 (0.69–1.20); Low	—	—	1.06 (0.75–1.45); Low
	DRi + ARB	1 (4,217)	0.72 (0.54–0.94); Low	—	—	0.83 (0.59–1.14); Low
	DRi + diuretic	0 (0)	-	—	—	1.05 (0.65–1.70); Very low
	ACEi + diuretic	1 (54)	5.39 (0.25–117.77); Very low	—	—	2.02 (0.22–22.89); Very low
	ARB + diuretic	0 (0)	—	—	—	0.37 (0.05–2.43); Very low
	ACEi + CCB	0 (0)	—	—	—	NA
	ARB + CCB	0 (0)	—	—	—	0.55 (0.10–2.23); Very low

An OR > 1 favors the ACE inhibitor (i.e., fewer events occur with the ACE inhibitor than with the other active agent).

ACEi, ACE inhibitor; DRi, DR inhibitor; NA, not applicable.

**Table 3:** Major cardiovascular outcome (composite endpoint) and all possible treatment comparisons.

**Pbo/Control**													
0.87 (0.75–0.99)	**ACEi**												
1.12 (0.81–1.54)	1.29 (0.95–1.76)	**BB**											
0.81 (0.67–0.97)	0.93 (0.79–1.12)	0.72 (0.52–1.02)	**CCB**										
0.89 (0.78–1.01)	1.02 (0.90–1.18)	0.80 (0.59–1.09)	1.10 (0.93–1.30)	**ARB**									
0.41 (0.11–1.22)	0.47 (0.12–1.41)	0.36 (0.09–1.14)	0.50 (0.13–1.51)	0.46 (0.12–1.37)	**ACEi + CCB**								
0.92 (0.69–1.28)	1.06 (0.82–1.47)	0.82 (0.56–1.28)	1.14 (0.87–1.55)	1.03 (0.79–1.43)	2.27 (0.74–8.95)	**Diuretic**							
0.91 (0.66–1.26)	1.05 (0.75–1.52)	0.82 (0.52–1.30)	1.13 (0.78–1.64)	1.03 (0.73–1.46)	2.26 (0.72–8.94)	1.00 (0.62–1.51)	**ACEi + Diuretic**						
NA	NA	NA	NA	NA	NA	NA	NA	**ARB + Diuretic**					
0.84 (0.67–1.05)	0.97 (0.79–1.19)	0.75 (0.53–1.08)	1.04 (0.80–1.33)	0.94 (0.77–1.16)	2.07 (0.67–7.94)	0.91 (0.63–1.26)	0.92 (0.62–1.35)	NA	**ACEi + ARB**				
1.15 (0.82–1.58)	1.32 (0.96–1.81)	1.02 (0.67–1.57)	1.41 (0.99–1.98)	1.29 (0.93–1.75)	2.81 (0.90–11.22)	1.25 (0.79–1.84)	1.25 (0.78–1.96)	NA	1.37 (0.94–1.95)	**DRi + ACEi**			
0.87 (0.62–1.20)	1.00 (0.73–1.38)	0.78 (0.51–1.20)	1.08 (0.75–1.51)	0.98 (0.71–1.33)	2.15 (0.69–8.45)	0.95 (0.60–1.40)	0.95 (0.59–1.50)	NA	1.04 (0.72–1.48)	0.76 (0.54–1.08)	**DRi + ARB**		
0.93 (0.54–1.66)	1.07 (0.63–1.91)	0.83 (0.45–1.60)	1.14 (0.67–2.03)	1.04 (0.60–1.86)	2.29 (0.67–9.73)	1.00 (0.63–1.62)	1.01 (0.54–1.97)	NA	1.10 (0.62–2.02)	0.81 (0.44–1.56)	0.58 (0.00–1.06)	**DRi + Diuretic**	
NA	NA	NA	NA	NA	NA	NA	NA	0.99 (0.52–1.84)	NA	NA	NA	NA	**ARB + CCB**

Summary ORs and CrIs from network meta-analysis. Comparisons between treatments should be read from left to right. The treatment at the top of each column is the reference group for comparisons in that column, while the comparator is the lower treatment in the stepladder. For major cardiovascular outcome, OR < 1 suggests fewer outcomes with the comparator than with the reference group. Effect sizes for treatment comparisons including ARB + diuretic and/or ARB + CCB were generally unstable and uninterpretable owing to small sample sizes, rare events, and lack of direct evidence.

ACEi, ACE inhibitor; DRi, DR inhibitor; NA, not available; Pbo/Control, placebo or control.

Progression of renal disease (composite endpoint of ESRD, doubling of serum creatinine, and all-cause mortality) occurred in 9,267 of 69,380 patients from 18 studies with available data. No RAS blocker was found to reduce progression of renal disease compared to placebo. Compared with ACE inhibitor as the reference treatment, no RAS blocker used in monotherapy and/or combination was associated with any significant reduction of progression of renal disease. Compared with ACE inhibitor as the reference, ORs were 1.10 (95% CrI 0.90–1.40; moderate confidence) for ARB, 0.97 (95% CrI 0.72–1.29; moderate confidence) for ACE inhibitor plus ARB, 0.99 (95% CrI 0.65–1.57; low confidence) for DR inhibitor plus ACE inhibitor, and 1.18 (95% CrI 0.78–1.84; low confidence) for DR inhibitor plus ARB (Tables 4, 5, and S8).

**Table 4:** Renal outcomes and all-cause mortality: comparisons of random effects pairwise meta-analysis with Bayesian network meta-analysis, including confidence of assessments.

Outcome	Treatment	Number of Studies with Direct Comparison to ACEi (Participants)	Pairwise Meta-analysis OR (95% CI); Quality of Evidence	*I* ^2^ (95% CI)	Cochran’s Q *p*-Value	Network Meta-analysis OR (95% CrI); Quality of Evidence
**Progression of renal disease**	ACEi (reference)					
	ARB	3 (10,976)	1.13 (0.89–1.42); Moderate	56.7% (0.0–88.0)	0.10	1.10 (0.90–1.40); Moderate
	ACEi + ARB	2 (6,780)	1.04 (0.92–1.18); Moderate	0.0% (0.0–90.0)	0.43	0.97 (0.72–1.29); Moderate
	DRi + ACEi	1 (3,790)	1.11 (0.83–1.47); Low	—	—	0.99 (0.65–1.57); Low
	DRi + ARB	1 (4,217)	1.31 (1.01–1.71); Low	—	—	1.18 (0.78–1.84); Low
	DRi + diuretic	0 (0)	—	—	—	NA
	ACEi + diuretic	0 (0)	—	—	—	0.98 (0.61–1.62); Very low
	ARB + diuretic	0 (0)	—	—	—	NA
	ACEi + CCB	0 (0)	—	—	—	NA
	ARB + CCB	0 (0)	—	—	—	NA
**ESRD**	ACEi (reference)					
	ARB	3 (10,976)	1.14 (0.76–1.71); Moderate	62.1% (0.0–0.89)	0.07	1.09 (0.84–1.42); Moderate
	ACEi + ARB	2 (6,780)	0.99 (0.75–1.32); Moderate	0.0% (0.0–90.0)	0.98	0.91 (0.63–1.27); Moderate
	DRi + ACEi	1 (3,790)	1.20 (0.80–1.80); Low	—	—	1.09 (0.66–1.85); Low
	DRi + ARB	1 (4,217)	1.28 (0.87–1.88); Low	—	—	1.17 (0.72–1.93); Low
	DRi + diuretic	0 (0)	—	—	—	NA
	ACEi + diuretic	0 (0)	—	—	—	1.75 (0.81–3.80); Very low
	ARB + diuretic	0 (0)	—	—	—	NA
	ACEi + CCB	0 (0)	—	—	—	NA
	ARB + CCB	0 (0)	—	—	—	NA
**Doubling of serum creatinine**	ACEi (reference)					
	ARB	3 (10,976)	1.20 (0.99–1.45); Moderate	0.0% (0.0–90.0)	0.39	1.26 (0.97–1.79); Moderate
	ACEi + ARB	2 (6,780)	0.99 (0.78–1.25); Moderate	0.0% (0.0–90.0)	0.45	0.99 (0.65–1.56); Moderate
	DRi + ACEi	1 (3,790)	0.98 (0.71–1.34); Low	—	—	0.93 (0.53–1.74); Low
	DRi + ARB	1 (4,217)	1.33 (1.00–1.76); Low	—	—	1.27 (0.73–2.35); Low
	DRi + diuretic	0 (0)	—	—	—	1.81 (0.72–4.62); Very low
	ACEi + diuretic	0 (0)	—	—	—	1.75 (0.84–3.84); Very low
	ARB + diuretic	0 (0)	—	—	—	NA
	ACEi + CCB	0 (0)	—	—	—	NA
	ARB + CCB	0 (0)	—	—	—	NA
**All-cause mortality**	ACEi (reference)					
	ARB	6 (13,670)	0.96 (0.86–1.08); Moderate	12.1% (0.0–78.0)	0.34	1.04 (0.92–1.18); Moderate
	ACEi + ARB	4 (10,231)	1.03 (0.93–1.14); Moderate	0.0% (0.0–85.0)	0.93	1.03 (0.88–1.21); Moderate
	DRi + ACEi	1 (3,790)	1.12 (0.91–1.39); Low	—	—	1.29 (0.96–1.72); Low
	DRi + ARB	1 (4,217)	0.79 (0.63–0.98); Low	—	—	0.90 (0.67–1.21); Low
	DRi + diuretic	0 (0)	—	—	—	1.53 (0.91–2.76); Very low
	ACEi + diuretic	1 (53)	3.24 (0.13–83.03); Very low	—	—	0.90 (0.67–1.26); Very low
	ARB + diuretic	0 (0)	—	—	—	1.19 (0.35–3.97); Very low
	ACEi + CCB	0 (0)	—	—	—	0.47 (0.13–1.14); Very low
	ARB + CCB	0 (0)	—	—	—	0.88 (0.31–2.47); Very low

An OR > 1 favors the ACE inhibitor (i.e., fewer events occur with the ACE inhibitor than with the other active agent).

ACEi, ACE inhibitor; DRi, DR inhibitor; NA, not applicable.

**Table 5:** Progression of renal disease (composite outcome) and all possible treatment comparisons.

**Pbo/Control**													
0.92(0.73–1.14)	**ACEi**												
9.00(2.15–41.83)	9.80 (3.37–45.05)	**BB**											
1.08(0.81–1.50)	1.17(0.90–1.64)	0.12(0.03–0.50)	**CCB**										
1.01(0.84–1.24)	1.10(0.90–1.40)	0.11(0.02–0.47)	0.93(0.69–1.24)	**ARB**									
NA	NA	NA	NA	NA	**ACEi + CCB**								
1.05(0.69–1.63)	1.14(0.78–1.74)	0.12(0.02–0.50)	0.98(0.64–1.43)	1.04(0.67–1.59)	NA	**Diuretic**							
0.90(0.58–1.39)	0.98(0.61–1.62)	0.10(0.02–0.44)	0.83(0.48–1.39)	0.89(0.54–1.42)	NA	0.85(0.46–1.57)	**ACEi + Diuretic**						
NA	NA	NA	NA	NA	NA	NA	NA	**ARB + Diuretic**					
0.90(0.65–1.19)	0.97(0.72–1.29)	0.10(0.02–0.42)	0.83(0.54–1.17)	0.89(0.66–1.13)	NA	0.85(0.51–1.34)	1.00(0.57–1.66)	NA	**ACEi + ARB**				
0.91(0.58–1.45)	0.99(0.65–1.57)	0.10(0.02–0.45)	0.84(0.50–1.38)	0.90(0.58–1.39)	NA	0.86(0.48–1.56)	1.01(0.54–1.92)	NA	1.02(0.63–1.71)	**DRi + ACEi**			
1.08(0.69–1.70)	1.18(0.78–1.84)	0.12(0.02–0.53)	1.00(0.60–1.62)	1.07(0.70–1.63)	NA	1.03(0.58–1.84)	1.20(0.65–2.27)	NA	1.21(0.76–2.02)	1.19(0.73–1.92)	**DRi + ARB**		
NA	NA	NA	NA	NA	NA	NA	NA	NA	NA	NA	NA	**DRi + Diuretic**	
NA	NA	NA	NA	NA	NA	NA	NA	0.99(0.33–2.98)	NA	NA	NA	NA	**ARB + CCB**

Summary ORs and CrIs from network meta-analysis. Comparisons between treatments should be read from left to right. The treatment at the top of each column is the reference group for comparisons in that column, while the comparator is the lower treatment in the stepladder. For progression of renal disease, OR < 1 suggests fewer outcomes with the comparator than with the reference group. Effect sizes for treatment comparisons including ARB + diuretic and/or ARB + CCB were generally unstable and uninterpretable owing to small sample sizes, rare events, and lack of direct evidence. There were no studies evaluating ACE inhibitor + CCB or DR inhibitor + diuretic.

ACEi, ACE inhibitor; DRi, DR inhibitor; NA, not available; Pbo/Control, placebo or control.

Cardiovascular mortality occurred in 6,166 of 95,060 patients from 41 studies that provided data. With placebo as the reference treatment, ACE inhibitor plus CCB (OR 0.14; 95% CrI 0.01–0.83) appeared to reduce the risk of cardiovascular mortality, though with very low confidence. Compared with ACE inhibitor as the reference treatment (Tables 2 and S8), no RAS blocker (except ACE inhibitor plus CCB) was associated with a significant reduction in cardiovascular mortality: ARB (1.07; 95% CrI 0.88–1.33; moderate confidence), ACE inhibitor plus ARB (1.06; 95% CrI 0.79–1.49; moderate confidence), DR inhibitor plus ARB (0.98; 95% CrI 0.61–1.60; low confidence), and DR inhibitor plus ACE inhibitor (1.45; 95% CrI 0.91–2.35; low confidence).

Myocardial infarction occurred in 4,593 of 84,792 patients from 48 studies with available data. Both ACE inhibitors (OR 0.77; 95% CrI 0.62–0.92; moderate confidence) and ARBs (0.82; 95% CrI 0.67–0.98; moderate confidence) were associated with reduced risk of myocardial infarction compared with placebo. Compared with ACE inhibitor as the reference treatment (Tables 2 and S8), no RAS blocker used in monotherapy and/or combination was associated with a significant reduction in myocardial infarction: ARB (1.07; 95% CrI 0.89–1.28; moderate confidence), ACE inhibitor plus ARB (1.00; 95% CrI 0.78–1.33; moderate confidence), DR inhibitor plus ARB (0.83; 95% CrI 0.52–1.31; low confidence), and DR inhibitor plus ACE inhibitor (1.35; 95% CrI 0.85–2.12; low confidence).

Stroke was observed in 4,591 of 95,155 patients from 42 studies. The combination of ACE inhibitor plus ARB was associated with a reduced risk of stroke compared with placebo (OR 0.80; 95% CrI 0.62–0.98; moderate confidence). Compared with ACE inhibitor as the reference treatment (Tables 2 and S8), no RAS blocker used in monotherapy and/or combination was associated with a significant reduction in the incidence of stroke: ARB (1.01; 95% CrI 0.88–1.16; moderate confidence), ACE inhibitor plus ARB (0.88; 95% CrI 0.71–1.08; moderate confidence). Combinations of DR inhibitor with ACE inhibitor/ARB were potentially associated with an increased risk of stroke: DR inhibitor plus ARB compared with ACE inhibitor plus ARB (1.44; 95% CrI 1.00–2.13; low confidence; S8 Table).

ESRD was reported in 1,786 of 67,316 patients from the 22 studies with available data. ACE inhibitors (OR 0.68; 95% CrI 0.51–0.91; moderate confidence), ARBs (0.74; 95% CrI 0.57–0.97; moderate confidence), and combinations of ACE inhibitor plus ARB (0.62; 95% CrI 0.42–0.90; moderate confidence) were associated with a reduced risk of ESRD compared with placebo. Compared with ACE inhibitor as the reference treatment (Tables 4 and S8), no RAS blocker used in monotherapy and/or combination was associated with a significant reduction in ESRD: ARB (1.09; 95% CrI 0.84–1.42; moderate confidence), ACE inhibitor plus ARB (0.91; 95% CrI 0.63–1.27; moderate confidence), DR inhibitor plus ACE inhibitor (1.09; 95% CrI 0.66–1.85; low confidence), and DR inhibitor plus ARB (1.17; 95% CrI 0.72–1.93; low confidence).

Doubling of serum creatinine level was reported in 2,645 of 67,505 patients from 24 studies. With placebo as the reference treatment, ACE inhibitors (OR 0.70; 95% CrI 0.52–0.91; moderate confidence) were associated with important reductions in the risk of doubling of serum creatinine. Compared with ACE inhibitor as the reference treatment (Tables 4 and S8), no RAS blocker used in monotherapy and/or combination was associated with a significant reduction of doubling of serum creatinine: ARB (1.26; 95% CrI 0.97–1.79; moderate confidence), ACE inhibitor plus ARB (0.99; 95% CrI 0.65–1.56; moderate confidence), DR inhibitor plus ARB (1.27; 95% CrI 0.73–2.35; low confidence), and DR inhibitor plus ACE inhibitor (0.93; 95% CrI 0.53–1.74; low confidence).

Death from any cause was reported in 11,199 of 101,369 patients from 59 studies. Compared with placebo or with ACE inhibitor as the reference treatment (S8 Table), no RAS blocker used in monotherapy and/or combination was associated with a significant reduction in all-cause mortality. Compared with ACE inhibitor as the reference treatment (Tables 4 and S8), the ORs were 1.04 (95% CrI 0.92–1.18; moderate confidence) for ARB, 1.03 (95% CrI 0.88–1.21; moderate confidence) for ACE inhibitor plus ARB, 1.29 (95% CrI 0.96–1.72; low confidence) for DR inhibitor plus ACE inhibitor, and 0.90 (95% CrI 0.67–1.21; low confidence) for DR inhibitor plus ARB.

Angina pectoris was reported in 5,026 of 65,656 patients from 30 studies. Both ACE inhibitors (0.81; 95% CrI 0.65–0.96; moderate confidence) and ARBs (0.76; 95% CrI 0.61–0.98; moderate confidence) were associated with a reduced risk of angina compared with placebo. Compared with ACE inhibitor as the reference treatment (Tables 2 and S8), no RAS blocker used in monotherapy and/or combination was associated with a significant reduction in angina pectoris: ARB (1.14; 95% CrI 0.98–1.37; moderate confidence), ACE inhibitor plus ARB (1.00; 95% CrI 0.82–1.23; moderate confidence), and DR inhibitor plus diuretic (1.32; 95% CrI 0.56–3.17; very low confidence).

Hospitalization for heart failure was reported in 5,272 of 81,373 patients from 33 studies. With placebo as reference treatment, ARB alone or in combination with ACE inhibitor seemed to be associated with a reduced risk of hospitalization for heart failure: 0.86 (95% CrI 0.72–0.99; moderate confidence) for ARB and 0.75 (95% CrI 0.60–0.91; moderate confidence) for ACE inhibitor plus ARB. Because of borderline estimates, ACE inhibitors were potentially associated with a decreased risk of heart failure hospitalization compared with placebo: 0.86 (95% CrI 0.73–1.01; moderate confidence). Compared with ACE inhibitor as the reference treatment (Tables 2 and S8), no RAS blocker used in monotherapy and/or combination was associated with a significant reduction in heart failure hospitalization: ARB (0.99; 95% CrI 0.86–1.12; moderate confidence), ACE inhibitor plus ARB (0.86; 95% CrI 0.73–1.01; moderate confidence), DR inhibitor plus ACE inhibitor (1.06; 95% CrI 0.75–1.45; low confidence), and DR inhibitor plus ARB (0.83; 95% CrI 0.59–1.14; low confidence).

### Additional Analyses and Evaluation of Models

A summary of SUCRA values with 95% CrIs by treatment and outcome is reported in S9 Table. Overall, many of these estimates were imprecise and do not allow for firm conclusions to be drawn. The full details of the sensitivity analyses are reported in S10 Table. For each outcome, our findings were robust when we removed studies with high risk of bias, small studies, type 1 diabetes mellitus studies, and studies in normotensive patients. We note that the results were not materially different when we examined the distribution of potential effect modifiers by including covariates in network meta-regression models or when estimating the rate ratios based on patient-years. Because of the small number of studies with available data, we were unable to perform preplanned meta-regression analysis to evaluate the impact of history of coronary disease. There was generally a better trade-off between model fit and complexity when consistency was assumed than when it was not (S11 Table). Potential significant inconsistency was identified in a small number of cases: for stroke (consistency model DIC = 581.58 versus inconsistency model DIC = 589.80) and for angina pectoris (407.85 versus 414.21). Data were double-checked, and we could not identify any important effect modifier that differed across comparisons.

## Discussion

In our main analyses, we found no significant differences in the risk of major cardiovascular outcome (composite of cardiovascular death, myocardial infarction, and stroke) between ACE inhibitor and either ARB or the combination of ACE inhibitor plus ARB. For the risk of progression of renal disease (composite of ESRD, doubling of serum creatinine, and all-cause mortality), no significant differences were detected between ACE inhibitor and any of the remaining therapies, such as ARB or the combination of ACE inhibitor plus ARB. Our findings also suggest that no RAS blocker strategy was superior to ACE inhibitor with respect to all-cause mortality, cardiovascular mortality, myocardial infarction, ESRD, or doubling of serum creatinine. Evidence can now be drawn from data for over 103,000 adults with diabetes from 71 randomized trials with at least 1 y of follow-up, and this increased sample size can provide more precise estimates of the cardiovascular and renal effects. We believe it is unlikely that future trials will show clinically relevant advantages of ARBs over ACE inhibitors (and vice versa) in preventing cardiovascular outcomes in adults with diabetes mellitus. For renal outcomes, some may consider, however, the necessity for additional industry-independent trials providing specific comparative data for the renoprotective effects of RAS blockade in patients with diabetic kidney disease. Despite many systematic reviews evaluating RAS blockers [23–37], to the best of our knowledge, this is the most comprehensive published systematic review (and network meta-analysis) of comparative treatment effects associated with the use of RAS blockers. It fully integrates all major cardiorenal outcomes and studies assessing ACE inhibitors, ARBs, and the DR inhibitor aliskiren in adults with diabetes, thereby providing clinicians and patients with an overall appraisal of these therapies. Compared with previous reviews in diabetes mellitus [23–26] (S12–S14 Tables), our analyses incorporate between more than two and five times the number of study participants and events. Our review was prospectively registered in PROSPERO and also adheres to the recently developed PRISMA reporting standards for network meta-analyses (S1 Checklist) [44]. Our main conclusions differed from those reported in recent meta-analyses in adults with diabetes, which claim different treatment effects for classes of RAS blockers [23,24].

Unlike previous reviews, which were limited to analyzing data only from published randomized trials, we were able to include unpublished data from large trials and/or specific data for subgroups of patients with diabetes [20,39–41,47–49]. Our network meta-analyses considered both direct and indirect comparisons of multiple antihypertensive regimens, by including relevant evidence on the comparative effects of RAS blockade. Specifically, the direct comparisons of ACE inhibitors, ARBs, the DR inhibitor aliskiren, and their combinations are largely driven by head-to-head comparative data, including a number of large randomized trials [39–41,55,75]. In 2003, results of the Valsartan in Acute Myocardial Infarction Trial (VALIANT) [39] found no significant differences between an ARB and an ACE inhibitor in all-cause mortality or major cardiovascular events among patients with myocardial infarction complicated by congestive heart failure and/or evidence of left ventricular systolic dysfunction. However, combining an ARB and an ACE inhibitor increased the rate of adverse events without improving survival. Similarly, in 2008, the Ongoing Telmisartan Alone and in Combination with Ramipril Global Endpoint Trial (ONTARGET) [40,41] showed no differences between an ACE inhibitor and an ARB, alone or in combination, for major cardiovascular and renal events, but highlighted the danger of dual blockade of RAS, reporting an increased risk of acute dialysis and hyperkalemia in patients with vascular disease or high-risk diabetes and who were prescribed an ACE inhibitor and an ARB together. To specifically assess the renal effects of dual therapy, the Veterans Affairs Nephropathy in Diabetes (VA NEPHRON-D) trial [75] was conducted in patients with diabetes mellitus and diabetic nephropathy. In line with previous studies [39–41], the VA NEPHRON-D trial showed that dual therapy with an ACE inhibitor and an ARB yielded no significant benefit with respect to the endpoints of renal disease progression, cardiovascular disease, or all-cause mortality, but was associated with an increased risk of adverse events compared with ARB alone.

Our findings reinforce the recommendations of current guidelines in North America and Europe suggesting that ACE inhibitors and ARBs, as preferred antihypertensive therapies, have generally similar effects on cardiovascular and renal outcomes [6–9]. Several points also deserve to be mentioned. Our findings show that dual therapy with an ACE inhibitor and an ARB had no additional beneficial effect on major cardiovascular and renal outcomes when compared to an ACE inhibitor or ARB alone in adults with diabetes. Accordingly, given the lack of consistent benefits on major clinical outcomes and considering the existent evidence [22,30,39–41,75] of adverse effects in terms of hypotension, hyperkalemia, and acute kidney injury, overall recommendations cautioning against the use of combination therapy with an ACE inhibitor and an ARB are still valid. In addition, this review provided relevant evidence to refute the claim raised during the last decade that ARBs may increase the risk of cardiovascular outcomes [76–79]. For example, results of the Randomized Olmesartan and Diabetes Microalbuminuria Prevention (ROADMAP) trial [50] and the Olmesartan Reducing Incidence of End Stage Renal Disease in Diabetic Nephropathy Trial (ORIENT) [80] showed an increased number of cardiovascular deaths among diabetic patients randomized to the single ARB olmesartan. Regarding cardiovascular safety, the US Food and Drug Administration and the European Medicines Agency conducted safety reviews with inconclusive findings [81,82]. Overall, our network meta-analysis found that ARBs as a class were not associated with an increased risk of cardiovascular risk (major cardiovascular outcome, cardiovascular death, myocardial infarction, stroke, angina pectoris, or hospitalization for heart failure) when compared with ACE inhibitors or placebo. Our evaluation showed favorable effects of ARBs as a class compared with placebo for the risk of myocardial infarction, stroke, and hospitalization for heart failure; potential benefits for decreasing the risk of major cardiovascular outcome and angina pectoris; and neutral effects on cardiovascular mortality and all-cause mortality.

For the DR inhibitor aliskiren, our review identified limited evidence and did not allow us to reach definitive conclusions for any of the cardiovascular and renal outcomes of interest. Most trials currently available in the literature comparing therapy with DR inhibitor have focused on surrogate outcomes and have been underpowered to provide robust estimates of major outcomes and adverse events [5,30]. The Aliskiren Trial in Type 2 Diabetes Using Cardiovascular and Renal Disease Endpoints (ALTITUDE) [22] trial was stopped prematurely because the addition of the DR inhibitor aliskiren to standard therapy with RAS blockade did not result in a decrease of cardiovascular and renal events and increased the risk of adverse outcomes such as stroke, hyperkalemia, and hypotension. Similarly, the Aliskiren Trial on Acute Heart Failure Outcomes (ASTRONAUT) [56] failed to show that the DR inhibitor was superior to placebo in addition to standard therapy in reducing cardiovascular death or heart failure rehospitalization among patients hospitalized for heart failure with reduced left ventricular ejection fraction. ASTRONAUT was stopped early, and event rates of hyperkalemia, hypotension, and renal impairment/renal failure were found to be higher in the aliskiren group. In ASTRONAUT, all-cause mortality was significantly increased with aliskiren in patients with diabetes but not in those without diabetes. As a consequence of these findings, regulatory authorities recommended new contraindications and warnings for aliskiren-containing medicines in patients with diabetes or kidney problems [83] and required aliskiren investigational treatment to be withdrawn from patients with diabetes in Aliskiren Trial to Minimize Outcomes in Patients with Heart Failure (ATMOSPHERE) [84,85], an ongoing trial of more than 7,000 patients with heart failure comparing aliskiren and the ACE inhibitor enalapril, alone and in combination.

During the performance and reporting of our review, an additional network meta-analysis of antihypertensive medications in diabetes and kidney disease was published and identified. Palmer et al. [42] evaluated blood-pressure-lowering agents in 157 studies comprising 43,256 participants and concluded that no treatment prolonged survival, but ACE inhibitor and ARB treatment, alone or in combination, were the most effective strategies against ESRD. Compared with placebo, they found that the combination of an ACE inhibitor and an ARB seemed to prevent ESRD, did not increase doubling of serum creatinine, and improved albuminuria, at the expense of an increased risk of hyperkalemia and acute kidney injury. Although our review indicates a fairly consistent pattern of findings with Palmer et al. [42] for ESRD and all-cause mortality for ACE inhibitors and ARBs against placebo, our network meta-analysis provided greater insight into the comparative effects of classes of RAS blockers on major cardiovascular and renal outcomes, largely based on expanded long-term trial datasets (considering both unpublished outcomes and events).

There are limitations to be noted regarding our review. First, as in other meta-analyses, there is clinical and methodological heterogeneity in the included trials in terms of different study characteristics and broad group populations according to the original trial designs. These data should be viewed as reflecting real world practice treatment with different RAS blockers, reflecting more closely the heterogeneous case mix of diabetic patients encountered in clinical practice. Second, we used study-level data instead of individual patient data, so the small number of studies limited the sensitivity analyses that could be conducted to account for heterogeneity in the absence of patient-level data. Although meta-regression analyses were performed to evaluate the effect of potential effect modifiers, the results of these analyses may be underpowered. Given the lack of consistently reported data in each trial, we did not adjust our analyses for treatment dose or adherence to therapy. Third, we followed the clinical guidelines approach of formulating our treatment comparisons at the drug-class level. Caution should be used when interpreting findings, considering the fact that not all single ACE inhibitor/ARB agents are represented in the randomized trials that formed the evidence base for our network meta-analyses. Fourth, our study does not consider costs or patient preferences for the different treatment strategies and outcomes. Future research needs to be conducted to explore the comparative cost-effectiveness of RAS blockade. Fifth, we evaluated major cardiovascular and renal outcomes using composite endpoints for a comparative effectiveness assessment. In past decades, the composite endpoints of major cardiovascular outcome and progression of renal disease have become standard outcomes of large randomized trials [13,40,41,75]. The power of analyses may be increased when such composite measures are used as compared with the individual components of these measures, since by grouping numerous types of events into a larger category, the composite endpoint will occur more frequently than any of the individual components. Confidence in composite endpoints depends partly on a belief that similar risk reductions apply to all the components of the composite endpoint. However, it is acknowledged that the use of composite endpoints is frequently complicated because of poor reporting and uncertain clinical relevance in many trials [86,87]. For example, our results showed no benefits of RAS blockers over placebo in the composite endpoint of progression of renal disease (a composite of the onset of ESRD, doubling of the baseline serum creatinine concentration, or death from any cause). ESRD was the only renal outcome for which RAS blockers (ACE inhibitor or ARB alone or in combination) were convincingly beneficial. Indeed, in this instance, ACE inhibitors reduced the incidence of both ESRD and doubling of creatinine. No RAS blocker reduced overall mortality in adults with diabetes. These examples highlight the challenges that clinicians, trialists, and guideline developers face when making decisions on the basis of composite endpoints, particularly when results for each single component of a composite endpoint are not presented separately in published reports. Although we adopted reproducible definitions that will enhance cross-study comparisons, this review was challenged by the heterogeneous reporting of events in studies. None of the trials examined and reported all of the outcomes of interest. It is also important to emphasize the limited ability of randomized controlled trials with a median duration of about 3 y to inform lifelong treatment decisions. Finally, publication bias was not quantitatively assessed, as there were inadequate numbers of included trials with direct comparisons to properly assess a funnel plot or use more advanced regression-based methods.

### Conclusions

Using randomized trial data and a novel evidence synthesis approach, our analyses indicate that comparisons of different RAS blockers showed similar effects of ACE inhibitors and ARBs on major cardiovascular and renal outcomes in adults with diabetes mellitus. Compared with monotherapy, the combination of an ACE inhibitor and an ARB failed to provide significant benefits in terms of major outcomes. Clinicians should discuss the balance between benefits, costs, and potential harms with the individual patient with diabetes before starting treatment. These findings are important for helping clinicians, health-care providers, policy-makers, and patients with diabetes make informed decisions regarding treatment selection.

## Supporting Information

S1 Checklist PRISMA network meta-analysis checklist of items to include when reporting a systematic review involving a network meta-analysis. (DOCX)

S1 Fig PRISMA flow diagram for study selection process. (DOCX)

S2 Fig Network geometry of all treatment comparisons included in the analyses. (DOCX)

S1 Table Baseline characteristics of included studies. (DOCX)

S2 Table Study-specific outcome-level assessment of the quality of evidence (high, moderate, low, very low, and not reported). (DOCX)

S3 Table Risk of bias and sponsorship of included studies. (DOCX)

S4 Table Summary characteristics of included studies. (DOCX)

S5 Table Reporting of outcomes in included studies. (DOCX)

S6 Table Number of cardiovascular events per trial and treatment comparison. (DOCX)

S7 Table Number of deaths and renal events per trial and treatment comparison. (DOCX)

S8 Table Main results for all possible treatment comparisons. Summary ORs and CrIs.
(DOCX)

S9 Table Summary of SUCRA values with 95% CrIs, by outcome and treatment. (DOCX)

S10 Table Sensitivity analyses. (DOCX)

S11 Table Summary of model fit statistics from network meta-analysis by outcome. (DOCX)

S12 Table Methodological differences from previous reviews of cardiovascular and/or renal outcomes of RAS blockade in patients with diabetes. (DOCX)

S13 Table Randomized controlled trials included in our systematic review versus previous reviews. (DOCX)

S14 Table Randomized controlled trials excluded in our systematic review that were included in previous reviews. (DOCX)

S1 Text PubMed search terms. (DOCX)

S2 Text List of screened systematic reviews and meta-analyses. (DOCX)

S3 Text Example of WinBUGS code for main analyses. (DOCX)

S4 Text List of included clinical trials. (DOCX)

## Abbreviations

ACE: angiotensin-converting enzyme
ARB: angiotensin receptor blocker
BB: beta blocker
CCB: calcium channel blocker
CI: confidence interval
CrI: credible interval
DIC: deviance information criterion
DR: direct renin
ESRD: end-stage renal disease
OR: odds ratio
RAS: renin–angiotensin system
SUCRA: surface under the cumulative ranking curve
