# Correction: Cardiovascular and Renal Outcomes of Renin-Angiotensin System Blockade in Adult Patients with Diabetes Mellitus: A Systematic Review with Network Meta-Analyses

**DOI:** 10.1371/journal.pmed.1002064

**Published:** 2016-06-10

**Authors:** The PLOS Medicine Staff

## Notice of Republication

This article was republished on May 19^th^, 2016 to correct poor figure quality in the PDF version, which was introduced during the typesetting process. The publisher apologizes for these issues with the figure quality. Please download this article again to view the updated version.
