# Supplementary material for: Cardiovascular and Renal Outcomes of Renin–Angiotensin System Blockade in Adult Patients with Diabetes Mellitus: A Systematic Review with Network Meta-Analyses
Source: PLoS Med. 2016 Mar 8;13(3):e1001971. doi: 10.1371/journal.pmed.1001971 (PMC4783064; doi:10.1371/journal.pmed.1001971)
Supplement: S1 Fig — (DOCX) [file pmed.1001971.s002.docx]

**S1 Fig. PRISMA Flow Diagram for Study Selection Process**

## Identification

Records identified through database searching:

PubMed (2004- July 2014): n = 321 Cochrane Database of Systematic Reviews: n = 25

Additional records identified through other sources:

n = 5

## Screening

Records screened after duplicates removed
(n = 343)

Records excluded
n = 311

Records of systematic reviews included: n = 32

## Eligibility

## Included

Additional citations screened/identified by reviewers: n = 11

Citations screened from PubMed ‘related search’:
n = 3604*

Citations screened from included systematic reviews: n = 712

Articles included in both qualitative and quantitative synthesis/meta-analysis: n = 88 (studies included n = 71)

Full-text articles excluded: n = 246

- Non- relevant population and/or comparison: 124
- Follow-up < 1 year: 71
- Non-relevant outcome: 47
- Other: 4

Full-text articles of clinical trials assessed for eligibility:
n = 334

Records excluded
n = 3993

*Related search based on 71 included articles as of 5^th^ September 2014
