# Supplementary material for: Cardiovascular and Renal Outcomes of Renin–Angiotensin System Blockade in Adult Patients with Diabetes Mellitus: A Systematic Review with Network Meta-Analyses
Source: PLoS Med. 2016 Mar 8;13(3):e1001971. doi: 10.1371/journal.pmed.1001971 (PMC4783064; doi:10.1371/journal.pmed.1001971)
Supplement: S2 Fig — (DOCX) [file pmed.1001971.s003.docx]

**S2 Fig.**

**Figure 2a. Network geometry of all treatment comparisons for cardiovascular composite.**

Solid lines represent direct comparisons within randomized controlled trials. The size of nodes is proportional to the number of randomized participants (sample size) and the width of the lines is proportional to the number of trials comparing each pair of treatments. Nodes in green represent RAS blockers (in monotherapy and/or combinations). Nodes in blue represent other control arms were included in the evidence networks to preserve randomization.

**Figure 2b. Network geometry of all treatment comparisons for cardiovascular death.**

Solid lines represent direct comparisons within randomized controlled trials. The size of nodes is proportional to the number of randomized participants (sample size) and the width of the lines is proportional to the number of trials comparing each pair of treatments. Nodes in green represent RAS blockers (in monotherapy and/or combinations). Nodes in blue represent other control arms were included in the evidence networks to preserve randomization.

**Figure 2c. Network geometry of all treatment comparisons for acute myocardial infarction.**

Solid lines represent direct comparisons within randomized controlled trials. The size of nodes is proportional to the number of randomized participants (sample size) and the width of the lines is proportional to the number of trials comparing each pair of treatments. Nodes in green represent RAS blockers (in monotherapy and/or combinations). Nodes in blue represent other control arms were included in the evidence networks to preserve randomization.

**Figure 2d. Network geometry of all treatment comparisons for stroke.**

Solid lines represent direct comparisons within randomized controlled trials. The size of nodes is proportional to the number of randomized participants (sample size) and the width of the lines is proportional to the number of trials comparing each pair of treatments. Nodes in green represent RAS blockers (in monotherapy and/or combinations). Nodes in blue represent other control arms were included in the evidence networks to preserve randomization.

**Figure 2e. Network geometry of all treatment comparisons for progression of renal disease (renal composite endpoint).**

Solid lines represent direct comparisons within randomized controlled trials. The size of nodes is proportional to the number of randomized participants (sample size) and the width of the lines is proportional to the number of trials comparing each pair of treatments. Nodes in green represent RAS blockers (in monotherapy and/or combinations). Nodes in blue represent other control arms were included in the evidence networks to preserve randomization.

**Figure 2f. Network geometry of all treatment comparisons for end-stage renal disease.**

Solid lines represent direct comparisons within randomized controlled trials. The size of nodes is proportional to the number of randomized participants (sample size) and the width of the lines is proportional to the number of trials comparing each pair of treatments. Nodes in green represent RAS blockers (in monotherapy and/or combinations). Nodes in blue represent other control arms were included in the evidence networks to preserve randomization.

**Figure 2g. Network geometry of all treatment comparisons for doubling of serum creatinine.**

Solid lines represent direct comparisons within randomized controlled trials. The size of nodes is proportional to the number of randomized participants (sample size) and the width of the lines is proportional to the number of trials comparing each pair of treatments. Nodes in green represent RAS blockers (in monotherapy and/or combinations). Nodes in blue represent other control arms were included in the evidence networks to preserve randomization.

**Figure 2h. Network geometry of all treatment comparisons for all-cause of death.**

Solid lines represent direct comparisons within randomized controlled trials. The size of nodes is proportional to the number of randomized participants (sample size) and the width of the lines is proportional to the number of trials comparing each pair of treatments. Nodes in green represent RAS blockers (in monotherapy and/or combinations). Nodes in blue represent other control arms were included in the evidence networks to preserve randomization.

**Figure 2i. Network geometry of all treatment comparisons for angina pectoris.**

Solid lines represent direct comparisons within randomized controlled trials. The size of nodes is proportional to the number of randomized participants (sample size) and the width of the lines is proportional to the number of trials comparing each pair of treatments. Nodes in green represent RAS blockers (in monotherapy and/or combinations). Nodes in blue represent other control arms were included in the evidence networks to preserve randomization.

**Figure 2j. Network geometry of all treatment comparisons for heart failure.**

Solid lines represent direct comparisons within randomized controlled trials. The size of nodes is proportional to the number of randomized participants (sample size) and the width of the lines is proportional to the number of trials comparing each pair of treatments. Nodes in green represent RAS blockers (in monotherapy and/or combinations). Nodes in blue represent other control arms were included in the evidence networks to preserve randomization.
