# Supplementary material for: Cardiovascular and Renal Outcomes of Renin–Angiotensin System Blockade in Adult Patients with Diabetes Mellitus: A Systematic Review with Network Meta-Analyses
Source: PLoS Med. 2016 Mar 8;13(3):e1001971. doi: 10.1371/journal.pmed.1001971 (PMC4783064; doi:10.1371/journal.pmed.1001971)
Supplement: S1 Table — (DOCX) [file pmed.1001971.s004.docx]

**S1 Table. Baseline characteristics of included studies**

| **Trial name, year** | **No of diabetic patients** | **Mean age (years)** | **Men %** | **Follow-up (years)** | **Diabetes mellitus** | **Mean diabetes duration (years)** | **Hypertension %** | **Coronary disease %** | **Level albuminuria** | **Treatment comparisons** | **Risk of bias** | **Source of data** |
| --- | --- | --- | --- | --- | --- | --- | --- | --- | --- | --- | --- | --- |
| Parving et al 1989^1,2^ | 32 | 31 | 72% | 8 | Type 1 | 20.0 | Normotension | - | Macro | ACEi (captopril); Placebo | High | Journal article |
| Bauer et al 1992^3^ | 33 | 50 | 73% | 1.5 | Mixed (30.3% type 1) | 17.3 | 67% | - | Macro | ACEi (enalapril); Placebo | Unclear | Journal article |
| Björck et al 1992^4^ | 40 | 42 | - | 2.2 | Type 1 | 25.0 | 95% | - | Macro | ACEi (enalapril); β-blocker (metoprolol) | High | Journal article |
| Chan et al 1992^5^ | 102 | 58 | 40% | 1.0 | Type 2 | 5.5 | Yes | - | Mixed (normo, micro and macro) | ACEi (enalapril); CCB (nifedipine) | Unclear | Journal article |
| Lacourcière et al 1993^6^ | 109 | 57 | 57% | 3.0 | Type 2 | 7.0 | Yes | - | Mixed (normo and micro) | ACEi (captopril); Control | Unclear | Journal article |
| Lewis et al 1993^7^ | 409 | 35 | 53% | 3.0 | Type 1 | 22.0 | 75% | - | Macro | ACEi (captopril); Placebo | Unclear | Journal article |
| Ravid et al 1993^8^ | 108 | 47 | 49% | 5.0 | Type 2 | 13.4 | Normotension | - | Micro | ACEi (enalapril); Placebo | Unclear | Journal article |
| Elving et al 1994^9^ | 30 | 37 | 77% | 2.0 | Type 1 | 26.0 | 37% | - | Macro | ACE inhibitor (captopril); β-blocker (atenolol) | High | Journal article |
| Sano et al 1994^10^ | 52 | 64 | - | 4.0 | Type 2 | 12.0 | 50% | - | Micro | ACEi (enalapril);  Control | High | Journal article |
| Laffel et al 1995^11^ | 143 | 33 | 51% | 2.0 | Type 1 | 18.3 | Normotension | - | Micro | ACEi (captopril); Placebo | Unclear | Journal article |
| Bakris et al 1996^12^ | 52 | 62 | 50% | 6.0 | Type 2 | 13.7 | Yes | - | Macro | ACEi (lisinopril); CCB (diltiazem/verapamil); β-blocker (atenolol) | High | Journal article |
| Viberti et al 1996^13^ | 235 | 32 | 52% | 2.0 | Type 1 | 17.8 | Normotension | - | Micro | ACEi (captopril); Placebo | Unclear | Journal article |
| Nielsen et al 1997^14,15^ | 43 | 61 | 63% | 3.5 | Type 2 | 12.5 | Yes | - | Macro | ACEi (enalapril); β-blocker (atenolol) | Unclear | Journal article |
| ABCD-Hypertension 1998^16,17^ | 470 | 57 | 67% | 5.6 | Type 2 | 8.6 | Yes | 25% | Mixed (micro and macro) | ACEi (enalapril); CCB (nisoldipine) | Low | Journal article |
| ABCD-normo 2002^18^ | 480 | 59 | 54% | 5.0 | Type 2 | 9.0 | Normotension | 25% | Mixed (micro and macro) | ACEi (enalapril); CCB (nisoldipine) | Low | Journal article |
| Crepaldi et al 1998^19^ | 92 | 37 | 68% | 3.0 | Type 1 | 19.0 | Normotension | - | Micro | ACEi (lisinopril); CCB (nifedipine); Placebo | Unclear | Journal article |
| FACET 1998^20^ | 380 | 63 | 60% | 3.5 | Type 2 | 10.6 | Yes | - | Mixed (normo and micro) | ACEi (fosinopril); CCB (amlodipine) | Unclear | Journal article |
| Nankervis et al 1998^21^ | 40 | 46 | 80% | 3.0 | Mixed/ Unspecific | 15.0 | 42% | - | Micro | ACEi (perindopril); Placebo | Unclear | Journal article |
| Ravid et al 1998^22^ | 194 | 55 | 39% | 6.0 | Type 2 | 5.8 | Normotension | - | Normo | ACEi (enalapril); Placebo | Unclear | Journal article |
| UKPDS-39  1998^23^ | 758 | 56 | 54% | 9.0 | Type 2 | 2.7 | Yes | - | Mixed (micro and macro) | ACEi (captopril); β-blocker (atenolol) | Unclear | Journal article |
| Fogari et al 1999^24^ | 107 | 56 | 100% | 2.0 | Type 2 | 8.3 | Yes | - | Macro | ACEi (ramipril); CCB (nitrendipine) | Unclear | Journal article |
| ATLANTIS 2000^25^ | 140 | 40 | 71% | 2.0 | Type 1 | 20.3 | Normotension | - | Micro | ACEi (ramipril); Placebo | Unclear | Journal article |
| Tarnow et al 2000^26^ | 52 | 38 | 61% | 4.0 | Type 1 | 24.5 | Yes | - | Macro | ACEi (lisinopril); CCB (nisoldipine) | Unclear | Journal article |
| Chan et al 2000^27^ | 102 | 58 | - | 5.5 | Type 2 | - | Yes | - | Mixed (normo, micro and macro) | ACEi (enalapril); CCB (nifedipine) | Unclear | Journal article |
| STOP HTN-2 2000^28^ | 719 | 76 | 40% | 4.0 | Type 2 | - | Yes | 14% | - | ACEi (-); CCB (-); Control | Unclear | Journal article |
| Micro-HOPE 2000^29^ | 3577 | 65 | 63% | 4.5 | Type 2 | 11.4 | 56% | 60% | Micro | ACE inhibitor (ramipril); Placebo | Low | Journal article |
| J-MIND 2001^30^ | 436 | 60 | 51% | 2.0 | Type 2 | 9.0 | Yes | - | Mixed (normo and micro) | ACEi (enalapril); CCB (nifedipine) | High | Journal article |
| IDNT 2001^31,32^ | 1715 | 59 | 66% | 2.6 | Type 2 | 14.8 | Yes | 29% | Macro | ARB (irbesartan); CCB (amlodipine); Placebo | Unclear | Journal article and sponsor communication |
| IRMA-2 2001^33^ | 608 | 58 | 68% | 2.0 | Type 2 | 9.9 | Yes | 8% | Micro | ARB (irbesartan); Placebo | Unclear | Journal article and sponsor communication |
| Jerums et al 2001^34^ | 42 | 30 | 40% | 2.0 | Type 1 | 14.5 | Normotensive | - | Micro | ACEi (perindopril); CCB (nifedipine); Placebo | Unclear | Journal article |
| RENAAL 2001^35,36^ | 1513 | 60 | 63% | 3.4 | Type 2 | ≥ 5.0 (90%)* | 94% | 11% | Macro | ARB (losartan); Placebo | Low | Journal article and FDA dockets |
| CAPPP 2001^37^ | 572 | 55 | 62% | 6.1 | Type 2 | - | Yes | 10% | Mixed (normo and micro) | ACEi (captopril);  Control | Unclear | Journal article |
| Val-HeFT 2001^38^ | 1185 | 62 | 80% | 1.9 | Mixed/ Unspecific | - | Normotensive | 57% | - | ACEi + ARB (ACEi + valsartan); ACEi (-) | Low | Journal article and sponsor communication |
| Fogari et al 2002^39^ | 309 | 63 | 57% | 4.0 | Type 2 | 8.8 | Yes | - | Micro | ACEi (fosinopril); CCB (amlodipine); ACEi (fosinopril) + CCB (amlodipine) | High | Journal article |
| JAPAN-IDDM 2002^40^ | 79 | 33 | 35% | 1.5 | Type 1 | 14.4 | 18% | - | Macro | ACEi (captopril);  Placebo | Unclear | Journal article |
| LIFE 2002^41^ | 1195 | 67 | 47% | 4.7 | Type 2 | - | Yes | 24% | Mixed (normo, micro and macro) | ARB (losartan); β-blocker (atenolol) | Low | Journal article |
| VALIANT 2003^42^ | 3400 | 65 | 69% | 2.0 | Type 2 | - | 55% | Yes | - | ACEi (captopril); ARB (valsartan); ACEi + ARB (captopril + valsartan) | Low | Journal article and sponsor communication |
| VALUE 2004^43^ | 4823 | 67 | 57% | 4.2 | Mixed/ Unspecific | - | Yes | 46% | Mixed | ARB (valsartan); CCB (amlodipine) | Low | Journal article and sponsor communication |
| BENEDICT 2004^44^ | 1204 | 62 | 53% | 3.6 | Type 2 | 7.8 | Yes | - | Normo | ACEi (trandolapril); CCB (verapamil); ACEi (trandolapril) + CCB (verapamil); Placebo | Unclear | Journal article |
| DETAIL 2004^45,46^ | 250 | 61 | 73% | 5.0 | Type 2 | 8.0 | Yes | 49% | Mixed (micro and macro) | ACEi (enalapril);  ARB (telmisartan) | Low | Journal article |
| DIABHYCAR 2004^47^ | 4912 | 65 | 70% | 3.9 | Type 2 | 9.8 | 56% | 6% | Mixed (micro and macro) | ACEi (ramipril);  Placebo | Low | Journal article |
| NESTOR 2004^48^ | 570 | 60 | 66% | 1.0 | Type 2 | 8.2 | Yes | - | Micro | ACEi (enalapril);  Diuretic (indapamide) | Unclear | Journal article |
| JMIC-B 2004^49^ | 372 | 63 | 69% | 3.0 | Type 2 | - | Yes | 59% | - | ACEi (-); CCB (nifedipine) | Unclear | Journal article |
| Ko et al 2005^50^ | 42 | 61 | 40% | 1.0 | Type 2 | 9.6 | Yes | - | Mixed (normo, micro and macro) | ACEi (enalapril);  ARB (valsartan) | High | Journal article |
| Schram et al 2005^51^ | 70 | 62 | 61% | 1.0 | Type 2 | - | Yes | - | Mixed (normo and micro) | ACEi (lisinopril);  ARB (candesartan); Diuretic (hydrochlorothiazide) | Unclear | Journal article |
| PERSUADE 2005^52^ | 1502 | 62 | 82% | 4.3 | Mixed/ Unspecific | - | 39% | 67% | - | ACEi (perindopril);  Placebo | Low | Journal article and sponsor communication |
| ALLHAT 2005^53,54^ | 13168 | 67 | 51% | 4.9 | Mixed/ Unspecific | - | Yes | 20% | Mixed (-) | ACEi (lisinopril); CCB (amlodipine); Diuretic (chlorthalidone) | Low | Journal article and investigators communication |
| SCOPE 2005^55^ | 599 | 76 | 35% | 3.7 | Type 2 | - | Yes | - | - | ARB (candesartan); Control | Unclear | Journal article |
| ABCD-2V 2006^56^ | 129 | 56 | 67% |  | Type 2 | 7.3 | Normotensive | 5% | Mixed (normo and micro) | ARB (valsartan); Control | High | Journal article |
| Tong et al 2006^57^ | 38 | 66 | 65% | 2.0 | Type 2 | - | Yes | - | Mixed (micro and macro) | ACEi (fosinopril);  Placebo | Unclear | Journal article |
| ADVANCE 2007^58,59^ | 11140 | 66 | 57% | 4.3 | Type 2 | - | 69% | 12% | Mixed (normo, micro and macro) | ACEi + Diuretic (perindopril + indapamide);  Placebo | Low | Journal article |
| DIRECT-Prevent 1 2008^60,61^ | 1421 | 30 | 57% | 4.7 | Type 1 | 6.7 | Normotensive | - | Normo | ARB (candesartan); Placebo | Low | Journal article and sponsor communication |
| DIRECT-Protect 1 2008^60,61^ | 1905 | 31 | 57% | 4.8 | Type 1 | 11.0 | Normotensive | - | Normo | ARB (candesartan); Placebo | Low | Journal article and sponsor communication |
| DIRECT-Protect 2 2008^60,62,63^ | 1905 | 57 | 50% | 4.7 | Type 2 | 8.7 | 62% | 5% | Normo | ARB (candesartan); Placebo | Low | Journal article and sponsor communication |
| GUARD 2008^64^ | 332 | 58 | 65% | 1.0 | Type 2 | - | Yes | - | Mixed (micro and macro) | ACEi + CCB (benazepril + amlodipine);  ACEi + Diuretic (benazepril + hydrochlorothiazide) | Unclear | Journal article |
| PRoFESS 2008^65^ | 5743 | 66 | 65% | 2.5 | Type 2 | - | 74% | 19% | - | ARB (telmisartan); Placebo | Low | Journal article and sponsor communication |
| ONTARGET 2008^66-68^ | 9612 | 66 | 68% | 4.7 | Mixed/ Unspecific | - | 77% | 64% | Mixed (normo, micro and macro) | ACEi (ramipril); ARB (telmisartan); ACEi + ARB (ramipril + telmisartan) | Low | Journal article and sponsor communication |
| TRANSCEND 2008^69-71^ | 2118 | 67 | 57% | 4.7 | Mixed/ Unspecific | - | 76% | 75% | Mixed (normo, micro and macro) | ARB (telmisartan); Placebo | Low | Journal article and sponsor communication |
| Kohlmann Jr et al 2009^72^ | 110 | 63 | 56% | 1.0 | Type 2 | 6.5 | Yes | - | Micro | ACEi + CCB (delapril + manidipine);  ARB + Diuretic (losartan + hydrochlorothiazide) | Unclear | Journal article |
| Mehdi et al 2009^73^ | 80 | 51 | 47% | 1.0 | Mixed | 16.1 | Yes | - | Macro | ACEi + ARB (lisinopril + losartan); ACEi + Diuretic (lisinopril + spironolactone); ACEi (lisinopril) | Unclear | Journal article |
| RAAS 2009^74^ | 285 | 29 | 46% | 5.0 | Type 1 | 11.2 | Normotensive | - | Normo | ACEi (enalapril);  ARB (losartan); Placebo | Unclear | Journal article |
| CASE-J 2010^75^ | 2018 | 63 | 56% | 3.2 | Type 2 | - | Yes | 30% | Mixed | ARB (candesartan); CCB (amlodipine) | Unclear | Journal article and investigators communication |
| ROADMAP 2011^76^ | 4447 | 58 | 46% | 3.2 | Type 2 | 6.1 | 94% | 25% | Normo | ARB (olmesartan); Placebo | Low | Journal article and sponsor communication |
| ORIENT 2011^77,78^ | 563 | 59 | 68% | 3.2 | Type 2 | - | Yes | 16% | Macro | ACEi (-); ARB (olmesartan); ACEi + ARB (ACEi + olmesartan); Placebo | Low | Journal article |
| DEMAND 2011^79^ | 380 | 61 | 65% | 3.0 | Type 2 | 6.3 | Yes | - | Mixed (normo and micro) | ACEi + CCB (delapril + manidipine); ACEi (delaparil); Placebo | Low | Journal article |
| ALTITUDE 2012^80^ | 8579 | 64 | 68% | 2.7 | Type 2 | > 5.0 (82%)* | 94% | 42% | Mixed (micro and macro) | DRi + ACEi (aliskiren + ACEi); DRi + ARB (aliskiren + ARB); ACEi; ARB | Low | Journal article and sponsor communication |
| NAGOYA HEART 2012^81,82^ | 1150 | 63 | 66% | 3.2 | Type 2 | - | Yes | 27% | - | ARB (valsartan); CCB (amlodipine) | Unclear | Journal article |
| VA NEPHRON-D  2013^83^ | 1448 | 65 | 99% | 2.2 | Type 2 | - | Yes | 23% | Macro | ARB (losartan); ACEi + ARB (lisinopril + losartan) | Low | Journal article |
| ASTRONAUT 2013^84,85^ | 662 | 66 | 77% | 1.0 | Mixed/ Unspecific |  | 82% | 73% | - | DRi + Diuretic (aliskiren + diuretic); Diuretic (-)** | Low | Journal article and sponsor communication |
| COLM 2014^86^ | 1362 | 74 | 51% | 3.3 | Type 2 | - | Yes | - | - | ARB + CCB (-); ARB + Diuretic (-) | Unclear | Journal article and investigators communication |
| OSCAR 2014^87,88^ | 628 | 73 | 48% | 3.0 | Type 2 | - | Yes | 69% | - | ARB + CCB (-); ARB (-) | Unclear | Journal article and investigators communication |
