# Supplementary material for: Cardiovascular and Renal Outcomes of Renin–Angiotensin System Blockade in Adult Patients with Diabetes Mellitus: A Systematic Review with Network Meta-Analyses
Source: PLoS Med. 2016 Mar 8;13(3):e1001971. doi: 10.1371/journal.pmed.1001971 (PMC4783064; doi:10.1371/journal.pmed.1001971)
Supplement: S2 Table — (DOCX) [file pmed.1001971.s005.docx]

**S2 Table. Study specific outcome level assessment (high, moderate, low, very low, and not reported).**

| **Trial name, year** | **Cardiovascular composite** | **Cardiovascular death** | **Myocardial infarction** | **Stroke** | **Heart failure** | **Angina** | **Renal composite** | **Doubling serum creatinine** | **End-stage renal disease** | **Death** |
| --- | --- | --- | --- | --- | --- | --- | --- | --- | --- | --- |
| Parving et al 1989^1,2^ | Not reported | Not reported | Not reported | Not reported | Not reported | Not reported | Not reported | Very low | Very low | Very low |
| Bauer et al 1992^3^ | Not reported | Not reported | Very low | Very low | Not reported | Not reported | Not reported | Not reported | Very low | Very low |
| Björck et al 1992^4^ | Not reported | Not reported | Very low | Not reported | Not reported | Not reported | Not reported | Not reported | Very low | Not reported |
| Chan et al 1992^5^ | Not reported | Very low | Very low | Not reported | Not reported | Very low | Not reported | Not reported | Not reported | Very low |
| Lacourcière et al 1993^6^ | Not reported | Very low | Very low | Not reported | Not reported | Very low | Not reported | Not reported | Not reported | Very low |
| Lewis et al 1993^7^ | Not reported | Not reported | Not reported | Not reported | Not reported | Not reported | Not reported | Moderate | Moderate | Moderate |
| Ravid et al 1993^8^ | Not reported | Not reported | Not reported | Not reported | Not reported | Not reported | Not reported | Low | Not reported | Not reported |
| Elving et al 1994^9^ | Not reported | Not reported | Very low | Not reported | Not reported | Not reported | Not reported | Not reported | Not reported | Not reported |
| Sano et al 1994^10^ | Not reported | Not reported | Not reported | Not reported | Not reported | Not reported | Not reported | Not reported | Not reported | Very low |
| Laffel et al 1995^11^ | Not reported | Not reported | Not reported | Not reported | Not reported | Not reported | Not reported | Not reported | Not reported | Very low |
| Bakris et al 1996^12^ | Not reported | Very low | Not reported | Not reported | Not reported | Not reported | Very low* | Very low | Very low | Very low |
| Viberti et al 1996^13^ | Not reported | Not reported | Not reported | Not reported | Not reported | Not reported | Not reported | Not reported | Not reported | Very low |
| Nielsen et al 1997^14,15^ | Not reported | Very low | Very low | Not reported | Not reported | Not reported | Not reported | Not reported | Very low | Very low |
| ABCD-Hypertension 1998^16,17^ | Not reported | Moderate | Moderate | Moderate | Moderate | Not reported | Not reported | Not reported | Not reported | Moderate |
| ABCD-normo 2002^18^ | Not reported | Moderate | Moderate | Moderate | Moderate | Not reported | Not reported | Not reported | Not reported | Moderate |
| Crepaldi et al 1998^19^ | Not reported | Not reported | Very low | Not reported | Not reported | Not reported | Not reported | Not reported | Not reported | Not reported |
| FACET 1998^20^ | Low* | Not reported | Moderate | Moderate | Not reported | Moderate | Not reported | Not reported | Not reported | Moderate |
| Nankervis et al 1998^21^ | Not reported | Not reported | Not reported | Not reported | Not reported | Not reported | Not reported | Not reported | Not reported | Very low |
| Ravid et al 1998^22^ | Not reported | Not reported | Not reported | Not reported | Not reported | Not reported | Not reported | Low | Not reported | Low |
| UKPDS-39  1998^23^ | Moderate | Moderate | Moderate | Moderate | Moderate | Moderate | Not reported | Not reported | Not reported | Moderate |
| Fogari et al 1999^24^ | Not reported | Not reported | Not reported | Not reported | Not reported | Not reported | Not reported | Not reported | Low | Not reported |
| ATLANTIS 2000^25^ | Not reported | Not reported | Low | Not reported | Not reported | Low | Not reported | Not reported | Not reported | Low |
| Tarnow et al 2000^26^ | Low* | Low | Low | Low | Low | Not reported | Not reported | Low | Not reported | Low |
| Chan et al 2000^27^ | Not reported | Low | Not reported | Not reported | Not reported | Not reported | Not reported | Not reported | Not reported | Low |
| STOP HTN-2 2000^28^ | Moderate | Moderate | Moderate | Moderate | Moderate | Not reported | Not reported | Not reported | Not reported | Moderate |
| Micro-HOPE 2000^29^ | High | High | High | High | High | Moderate | Not reported | Not reported | Moderate | High |
| J-MIND 2001^30^ | Not reported | Not reported | Low | Low | Low | Not reported | Not reported | Not reported | Not reported | Not reported |
| IDNT 2001^31,32^ | Moderate* | High | High | High | High | Moderate | High | High | High | High |
| IRMA-2 2001^33^ | Moderate | Moderate | Moderate | Moderate | Moderate | Moderate | Not reported | Not reported | Not reported | Moderate |
| Jerums et al 2001^34^ | Not reported | Not reported | Not reported | Not reported | Not reported | Not reported | Not reported | Not reported | Not reported | Low |
| RENAAL 2001^35,36^ | Moderate* | High | High | High | High | High | High | High | High | High |
| CAPPP 2001^37^ | Moderate | Moderate | Moderate | Moderate | Moderate | Not reported | Not reported | Not reported | Not reported | Moderate |
| Val-HeFT 2001^38^ | Not reported | Not reported | Not reported | Not reported | Moderate | Moderate | Not reported | Not reported | Not reported | High |
| Fogari et al 2002^39^ | Low | Low | Low | Low | Not reported | Not reported | Not reported | Not reported | Not reported | Low |
| JAPAN-IDDM 2002^40^ | Not reported | Not reported | Not reported | Not reported | Not reported | Not reported | Not reported | Very low | Not reported | Not reported |
| LIFE 2002^41^ | High | High | High | High | High | High | Not reported | Not reported | Not reported | High |
| VALIANT 2003^42^ | High* | High | High | High | High | Moderate | Not reported | Not reported | Not reported | High |
| VALUE 2004^43^ | High | High | High | High | High | High | Not reported | Not reported | High | High |
| BENEDICT 2004^44^ | Not reported | Moderate | Not reported | Not reported | Not reported | Not reported | Not reported | Not reported | Not reported | Moderate |
| DETAIL 2004^45,46^ | Low* | Low | Low | Low | Low | Not reported | Not reported | Not reported | Not reported | Low |
| DIABHYCAR 2004^47^ | Moderate* | High | High | High | High | Moderate | Moderate* | High | High | High |
| NESTOR 2004^48^ | Low* | Low | Low | Low | Not reported | Low | Not reported | Not reported | Not reported | Low |
| JMIC-B 2004^49^ | Low* | Low | Low | Low | Low | Low | Not reported | Not reported | Not reported | Low |
| Ko et al 2005^50^ | Not reported | Not reported | Not reported | Very low | Not reported | Not reported | Not reported | Not reported | Not reported | Not reported |
| Schram et al 2005^51^ | Not reported | Not reported | Very low | Not reported | Not reported | Not reported | Not reported | Not reported | Not reported | Not reported |
| PERSUADE 2005^52^ | High | High | High | High | High | Not reported | Moderate | High | Not reported | High |
| ALLHAT 2005^53,54^ | High | High | High | High | High | High | High | High | High | High |
| SCOPE 2005^55^ | Moderate | Not reported | Not reported | Moderate | Not reported | Not reported | Not reported | Not reported | Not reported | Not reported |
| ABCD-2V 2006^56^ | Not reported | Not reported | Not reported | Low | Not reported | Not reported | Not reported | Not reported | Not reported | Low |
| Tong et al 2006^57^ | Not reported | Not reported | Not reported | Not reported | Not reported | Not reported | Reported | Very low | Not reported | Not reported |
| ADVANCE 2007^58,59^ | High | High | Not reported | High | Not reported | Not reported | Moderate* | High | High | High |
| DIRECT-Prevent 1 2008^60,61^ | Moderate | Moderate | Moderate | Moderate | Moderate | Moderate | Moderate | Moderate | Not reported | Moderate |
| DIRECT-Protect 1 2008^60,61^ | Moderate | Moderate | Moderate | Moderate | Not reported | Moderate | Moderate | Moderate | Not reported | Moderate |
| DIRECT-Protect 2 2008^60,62,63^ | Moderate | Moderate | Moderate | Moderate | Moderate | Moderate | Moderate | Moderate | Not reported | Moderate |
| GUARD 2008^64^ | Not reported | Not reported | Not reported | Not reported | Not reported | Not reported | Not reported | Not reported | Not reported | Low |
| PRoFESS 2008^65^ | High | High | High | High | Not reported | Not reported | Not reported | Not reported | Not reported | High |
| ONTARGET 2008^66-68^ | High | High | High | High | High | High | High | High | High | High |
| TRANSCEND 2008^69-71^ | High | High | High | High | High | High | High | High | High | High |
| Kohlmann Jr et al 2009^72^ | Not reported | Not reported | Very low | Not reported | Not reported | Not reported | Not reported | Not reported | Not reported | Not reported |
| Mehdi et al 2009^73^ | Not reported | Not reported | Very low | Very low | Very low | Not reported | Not reported | Not reported | Not reported | Very low |
| RAAS 2009^74^ | Not reported | Not reported | Not reported | Not reported | Not reported | Not reported | Not reported | Not reported | Not reported | Low |
| CASE-J 2010^75^ | Moderate | Moderate | Moderate | Moderate | Not reported | Moderate | Moderate | Moderate | Moderate | Moderate |
| ROADMAP 2011^76^ | Moderate* | High | High | High | Moderate | High | Moderate* | High | High (no cases) | High |
| ORIENT 2011^77,78^ | Moderate | Moderate | Moderate | Moderate | Not reported | Moderate | Moderate | Moderate | Moderate | Moderate |
| DEMAND 2011^79^ | Not reported | Moderate | Not reported | Not reported | Not reported | Not reported | Not reported | Not reported | Not reported | Moderate |
| ALTITUDE 2012^80^ | High | High | High | High | High | Not reported | High | High | High | High |
| NAGOYA HEART 2012^81,82^ | Not reported | Not reported | Moderate | Moderate | Moderate | Not reported | Not reported | Not reported | Not reported | Moderate |
| VA NEPHRON-D  2013^83^ | Not reported | Not reported | High | High | High | Not reported | Moderate | Not reported | Moderate | High |
| ASTRONAUT 2013^84,85^ | Moderate | Moderate | Moderate | Moderate | Moderate | Moderate | Not reported | Moderate | Not reported | Moderate |
| COLM 2014^86^ | Moderate | Moderate | Moderate | Moderate | Moderate | Moderate | Moderate | Moderate | Moderate | Moderate |
| OSCAR 2014^87,88^ | Not reported | Moderate | Moderate | Moderate | Low | Low | Not reported | Not reported | Not reported | Modertate |
