# Supplementary material for: Cardiovascular and Renal Outcomes of Renin–Angiotensin System Blockade in Adult Patients with Diabetes Mellitus: A Systematic Review with Network Meta-Analyses
Source: PLoS Med. 2016 Mar 8;13(3):e1001971. doi: 10.1371/journal.pmed.1001971 (PMC4783064; doi:10.1371/journal.pmed.1001971)
Supplement: S3 Table — (DOCX) [file pmed.1001971.s006.docx]

**S3 Table. Risk of bias and sponsorship of included studies.**

| **Trial name, year** | **Adequate sequence generation** | **Allocation concealment** | **Blinding** | **Incomplete outcome data addressed** | **Selective outcome reporting** | **Other bias** | **Overall risk of bias** | **Funding source** |
| --- | --- | --- | --- | --- | --- | --- | --- | --- |
| Parving et al 1989^1,2^ | Unclear risk | Unclear risk | High risk | Unclear risk | Low risk | Unclear risk | High risk | Not reported |
| Bauer et al 1992^3^ | Unclear risk | Unclear risk | Low risk | Low risk | Low risk | Unclear risk | Unclear risk | Industry and nonindustry |
| Björck et al 1992^4^ | Unclear risk | Unclear risk | High risk | Low risk | Unclear risk | Low risk | High risk | Industry and nonindustry |
| Chan et al 1992^5^ | Unclear risk | Unclear risk | Low risk | Low risk | Low risk | Low risk | Unclear risk | Industry |
| Lacourcière et al 1993^6^ | Unclear risk | Unclear risk | Unclear risk | Unclear risk | Low risk | Low risk | Unclear risk | Not reported |
| Lewis et al 1993^7^ | Unclear risk | Unclear risk | Low risk | Low risk | Unclear risk | Low risk | Unclear risk | Industry and nonindustry |
| Ravid et al 1993^8^ | Unclear risk | Unclear risk | Low risk | Low risk | Low risk | Low risk | Unclear risk | Nonindustry |
| Elving et al 1994^9^ | Unclear risk | Unclear risk | High risk | Low risk | Low risk | Low risk | High risk | Nonindustry |
| Sano et al 1994^10^ | Unclear risk | Unclear risk | High risk | Low risk | Low risk | Low risk | High risk | Not reported |
| Laffel et al 1995^11^ | Unclear risk | Unclear risk | Low risk | Low risk | Low risk | Low risk | Unclear risk | Industry |
| Bakris et al 1996^12^ | Unclear risk | Unclear risk | High risk | Low risk | Low risk | Low risk | High risk | Nonindustry |
| Viberti et al 1996^13^ | Unclear risk | Unclear risk | Low risk | Low risk | Low risk | Low risk | Unclear risk | Industry |
| Nielsen et al 1997^14,15^ | Unclear risk | Unclear risk | Unclear risk | Low risk | Low risk | Low risk | Unclear risk | Not reported |
| ABCD-Hypertension 1998^16,17^ | Low risk | Low risk | Low risk | Low risk | Low risk | Low risk | Low risk | Industry and nonindustry |
| ABCD-normo 2002^18^ | Low risk | Low risk | Low risk | Low risk | Low risk | Low risk | Low risk | Industry and nonindustry |
| Crepaldi et al 1998^19^ | Unclear risk | Unclear risk | Low risk | Low risk | Low risk | Low risk | Unclear risk | Not reported |
| FACET 1998^20^ | Low risk | Low risk | Unclear risk | Low risk | Low risk | Low risk | Unclear risk | Industry |
| Nankervis et al 1998^21^ | Unclear risk | Unclear risk | Low risk | Low risk | Low risk | Low risk | Unclear risk | Not reported |
| Ravid et al 1998^22^ | Low risk | Low risk | Low risk | Low risk | Unclear | Low risk | Unclear risk | Nonindustry |
| UKPDS-39  1998^23^ | Unclear risk | Unclear risk | Unclear risk | Low risk | Low risk | Low risk | Unclear risk | Industry and nonindustry |
| Fogari et al 1999^24^ | Unclear risk | Unclear risk | Unclear risk | Unclear risk | Unclear risk | Unclear risk | Unclear risk | Not reported |
| ATLANTIS 2000^25^ | Low risk | Low risk | Unclear risk | Unclear risk | Low risk | Low risk | Unclear risk | Industry |
| Tarnow et al 2000^26^ | Unclear risk | Low risk | Unclear risk | Low risk | Low risk | Low risk | Unclear risk | Industry |
| Chan et al 2000^27^ | Unclear risk | Unclear risk | Unclear risk | Low risk | Low risk | Low risk | Unclear risk | Not reported |
| STOP HTN-2 2000^28^ | Unclear risk | Unclear risk | Low risk | Low risk | Low risk | Low risk | Unclear risk | Industry |
| Micro-HOPE 2000^29^ | Low risk | Low risk | Low risk | Low risk | Low risk | Low risk | Low risk | Industry and nonindustry |
| J-MIND 2001^30^ | Low risk | Low risk | High risk | Unclear risk | Unclear risk | Unclear risk | High risk | Not reported |
| IDNT 2001^31,32^ | Unclear risk | Unclear risk | Low risk | Low risk | Low risk | Unclear risk | Unclear risk | Industry |
| IRMA-2 2001^33^ | Unclear risk | Unclear risk | Low risk | Low risk | Low risk | Unclear risk | Unclear risk | Industry |
| Jerums et al 2001^34^ | Unclear risk | Low risk | Low risk | Low risk | Unclear | Unclear | Unclear risk | Industry and nonindustry |
| RENAAL 2001^35,36^ | Low risk | Low risk | Low risk | Low risk | Low risk | Low risk | Low risk | Industry |
| CAPPP 2001^37^ | Unclear risk | Unclear risk | Unclear risk | Low risk | Low risk | Low risk | Unclear risk | Industry |
| Val-HeFT 2001^38^ | Low risk | Low risk | Low risk | Low risk | Low risk | Low risk | Low risk | Industry |
| Fogari et al 2002^39^ | Low risk | Low risk | High risk | Low risk | Low risk | Low risk | High risk | Not reported |
| JAPAN-IDDM 2002^40^ | Unclear risk | Low risk | Low risk | Low risk | Low risk | Low risk | Unclear risk | Nonindustry |
| LIFE 2002^41^ | Low risk | Low risk | Low risk | Low risk | Low risk | Low risk | Low risk | Industry |
| VALIANT 2003^42^ | Low risk | Low risk | Low risk | Low risk | Low risk | Low risk | Low risk | Industry |
| VALUE 2004^43^ | Low risk | Low risk | Low risk | Low risk | Low risk | Low risk | Low risk | Industry |
| BENEDICT 2004^44^ | Unclear risk | Unclear risk | Low risk | Low risk | Low risk | Low risk | Unclear risk | Industry and nonindustry |
| DETAIL 2004^45,46^ | Low risk | Low risk | Low risk | Low risk | Low risk | Low risk | Low risk | Industry |
| DIABHYCAR 2004^47^ | Low risk | Low risk | Low risk | Low risk | Low risk | Low risk | Low risk | Industry and nonindustry |
| NESTOR 2004^48^ | Unclear risk | Unclear risk | Low risk | Low risk | Low risk | Low risk | Unclear risk | Industry |
| JMIC-B 2004^49^ | Unclear risk | Unclear risk | Unclear risk | Low risk | Low risk | Low risk | Unclear risk | Nonindustry |
| Ko et al 2005^50^ | Unclear risk | Unclear risk | High risk | Unclear risk | Unclear risk | Unclear risk | High risk | Not reported |
| Schram et al 2005^51^ | Unclear risk | Unclear risk | Unclear risk | Low risk | Low risk | Low risk | Unclear risk | Industry |
| PERSUADE 2005^52^ | Low risk | Low risk | Low risk | Low risk | Low risk | Low risk | Low risk | Industry |
| ALLHAT 2005^53,54^ | Low risk | Low risk | Low risk | Low risk | Low risk | Low risk | Low risk | Industry and nonindustry |
| SCOPE 2005^55^ | Low risk | Low risk | Unclear risk | Low risk | Low risk | Low risk | Unclear risk | Industry |
| ABCD-2V 2006^56^ | Low risk | Low risk | High risk | Low risk | Low risk | Low risk | High risk | Industry |
| Tong et al 2006^57^ | Unclear risk | Unclear risk | Low risk | Low risk | Low risk | Low risk | Unclear risk | Industry |
| ADVANCE 2007^58,59^ | Low risk | Low risk | Low risk | Low risk | Low risk | Low risk | Low risk | Industry and nonindustry |
| DIRECT-Prevent 1 2008^60,61^ | Low risk | Low risk | Low risk | Low risk | Low risk | Low risk | Low risk | Industry |
| DIRECT-Protect 1 2008^60,61^ | Low risk | Low risk | Low risk | Low risk | Low risk | Low risk | Low risk | Industry |
| DIRECT-Protect 2 2008^60,62,63^ | Low risk | Low risk | Low risk | Low risk | Low risk | Low risk | Low risk | Industry |
| GUARD 2008^64^ | Unclear risk | Unclear risk | Unclear risk | Unclear risk | Unclear risk | Unclear risk | Unclear risk | Industry |
| PRoFESS 2008^65^ | Low risk | Low risk | Low risk | Low risk | Low risk | Low risk | Low risk | Industry |
| ONTARGET 2008^66-68^ | Low risk | Low risk | Low risk | Low risk | Low risk | Low risk | Low risk | Industry and nonindustry |
| TRANSCEND 2008^69-71^ | Low risk | Low risk | Low risk | Low risk | Low risk | Low risk | Low risk | Industry |
| Kohlmann Jr et al 2009^72^ | Unclear risk | Low risk | Low risk | Low risk | Low risk | Low risk | Unclear risk | Industry |
| Mehdi et al 2009^73^ | Unclear risk | Unclear risk | Unclear risk | Low risk | Low risk | Low risk | Unclear risk | Nonindustry |
| RAAS 2009^74^ | Low risk | Unclear risk | Low risk | Low risk | Low risk | Low risk | Unclear risk | Industry and nonindustry |
| CASE-J 2010^75^ | Unclear risk | Unclear risk | Unclear risk | Low risk | Low risk | Low risk | Unclear risk | Industry and nonindustry |
| ROADMAP 2011^76^ | Low risk | Low risk | Low risk | Low risk | Low risk | Low risk | Low risk | Industry |
| ORIENT 2011^77,78^ | Low risk | Low risk | Low risk | Low risk | Low risk | Low risk | Low risk | Industry |
| DEMAND 2011^79^ | Low risk | Low risk | Low risk | Low risk | Low risk | Low risk | Low risk | Industry and nonindustry |
| ALTITUDE 2012^80^ | Low risk | Low risk | Low risk | Low risk | Low risk | Low risk | Low risk | Industry |
| NAGOYA HEART 2012^81,82^ | Low risk | Low risk | Unclear risk | Low risk | Low risk | Low risk | Unclear risk | Industry and nonindustry |
| VA NEPHRON-D  2013^83^ | Low risk | Low risk | Low risk | Low risk | Low risk | Low risk | Low risk | Nonindustry |
| ASTRONAUT 2013^84,85^ | Low risk | Low risk | Low risk | Low risk | Low risk | Low risk | Low risk | Industry |
| COLM 2014^86^ | Low risk | Low risk | Unclear risk | Low risk | Low risk | Low risk | Unclear risk | Nonindustry |
| OSCAR 2014^87,88^ | Unclear risk | Unclear risk | Unclear risk | Low risk | Low risk | Low risk | Unclear risk | Nonindustry |

*Estimated by authors based on single components with available information. GRADE Working Group grades of evidence:

- High quality: further research is very unlikely to change our confidence in the estimate of effect
- Moderate: further research may change the estimate (and is likely to have an important influence on our confidence in the estimate of effect)
- Low: further research is likely to change the estimate (an is very likely to have an important influence on our confidence in the estimate of effect)
- Very low: any estimate of effect is very uncertain
