# Supplementary material for: Cardiovascular and Renal Outcomes of Renin–Angiotensin System Blockade in Adult Patients with Diabetes Mellitus: A Systematic Review with Network Meta-Analyses
Source: PLoS Med. 2016 Mar 8;13(3):e1001971. doi: 10.1371/journal.pmed.1001971 (PMC4783064; doi:10.1371/journal.pmed.1001971)
Supplement: S4 Table — (DOCX) [file pmed.1001971.s007.docx]

**S4 Table. Summary characteristics of included studies.**

| **Characteristic** | **Category** | **No. of studies (patients)** |
| --- | --- | --- |
| **Diabetic population** | Overall | 71 (103,120) |
|  | Type 2 diabetes mellitus | 47 (64,992) |
|  | Type 1 diabetes mellitus | 14 (4,905) |
|  | Mixed | 10 (33,223) |
| **Year of primary publication** | 2010-present | 10 (21,237) |
|  | 2000-2009 | 41 (78,454) |
|  | 1990-1999 | 19 (3,397) |
|  | 1980-1989 | 1 (32) |
| **Sponsorship** | Industry/non-industry/partial or mixed/not reported | 33/10/17/11 |
| **Risk of bias** | High/unclear/low | 9/37/25 |
| **Sample size** | Median (range) | 436 (30 – 13,168) |
| **Duration of follow-up (years)** | Median (range) | 3.2 (1.0 – 9.0) |
| **Mean subject age (years)** | Median (range) | 60 (29 – 76) |
| **Mean diabetes duration (years)** | Median (range) | 10.3 (2.7 – 26.0) |
| **% male subjects** | Median (range) | 60.5 (35.0 – 100.0) |
| **Level of albuminuria** | Microalbuminuria | 13 (6,026) |
|  | Macroalbuminuria | 15 (6,196) |
|  | Normoalbuminuria | 7 (11,361) |
|  | Mixed | 25 (62,215) |
|  | Unclear/not reported | 11 (17,322) |
| **Hypertension** | No | 15 (6,443) |
|  | Yes | 37 (36,917) |
|  | Mixed (e.g. 18-95%) | 19 (59,760) |
| **Coronary disease** | Mixed (e.g. 5-75%) | 27 (84,751) |
|  | Yes | 1 (3,400) |
|  | Unclear/not reported | 41 (12,646) |
