# Supplementary material for: Cardiovascular and Renal Outcomes of Renin–Angiotensin System Blockade in Adult Patients with Diabetes Mellitus: A Systematic Review with Network Meta-Analyses
Source: PLoS Med. 2016 Mar 8;13(3):e1001971. doi: 10.1371/journal.pmed.1001971 (PMC4783064; doi:10.1371/journal.pmed.1001971)
Supplement: S5 Table — (DOCX) [file pmed.1001971.s008.docx]

**S5 Table. Reporting of outcomes in included studies.**

| **Outcome** | **No. of studies** | **No. of treatments** | **No. of participants** | **No. of events** | **Aggregate event rate** |
| --- | --- | --- | --- | --- | --- |
| Major cardiovascular outcome (composite) | 33 | 14 | 92,469 | 12,328 | 13.3 |
| Cardiovascular mortality | 41 | 14 | 95,060 | 6,166 | 6.5 |
| Myocardial infarction | 48 | 14 | 84,792 | 4,593 | 5.4 |
| Stroke | 42 | 14 | 95,115 | 4,591 | 4.8 |
| Heart failure | 33 | 13 | 81,373 | 5,272 | 6.5 |
| Angina pectoris | 30 | 10 | 65,656 | 5,026 | 7.7 |
| Progression of renal disease (composite) | 18 | 12 | 69,380 | 9,267 | 13.4 |
| End-stage renal disease | 22 | 12 | 67,316 | 1,786 | 2.7 |
| Doubling of serum creatinine level | 24 | 13 | 67,505 | 2,645 | 3.9 |
| All-cause mortality | 59 | 14 | 101,369 | 11,199 | 11.0 |
| Total | 71 | 14 | 103,120 | - | - |
