# Supplementary material for: Cardiovascular and Renal Outcomes of Renin–Angiotensin System Blockade in Adult Patients with Diabetes Mellitus: A Systematic Review with Network Meta-Analyses
Source: PLoS Med. 2016 Mar 8;13(3):e1001971. doi: 10.1371/journal.pmed.1001971 (PMC4783064; doi:10.1371/journal.pmed.1001971)
Supplement: S6 Table — (DOCX) [file pmed.1001971.s009.docx]

**S6 Table. Number of cardiovascular events per trial and treatment comparison.**

| **Trial name, year** | **Treatment comparisons** | **Cardiovascular composite** | **Cardiovascular death** | **Myocardial infarction** | **Stroke** | **Heart failure** | **Angina** |
| --- | --- | --- | --- | --- | --- | --- | --- |
| Parving et al 1989^1,2^ | ACEi; placebo | - | - | - | - | - | - |
| Bauer et al 1992^3^ | ACEi; placebo | - | - | 1/18; 0/15 | 0/18; 1/15 | - | - |
| Björck et al 1992^4^ | ACEi; β-blocker | - | - | 2/22; 0/18 | - | - | - |
| Chan et al 1992^5^ | ACEi; CCB | - | 1/50; 0/52 | 1/50; 0/52 | - | - | 1/50; 1/52 |
| Lacourcière et al 1993^6^ | ACEi; control | - | 3/50; 0/59 | 3/50; 2/59 | - | - | 0/50; 1/59 |
| Lewis et al 1993^7^ | ACEi; placebo | - | - | - | - | - | - |
| Ravid et al 1993^8^ | ACEi; placebo | - | - | - | - | - | - |
| Elving et al 1994^9^ | ACEi; β-blocker | - | - | 1/15; 1/15 | - | - | - |
| Sano et al 1994^10^ | ACEi; control | - | - | - | - | - | - |
| Laffel et al 1995^11^ | ACEi; placebo | - | - | - | - | - | - |
| Bakris et al 1996^12^ | ACEi; β-blocker; CCB | - | 1/18; 3/16; 1/18 | - | - | - | - |
| Viberti et al 1996^13^ | ACEi; placebo | - | - | - | - | - | - |
| Nielsen et al 1997^14,15^ | ACEi; β-blocker | - | 1/21; 3/22 | 0/21; 2/22 | - | - | - |
| ABCD-Hypertension 1998^16,17^ | ACEi; CCB | - | 6/235; 11/235 | 9/235; 27/235 | 7/235; 11/235 | 10/235; 8/235 | - |
| ABCD-normo 2002^18^ | ACEi; CCB | - | 14/246; 8/234 | 16/246; 18/234 | 6/246; 11/234 | 12/246; 11/234 | - |
| Crepaldi et al 1998^19^ | ACEi; CCB; placebo | - | - | 0/32; 0/26; 1/34 | - | - | - |
| FACET 1998^20^ | ACEi; CCB | 14/189; 23/191 | - | 10/189; 13/191 | 4/189; 10/191 | - | 0/189; 4/191 |
| Nankervis et al 1998^21^ | ACEi; placebo | - | - | - | - | - | - |
| Ravid et al 1998^22^ | ACEi; placebo | - | - | - | - | - | - |
| UKPDS-39  1998^23^ | ACEi; β-blocker | 81/400; 80/358 | 48/400; 32/358 | 61/400; 46/358 | 21/400; 17/358 | 12/400; 9/358 | 20/400; 25/358 |
| Fogari et al 1999^24^ | ACEi; CCB | - | - | - | - | - | - |
| ATLANTIS 2000^25^ | ACEi; placebo | - | - | 3/92; 1/48 | - | - | 4/92; 5/48 |
| Tarnow et al 2000^26^ | ACEi; CCB | 3/25; 8/27 | 0/25; 2/27 | 2/25; 1/27 | 1/25; 5/27 | - | 1/25; 0/27 |
| Chan et al 2000^27^ | ACEi; CCB | - | 2/50; 2/52 | - | - | - | - |
| STOP HTN-2 2000^28^ | ACEi; CCB; control | 81/235; 79/231; 92/253 | 39/235; 33/231; 45/253 | 17/235; 32/231; 26/253 | 34/235; 29/231; 39/253 | 22/235; 24/231; 29/253 | - |
| Micro-HOPE 2000^29^ | ACEi; placebo | 277/1808; 351/1769 | 112/1808; 172/1769 | 185/1808; 229/1769 | 76/1808; 108/1769 | 81/1808; 79/1769 | 363/1808; 397/1769 |
| J-MIND 2001^30^ | ACEi; CCB | - | - | 1/208; 1/228 | 5/208; 2/228 | 0/208; 1/228 | 2/208; 0/228 |
| IDNT 2001^31,32^ | ARB; CCB; placebo | 124/579; 79/567; 118/569 | 52/579; 37/567; 118/569 | 44/579; 27/567; 46/569 | 28/579; 15/567; 26/569 | 58/579; 86/567; 71/569 | 58/579; 60/567; 66/569 |
| IRMA-2 2001^33^ | ARB; placebo | 11/402; 9/206 | 5/402; 1/206 | 6/402; 5/206 | 3/402; 3/206 | 7/402; 1/206 | 11/402; 6/206 |
| Jerums et al 2001^34^ | ACEi; CCB; placebo | - | - | - | - | - | - |
| RENAAL 2001^35,36^ | ARB; placebo | 188/751; 197/762 | 90/751; 79/762 | 50/751; 68/762 | 47/751; 50/762 | 89/751; 127/762 | 42/751; 41/762 |
| CAPPP 2001^37^ | ACEi; control | 35/309; 46/263 | 9/309; 15/263 | 12/309; 27/263 | 23/309; 19/263 | 11/309; 17/263 | - |
| Val-HeFT 2001^38^ | ACEi + ARB; ACEi | - | - | - | - | 126/609; 132/576 | 43/609; 51/576 |
| Fogari et al 2002^39^ | ACEi + CCB; ACEi; CCB | 4/104; 7/102; 8/103 | 1/104; 2/102; 2/103 | 1/104; 3/102; 4/103 | 1/104; 3/102; 2/103 | - | - |
| JAPAN-IDDM 2002^40^ | ACEi; placebo | - | - | - | - | - | - |
| LIFE 2002^41^ | ARB; β-blocker | 103/586; 139/609 | 38/586; 61/609 | 41/586; 50/609 | 51/586; 65/609 | 32/586; 55/609 | 30/586; 30/609 |
| VALIANT 2003^42^ | ACEi + ARB; ACEi; ARB | 364/1146; 370/1120; 392/1134 | 261/1146; 261/1120; 286/1134 | 177/1146; 173/1120; 203/1134 | 36/1146; 53/1120; 55/1134 | 274/1146; 281/1120; 296/1134 | 239/1146; 202/1120; 251/1134 |
| VALUE 2004^43^ | ARB; CCB | 349/2395; 317/2428 | 137/2395; 135/2428 | 150/2395; 134/2428 | 122/2395; 103/2428 | 175/2395; 209/2428 | 221/2395; 160/2428 |
| BENEDICT 2004^44^ | ACEi + CCB; ACEi; CCB; placebo | - | 0/300; 1/301; 1/303; 3/300 | - | - | - | - |
| DETAIL 2004^45,46^ | ACEi; ARB | 14/130; 18/120 | 2/130; 3/120 | 8/130; 10/120 | 6/130; 6/120 | 7/130; 9/120 | - |
| DIABHYCAR 2004^47^ | ACEi; placebo | 282/2443; 276/2469 | 141/2443; 133/2469 | 61/2443; 78/2469 | 118/2443; 116/2469 | 85/2443; 102/2469 | 35/2443; 61/2469 |
| NESTOR 2004^48^ | ACEi; diuretic | 1/286; 8/284 | 1/286; 2/284 | 0/286; 3/284 | 0/286; 4/284 | - | 0/286; 2/284 |
| JMIC-B 2004^49^ | ACEi; CCB | 13/173; 9/199 | 3/173; 1/199 | 4/173; 4/199 | 6/173; 4/199 | 5/173; 8/199 | 12/173; 16/199 |
| Ko et al 2005^50^ | ACEi; ARB | - | - | - | 1/20; 0/22 | - | - |
| Schram et al 2005^51^ | ACEi; ARB; diuretic | - | - | 0/22; 0/24; 2/22 | - | - | - |
| PERSUADE 2005^52^ | ACEi; placebo | 103/721; 130/781 | 47/721; 60/781 | 56/721; 78/781 | 18/721; 23/781 | 13/721; 26/781 | - |
| ALLHAT 2005^53,54^ | ACEi; CCB; diuretic | 557/3532; 553/3612; 968/6024 | 304/3532; 320/3612; 517/6024 | 253/3532, 261/3612; 435/6024 | 210/3532; 184/3612; 338/6024 | 220/3532; 281/3612; 352/6024 | 294/3532; 275/3612; 468/6024 |
| SCOPE 2005^55^ | ARB; control | 46/313; 51/286 | - | - | 17/313; 17/286 | - | - |
| ABCD-2V 2006^56^ | ARB; control | - | - | - | - | - | - |
| Tong et al 2006^57^ | ACEi; placebo | - | - | - | - | - | - |
| ADVANCE 2007^58,59^ | ACEi + diuretic; placebo | 480/5569; 520/5571 | 211/5569; 257/5571 | - | 286/5569; 303/5571 | - | - |
| DIRECT-Prevent 1 2008^60,61^ | ARB; placebo | 3/711; 7/710 | 0/711; 2/710 | 0/711; 5/710 | 3/711; 1/710 | 0/711; 1/710 | 4/711; 6/710 |
| DIRECT-Protect 1 2008^60,61^ | ARB; placebo | 3/951; 5/954 | 1/951; 1/954 | 1/951; 3/954 | 2/951; 2/954 | Not reported | 6/951; 9/954 |
| DIRECT-Protect 2 2008^60,62,63^ | ARB; placebo | 51/951; 65/954 | 13/951; 17/954 | 29/951; 36/954 | 21/951; 28/954 | 6/951; 7/954 | 51/951; 40/954 |
| GUARD 2008^64^ | ACEi + CCB; ACEi + diuretic | - | - | - | - | - | - |
| PRoFESS 2008^65^ | ARB; placebo | 491/2840; 502/2903 | 184/2840; 161/2903 | 89/2840; 79/2903 | 316/2840; 330/2903 | 82/2840; 82/2903 | - |
| ONTARGET 2008^66-68^ | ACEi + ARB; ACEi; ARB | 533/3220; 546/3146; 539/3246 | 300/3220; 281/3146; 290/3246 | 179/3220; 192/3146; 193/3246 | 168/3220; 177/3146; 169/3246 | 175/3220; 189/3146; 224/3246 | 327/3220; 352/3146; 353/3246 |
| TRANSCEND 2008^69-71^ | ARB; placebo | 169/1059; 188/1059 | 99/1059; 96/1059 | 52/1059; 56/1059 | 48/1059; 58/1059 | 66/1059; 52/1059 | 109/1059; 119/1059 |
| Kohlmann Jr et al 2009^72^ | ACEi + CCB; ARB + diuretic | - | - | 1/54; 1/56 | - | - | - |
| Mehdi et al 2009^73^ | ACEi + ARB; ACEi + diuretic; ACEi | - | - | 0/26; 1/27; 0/27 | 1/26; 2/27; 1/27 | 2/26; 2/27; 0/27 | - |
| RAAS 2009^74^ | ACEi; ARB; placebo | - | - | - | - | - | - |
| CASE-J 2010^75^ | ARB; CCB | 50/1011; 48/1007 | 11/1011; 15/1007 | 14/1011; 15/1007 | 30/1011; 23/1007 | - | 6/1011, 8/1007 |
| ROADMAP 2011^76^ | ARB; placebo | 29/2232; 16/2215 | 15/2232; 3/2215 | 5/2232; 5/2215 | 16/2232; 10/2215 | 0/2232; 7/2215 | 40/2232; 55/2215 |
| ORIENT 2011^77,78^ | ACEi + ARB; ACEi; ARB; placebo | 16/205; 14/209; 5/75; 7/74 | 7/205; 2/209; 3/75; 1/74 | 3/205; 5/209; 0/75; 2/74 | 6/205; 7/209; 2/75; 4/74 | 13/205; 19/209; 5/75; 6/74 | 3/205; 3/209; 2/75; 0/74 |
| DEMAND 2011^79^ | ACEi + CCB; ACEi; placebo | - | 0/126; 0/127; 2/127 | - | - | - | - |
| ALTITUDE 2012^80^ | DRi + ACEi; DRi + ARB; ACEi; ARB | 234/1926; 224/2353; 209/1864; 201/2436 | 136/1926; 115/2353; 113/1864; 106/2436 | 84/1926; 64/2353; 74/1864; 70/2436 | 65/1926; 86/2353; 58/1864; 66/2436 | 106/1926; 103/2353; 112/1864; 111/2436 | - |
| NAGOYA HEART 2012^81,82^ | ARB; CCB | - | - | 7/575; 3/575 | 13/575; 16/575 | 3/575; 15/575 | - |
| VA NEPHRON-D  2013^83^ | ACEi + ARB; ARB | - | - | 52/724; 40/724 | 18/724; 18/724 | 89/724; 106/724 | - |
| ASTRONAUT 2013^84,85^ | DRi + diuretic; diuretic | 69/319; 74/343 | 62/319; 52/343 | 11/319; 21/343 | 6/319; 13/343 | 108/319; 108/343 | 16/319; 13/343 |
| COLM 2014^86^ | ARB + CCB; ARB + diuretic | 26/684; 26/678 | 4/684; 7/678 | 3/684; 8/678 | 24/684; 16/678 | 7/684; 5/678 | 1/684; 2/678 |
| OSCAR 2014^87,88^ | ARB + CCB; diuretic | - | 1/319; 2/309 | 3/319; 1/309 | 10/319; 8/309 | 3/319; 5/309 | 2/319; 1/309 |
