# Supplementary material for: Cardiovascular and Renal Outcomes of Renin–Angiotensin System Blockade in Adult Patients with Diabetes Mellitus: A Systematic Review with Network Meta-Analyses
Source: PLoS Med. 2016 Mar 8;13(3):e1001971. doi: 10.1371/journal.pmed.1001971 (PMC4783064; doi:10.1371/journal.pmed.1001971)
Supplement: S7 Table — (DOCX) [file pmed.1001971.s010.docx]

**S7 Table. Number of deaths and renal events per trial and treatment comparison.**

| **Trial name, year** | **Treatment comparisons** | **Deaths** | **Renal composite** | **ESRD** | **Doubling CrS** |
| --- | --- | --- | --- | --- | --- |
| Parving et al 1989^1,2^ | ACEi; placebo | 1/15; 1/17 | - | 0/15; 3/17 | 2/15; 3/17 |
| Bauer et al 1992^3^ | ACEi; placebo | 1/18; 0/15 | - | 1/18; 0/15 | - |
| Björck et al 1992^4^ | ACEi; β-blocker | - | - | 2/22; 2/18 | - |
| Chan et al 1992^5^ | ACEi; CCB | 1/50; 0/52 | - | - | - |
| Lacourcière et al 1993^6^ | ACEi; control | 4/50; 0/59 | - | - | - |
| Lewis et al 1993^7^ | ACEi; placebo | 8/207; 14/202 | - | 20/207; 31/202 | 25/207; 43/202 |
| Ravid et al 1993^8^ | ACEi; placebo | - | - | - | 2/56; 12/52 |
| Elving et al 1994^9^ | ACEi; β-blocker | - | - | - | - |
| Sano et al 1994^10^ | ACEi; control | 1/26; 0/26 | - | - | - |
| Laffel et al 1995^11^ | ACEi; placebo | 1/70; 0/73 | - | - | - |
| Bakris et al 1996^12^ | ACEi; β-blocker; CCB | 1/18; 4/16; 1/18 | 2/18; 9/16; 3/18 | 0/18; 4/16; 0/18 | 1/18; 5/16; 2/18 |
| Viberti et al 1996^13^ | ACEi; placebo | 1/116; 1/119 | - | - | - |
| Nielsen et al 1997^14,15^ | ACEi; β-blocker | 1/21; 5/22 | - | 1/21; 1/22 | - |
| ABCD-Hypertension 1998^16,17^ | ACEi; CCB | 14/235; 18/235 | - | - | - |
| ABCD-normo 2002^18^ | ACEi; CCB | 19/246; 19/234 | - | - | - |
| Crepaldi et al 1998^19^ | ACEi; CCB; placebo | - | - | - | - |
| FACET 1998^20^ | ACEi; CCB | 4/189; 5/191 | - | - | - |
| Nankervis et al 1998^21^ | ACEi; placebo | 0/20; 3/20 | - | - | - |
| Ravid et al 1998^22^ | ACEi; placebo | 3/97; 2/97 | - | - | 3/97; 8/79 |
| UKPDS-39  1998^23^ | ACEi; β-blocker | 75/400; 59/358 | - | - | - |
| Fogari et al 1999^24^ | ACEi; CCB | - | - | 1/54; 1/53 | - |
| ATLANTIS 2000^25^ | ACEi; placebo | 5/92; 0/48 | - | - | - |
| Tarnow et al 2000^26^ | ACEi; CCB | 0/25; 3/27 | - | 2/25; 3/27 | - |
| Chan et al 2000^27^ | ACEi; CCB | 2/50; 2/52 | - | - | - |
| STOP HTN-2 2000^28^ | ACEi; CCB; control | 56/235; 50/231; 67/253 | - | - | - |
| Micro-HOPE 2000^29^ | ACEi; placebo | 196/1808; 248/1769 | - | 10/1808; 8/1769 | - |
| J-MIND 2001^30^ | ACEi; CCB | - | - | - | - |
| IDNT 2001^31,32^ | ARB; CCB; placebo | 87/579; 83/567; 93/569 | 189/579; 233/567; 222/569 | 82/579; 104/567; 101/569 | 98/579; 144/567; 135/569 |
| IRMA-2 2001^33^ | ARB; placebo | 11/402; 5/206 | - | - | - |
| Jerums et al 2001^34^ | ACEi; CCB; placebo | 0/17; 1/13; 0/12 | - | - | - |
| RENAAL 2001^35,36^ | ARB; placebo | 158/751; 155/762 | 327/751; 359/762 | 147/751; 194/762 | 162/751; 198/762 |
| CAPPP 2001^37^ | ACEi; control | 20/309; 34/263 | - | - | - |
| Val-HeFT 2001^38^ | ACEi + ARB; ACEi | 139/609; 125/576 | - | - | - |
| Fogari et al 2002^39^ | ACEi + CCB; ACEi; CCB | 2/104; 3/102; 4/103 | - | - | - |
| JAPAN-IDDM 2002^40^ | ACEi; placebo | - | - | - | 2/52; 2/27 |
| LIFE 2002^41^ | ARB; β-blocker | 63/586; 104/609 | - | - | - |
| VALIANT 2003^42^ | ACEi + ARB; ACEi; ARB | 298/1146; 294/1120; 325/1134 | - | - | - |
| VALUE 2004^43^ | ARB; CCB | 330/2395; 314/2428 | - | 28/2395; 21/2428 | - |
| BENEDICT 2004^44^ | ACEi + CCB; ACEi; CCB; placebo | 2/300; 4/301; 2/303; 5/300 | - | - | - |
| DETAIL 2004^45,46^ | ACEi; ARB | 6/130; 6/120 | - | - | - |
| DIABHYCAR 2004^47^ | ACEi; placebo | 334/2443; 324/2469 | 386/2443; 394/2469 | 11/2443; 12/2469 | 48/2443; 60/2469 |
| NESTOR 2004^48^ | ACEi; diuretic | 1/286; 2/284 | - | - | - |
| JMIC-B 2004^49^ | ACEi; CCB | 5/173; 2/199 | - | - | - |
| Ko et al 2005^50^ | ACEi; ARB | - | - | - | - |
| Schram et al 2005^51^ | ACEi; ARB; diuretic | - | - | - | - |
| PERSUADE 2005^52^ | ACEi; placebo | 73/721; 93/781 | 80/721; 101/781 | - | 10/721; 11/781 |
| ALLHAT 2005^53,54*^ | ACEi; CCB; diuretic | 612/3532; 619/3612; 1079/6024 | 705/3532; 704/3612; 1241/6024 | 77/3532; 93/3612; 120/6024 | 84/2972; 79/3051; 139/5196 |
| SCOPE 2005^55^ | ARB; control | - | - | - | - |
| ABCD-2V 2006^56^ | ARB; control | 1/66; 0/63 | - | - | - |
| Tong et al 2006^57^ | ACEi; placebo | - | - | - | 4/18; 5/20 |
| ADVANCE 2007^58,59^ | ACEi + diuretic; placebo | 408/5569; 471/5571 | 488/5569; 537/5571 | 25/5569; 21/5571 | 55/488; 45/5571 |
| DIRECT-Prevent 1 2008^60,61^ | ARB; placebo | 7/711; 5/710 | 10/711; 6/710 | - | 10/711; 4/710 |
| DIRECT-Protect 1 2008^60,61^ | ARB; placebo | 7/951; 8/954 | 12/951; 8/954 | - | 12/951; 7/954 |
| DIRECT-Protect 2 2008^60,62,63^ | ARB; placebo | 37/951; 35/954 | 23/951; 13/954 | - | 17/951; 12/954 |
| GUARD 2008^64^ | ACEi + CCB; ACEi + diuretic | 1/166; 2/166 | - | - | - |
| PRoFESS 2008^65^ | ARB; placebo | 284/2840; 249/2903 | - | - | - |
| ONTARGET 2008^66-68^ | ACEi + ARB; ACEi; ARB | 485/3220; 458/3146; 455/3246 | 585/3220; 545/3146; 554/3246 | 62/3220; 61/3146; 50/3246 | 94/3220; 87/3146; 92/3246 |
| TRANSCEND 2008^69-71^ | ARB; placebo | 156/1059; 141/1059 | 181/1059; 160/1059 | 5/1059; 9/1059 | 27/1059; 21/1059 |
| Kohlmann Jr et al 2009^72^ | ACEi + CCB; ARB + diuretic | - | - | - | - |
| Mehdi et al 2009^73^ | ACEi + ARB; ACEi + diuretic; ACEi | 0/26; 1/27; 0/27 | - | - | - |
| RAAS 2009^74^ | ACEi; ARB; placebo | 1/94; 1/96; 1/95 | - | - | - |
| CASE-J 2010^75^ | ARB; CCB | 40/1011; 49/1007 | 17/1011; 21/1007 | 4/1011; 9/1007 | 17/1011; 20/1007 |
| ROADMAP 2011^76^ | ARB; placebo | 26/2232; 15/2215 | 49/2232; 38/2215 | - | 23/2232; 23/2215 |
| ORIENT 2011^77,78^ | ACEi + ARB; ACEi; ARB; placebo | 14/205; 16/209; 5/75; 4/74 | 80/205; 87/209; 35/75; 42/74 | 48/205; 49/209; 25/75; 29/74 | 72/205; 80/209; 33/75; 40/74 |
| DEMAND 2011^79^ | ACEi + CCB; ACEi; placebo | 0/126; 0/127; 2/127 | - | - | - |
| ALTITUDE 2012^80**^ | DRi + ACEi; DRi + ARB; ACEi; ARB | 202/1926; 179/2353; 176/1864; 188/2436 | 108/1926; 155/2353; 95/1864; 163/2436 | 53/1926; 69/2353; 43/1864; 71/2436 | 82/1926; 134/2353; 81/1864; 142/2436 |
| NAGOYA HEART 2012^81,82^ | ARB; CCB | 22/575; 16/575 | - | - | - |
| VA NEPHRON-D  2013^83^ | ACEi + ARB; ARB | 63/724; 60/724 | 132/724; 152/724 | 27/724; 43/724 | - |
| ASTRONAUT 2013^84,85^ | DRi + diuretic; diuretic | 72/319; 57/343 | - | - | 65/319; 49/343 |
| COLM 2014^86^ | ARB + CCB; ARB + diuretic | 22/684; 29/678 | 8/684; 8/678 | 0/684; 3/678 | 8/684; 6/678 |
| OSCAR 2014^87,88^ | ARB + CCB; diuretic | 7/319; 8/309 | - | - | - |

*For ALLHAT, sample size for the doubling CrS endpoint was reduced due to missing baseline and follow-up creatinine values (according to trialists). For ALTITUDE, data for the renal composite endpoint included: doubling CrS, ESRD and/or renal death. ** For ALTITUDE, outcomes are based on the longest follow-up period (e.g. at the end of study) including upublished data from the study sponsor. Used data are somewhat discrepant with those from published article in the New England Journal of Medicine (where a lower number of events were reported).
