# Supplementary material for: Cardiovascular and Renal Outcomes of Renin–Angiotensin System Blockade in Adult Patients with Diabetes Mellitus: A Systematic Review with Network Meta-Analyses
Source: PLoS Med. 2016 Mar 8;13(3):e1001971. doi: 10.1371/journal.pmed.1001971 (PMC4783064; doi:10.1371/journal.pmed.1001971)
Supplement: S8 Table — Summary ORs and CrIs. (DOCX) [file pmed.1001971.s011.docx]

**S8 Table. Main results for all possible treatment comparisons. Summary odds ratios (OR) and credible intervals.**

**Table 8a. Major cardiovascular outcome (composite endpoint) and all possible treatment comparisons.**

**Summary odds ratios (OR) and credible intervals from network meta-analysis.**

| **Pbo/control** |  |  |  |  |  |  |  |  |  |  |  |  |  |
| --- | --- | --- | --- | --- | --- | --- | --- | --- | --- | --- | --- | --- | --- |
| **0.87**  **(0.75-0.99)** | **ACEi** |  |  |  |  |  |  |  |  |  |  |  |  |
| 1.12  (0.81-1.54) | 1.29  (0.95-1.76) | **BB** |  |  |  |  |  |  |  |  |  |  |  |
| **0.81**  **(0.67-0.97)** | 0.93  (0.79-1.12) | 0.72  (0.52-1.02) | **CCB** |  |  |  |  |  |  |  |  |  |  |
| 0.89  (0.78-1.01) | 1.02  (0.90-1.18) | 0.80  (0.59-1.09) | 1.10  (0.93-1.30) | **ARB** |  |  |  |  |  |  |  |  |  |
| 0.41  (0.11-1.22) | 0.47  (0.12-1.41) | 0.36  (0.09-1.14) | 0.50  (0.13-1.51) | 0.46  (0.12-1.37) | **ACEi+CCB** |  |  |  |  |  |  |  |  |
| 0.92  (0.69-1.28) | 1.06  (0.82-1.47) | 0.82  (0.56-1.28) | 1.14  (0.87-1.55) | 1.03  (0.79-1.43) | 2.27  (0.74-8.95) | **Diuretic** |  |  |  |  |  |  |  |
| 0.91  (0.66-1.26) | 1.05  (0.75-1.52) | 0.82  (0.52-1.30) | 1.13  (0.78-1.64) | 1.03  (0.73-1.46) | 2.26  (0.72-8.94) | 1.00  (0.62-1.51) | **ACEi+diuretic** |  |  |  |  |  |  |
| NA | NA | NA | NA | NA | NA | NA | NA | **ARB+diuretic** |  |  |  |  |  |
| 0.84  (0.67-1.05) | 0.97  (0.79-1.19) | 0.75  (0.53-1.08) | 1.04  (0.80-1.33) | 0.94  (0.77-1.16) | 2.07  (0.67-7.94) | 0.91  (0.63-1.26) | 0.92  (0.62-1.35) | **NA** | **ACEi+ARB** |  |  |  |  |
| 1.15  (0.82-1.58) | 1.32  (0.96-1.81) | 1.02  (0.67-1.57) | 1.41  (0.99-1.98) | 1.29  (0.93-1.75) | 2.81  (0.90-11.22) | 1.25  (0.79-1.84) | 1.25  (0.78-1.96) | **NA** | 1.37  (0.94-1.95) | **DRi+ACEi** |  |  |  |
| 0.87  (0.62-1.20) | 1.00  (0.73-1.38) | 0.78  (0.51-1.20) | 1.08  (0.75-1.51) | 0.98  (0.71-1.33) | 2.15  (0.69-8.45) | 0.95  (0.60-1.40) | 0.95  (0.59-1.50) | **NA** | 1.04  (0.72-1.48) | 0.76  (0.54-1.08) | **DRi+ARB** |  |  |
| 0.93  (0.54-1.66) | 1.07  (0.63-1.91) | 0.83  (0.45-1.60) | 1.14  (0.67-2.03) | 1.04  (0.60-1.86) | 2.29  (0.67-9.73) | 1.00  (0.63-1.62) | 1.01  (0.54-1.97) | **NA** | 1.10  (0.62-2.02) | 0.81  (0.44-1.56) | 0.58  (0.00-1.06) | **DRi+diuretic** |  |
| NA | NA | NA | NA | NA | NA | NA | NA | 0.99  (0.52-1.84) | **NA** | **NA** | **NA** | **NA** | **ARB+CCB** |

Comparisons between treatments should be read from left to right. The treatment at the top of each column is the reference group for comparisons in that column, while the comparator is the lower treatment in the step-ladder. For major cardiovascular outcome, odds ratios (ORs) < than 1 suggests fewer outcomes with the comparator than with the reference group. Statistically significant results are in bold and underlined. Comparisons involving any renin-angiotensin receptor blocker are highlighted in light green. Comparisons of renin-angiotensin blockers against each other are highlighted in dark green.

NA = not available. Effect sizes for treatment comparisons including ARB+diuretic and/or ARB+CCB were generally unstable and uninterpretable owing to small sample sizes, rare events and lack of direct evidence.

Pbo/control = placebo or control; ACEi = Angiotensin converting enzyme inhibitor; BB = beta-blocker; CCB = Calcium channel blocker; ARB = Angiotensin receptor blocker; ACEi+CCB = Angiotensin converting enzyme inhibitor + calcium channel blocker; ACEi+CCB = Angiotensin converting enzyme inhibitor + diuretic; ACEi+ARB = Angiotensin converting enzyme inhibitor + angiotensin receptor blocker; DRi+ACEi = Direct renin inhibitor (aliskiren) + angiotensin converting enzyme inhibitor; DRi+ARB = Direct renin inhibitor (aliskiren) + angiotensin receptor blocker; DRi+diuretic = Direct renin inhibitor (aliskiren) + diuretic.

**Table 8b. Cardiovascular mortality and all possible treatment comparisons.**

**Summary odds ratios (OR) and credible intervals from network meta-analysis.**

| **Pbo/control** |  |  |  |  |  |  |  |  |  |  |  |  |  |
| --- | --- | --- | --- | --- | --- | --- | --- | --- | --- | --- | --- | --- | --- |
| 0.93  (0.75-1.14) | **ACEi** |  |  |  |  |  |  |  |  |  |  |  |  |
| 1.17  (0.74-1.91) | 1.26  (0.81-2.01) | **BB** |  |  |  |  |  |  |  |  |  |  |  |
| 0.89  (0.67-1.16) | 0.96  (0.74-1.23) | 0.76  (0.45-1.22) | **CCB** |  |  |  |  |  |  |  |  |  |  |
| 1.00  (0.82-1.23) | 1.07  (0.88-1.33) | 0.85  (0.54-1.33) | 1.12  (0.87-1.48) | **ARB** |  |  |  |  |  |  |  |  |  |
| **0.14**  **(0.01-0.83)** | **0.15**  **(0.01-0.88)** | **0.12**  **(0.01-0.75)** | **0.16**  **(0.01-0.93)** | **0.14**  **(0.01-0.83)** | **ACEi+CCB** |  |  |  |  |  |  |  |  |
| 0.91  (0.57-1.45) | 0.98  (0.64-1.52) | 0.78  (0.41-1.42) | 1.02  (0.67-1.60) | 0.91  (0.57-1.44) | **6.55**  **(1.05-131)** | **Diuretic** |  |  |  |  |  |  |  |
| 0.81  (0.49-1.36) | 0.87  (0.51-1.52) | 0.70  (0.34-1.38) | 0.92  (0.52-1.64) | 0.82  (0.47-1.39) | 5.88  (0.91-119) | 0.90  (0.45-1.78) | **ACEi+diuretic** |  |  |  |  |  |  |
| 0.71  (0.02-14.19) | 0.77  (0.02-15.2) | 0.61  (0.02-12.4) | 0.81  (0.02-16.1) | 0.71  (0.02-14.1) | 5.57  (0.09-402) | 0.79  (0.02-16.12) | 0.88  (0.03-18.26) | **ARB+diuretic** |  |  |  |  |  |
| 0.99  (0.71-1.42) | 1.06  (0.79-1.49) | 0.84  (0.50-1.43) | 1.11  (0.78-1.66) | 0.99  (0.73-1.37) | **7.13**  **(1.17-141)** | 1.08  (0.65-1.86) | 1.21  (0.67-2.29) | 1.39  (0.07-43.71) | **ACEi+ARB** |  |  |  |  |
| 1.35  (0.82-2.22) | 1.45  (0.91-2.35) | 1.16  (0.60-1.16) | 1.52  (0.91-2.58) | 1.35  (0.84-2.16) | **9.80**  **(1.54-197)** | 1.49  (0.79-2.80) | 1.66  (0.82-3.38) | 1.90  (0.09-61.25) | 1.37  (0.77-2.34) | **DRi+ACEi** |  |  |  |
| 0.91  (0.55-1.51) | 0.98  (0.61-1.60) | 0.78  (0.40-1.46) | 1.03  (0.61-1.74) | 0.91  (0.56-1.47) | **6.61**  **(1.04-133)** | 1.00  (0.53-1.90) | 1.12  (0.55-2.30) | 1.28  (0.06-40.53) | 0.92  (0.52-1.59) | 0.67  (0.39-1.15) | **DRi+ARB** |  |  |
| 1.23  (0.57-2.67) | 1.32  (0.63-2.84) | 1.05  (0.43-2.48) | 1.38  (0.66-2.98) | 1.23  (0.57-2.64) | **8.95**  **(1.29-185)** | 1.35  (0.73-2.52) | 1.51  (0.60-3.84) | 1.73  (0.08-60.01) | 1.24  (0.55-2.76) | 0.91  (0.38-2.20) | 1.35  (0.56-3.27) | **DRi+diuretic** |  |
| 0.38  (0.01-5.35) | 0.41  (0.02-5.75) | 0.33  (0.01-4.70) | 0.43  (0.02-6.08) | 0.38  (0.02-5.26) | 2.85  (0.06-168) | 0.42  (0.02-6.02) | 0.47  (0.02-6.91) | 0.53  (0.12-1.98) | 0.39  (0.02-5.49) | 0.28  (0.01-4.08) | 0.42  (0.02-5.99) | 0.31  (0.01-4.79) | **ARB+CCB** |

Comparisons between treatments should be read from left to right. The treatment at the top of each column is the reference group for comparisons in that column, while the comparator is the lower treatment in the step-ladder. For cardiovascular mortality, odds ratios (ORs) < than 1 suggests fewer outcomes with the comparator than with the reference group. Statistically significant results are in bold and underlined. Comparisons involving any renin-angiotensin receptor blocker are highlighted in light green. Comparisons of renin-angiotensin blockers against each other are highlighted in dark green.

Pbo/control = placebo or control; ACEi = Angiotensin converting enzyme inhibitor; BB = beta-blocker; CCB = Calcium channel blocker; ARB = Angiotensin receptor blocker; ACEi+CCB = Angiotensin converting enzyme inhibitor + calcium channel blocker; ACEi+CCB = Angiotensin converting enzyme inhibitor + diuretic; ACEi+ARB = Angiotensin converting enzyme inhibitor + angiotensin receptor blocker; DRi+ACEi = Direct renin inhibitor (aliskiren) + angiotensin converting enzyme inhibitor; DRi+ARB = Direct renin inhibitor (aliskiren) + angiotensin receptor blocker; DRi+diuretic = Direct renin inhibitor (aliskiren) + diuretic.

**Table 8c. Myocardial infarction and all possible treatment comparisons.**

**Summary odds ratios (OR) and credible intervals from network meta-analysis.**

| **Pbo/control** |  |  |  |  |  |  |  |  |  |  |  |  |  |
| --- | --- | --- | --- | --- | --- | --- | --- | --- | --- | --- | --- | --- | --- |
| **0.77**  **(0.62-0.92)** | **ACEi** |  |  |  |  |  |  |  |  |  |  |  |  |
| 0.78  (0.50-1.19) | 1.01  (0.68-1.52) | **BB** |  |  |  |  |  |  |  |  |  |  |  |
| 0.84  (0.65-1.09) | 1.09  (0.89-1.39) | 1.08  (0.70-1.72) | **CCB** |  |  |  |  |  |  |  |  |  |  |
| **0.82**  **(0.67-0.98)** | 1.07  (0.89-1.28) | 1.05  (0.69-1.58) | 0.98  (0.75-1.21) | **ARB** |  |  |  |  |  |  |  |  |  |
| 0.35  (0.05-1.68) | 0.45  (0.06-2.18) | 0.45  (0.06-2.24) | 0.41  (0.06-1.97) | 0.42  (0.06-2.05) | **ACEi+CCB** |  |  |  |  |  |  |  |  |
| 0.86  (0.59-1.40) | 1.12  (0.81-1.82) | 1.11  (0.67-2.09) | 1.03  (0.73-1.61) | 1.05  (0.75-1.75) | 2.52  (0.51-19.4) | **Diuretic** |  |  |  |  |  |  |  |
| NA | NA | NA | NA | NA | NA | **NA** | **ACEi+diuretic** |  |  |  |  |  |  |
| 2.34  (0.28-30.5) | 3.06  (0.37-39.8) | 3.02  (0.35-40.48) | 2.79  (0.33-36.0) | 2.86  (0.35-36.9) | 6.91  (0.65-135) | 2.66  (0.31-35.2) | **NA** | **ARB+diuretic** |  |  |  |  |  |
| 0.78  (0.57-1.03) | 1.00  (0.78-1.33) | 0.99  (0.62-1.59) | 0.92  (0.65-1.24) | 0.94  (0.73-1.23) | 2.21  (9.46-16.7) | 0.89  (0.52-1.33) | **NA** | 0.33  (0.03-2.75) | **ACEi+ARB** |  |  |  |  |
| 1.04  (0.64-1.64) | 1.35  (0.85-2.12) | 1.33  (0.73-2.40) | 1.24  (0.74-1.97) | 1.27  (0.80-1.99) | 2.98  (0.58-23.1) | 1.20  (0.61-2.04) | **NA** | 0.44  (0.03-3.83) | 1.35  (0.80-2.21) | **DRi+ACEi** |  |  |  |
| 0.63  (0.38-1.02) | 0.83  (0.52-1.31) | 0.82  (0.44-1.49) | 0.76  (0.45-1.22) | 0.78  (0.49-1.23) | 1.83  (0.35-14.2) | 0.74  (0.37-1.25) | **NA** | 0.27  (0.02-2.35) | 0.82  (0.49-1.37) | 0.61  (0.36-1.03) | **DRi+ARB** |  |  |
| 0.47  (0.18-1.23) | 0.61  (0.24-1.60) | 0.60  (0.22-1.71) | 0.56  (0.22-1.44) | 0.58  (0.22-1.52) | 1.38  (0.22-12.4) | 0.55  (0.22-1.24) | **NA** | 0.20  (0.01-2.02) | 0.61  (0.23-1.65) | 0.45  (0.16-1.32) | 0.75  (0.26-2.17) | **DRi+diuretic** |  |
| 1.11  (0.16-11.02) | 1.45  (0.21-14.4) | 1.43  (0.20-14.42) | 1.36  (0.19-13.0) | 1.36  (0.20-13.4) | 3.29  (0.33-53.1) | 1.27  (0.18-12.9) | **NA** | 0.48  (0.11-1.74) | 1.45  (0.21-14.3) | 1.09  (0.15-11.1) | 1.76  (0.24-18.1) | 2.36  (0.28-26.9) | **ARB+CCB** |

Comparisons between treatments should be read from left to right. The treatment at the top of each column is the reference group for comparisons in that column, while the comparator is the lower treatment in the step-ladder. For myocardial infarction, odds ratios (ORs) < than 1 suggests fewer outcomes with the comparator than with the reference group. Statistically significant results are in bold and underlined. Comparisons involving any renin-angiotensin receptor blocker are highlighted in light green. Comparisons of renin-angiotensin blockers against each other are highlighted in dark green.

NA = not available. Effect sizes for treatment comparisons including ACEi+diuretic were generally unstable and uninterpretable owing to small sample sizes, rare events and lack of direct evidence.

Pbo/control = placebo or control; ACEi = Angiotensin converting enzyme inhibitor; BB = beta-blocker; CCB = Calcium channel blocker; ARB = Angiotensin receptor blocker; ACEi+CCB = Angiotensin converting enzyme inhibitor + calcium channel blocker; ACEi+CCB = Angiotensin converting enzyme inhibitor + diuretic; ACEi+ARB = Angiotensin converting enzyme inhibitor + angiotensin receptor blocker; DRi+ACEi = Direct renin inhibitor (aliskiren) + angiotensin converting enzyme inhibitor; DRi+ARB = Direct renin inhibitor (aliskiren) + angiotensin receptor blocker; DRi+diuretic = Direct renin inhibitor (aliskiren) + diuretic.

**Table 8d. Stroke and all possible treatment comparisons.**

**Summary odds ratios (OR) and credible intervals from network meta-analysis.**

| **Pbo/control** |  |  |  |  |  |  |  |  |  |  |  |  |  |
| --- | --- | --- | --- | --- | --- | --- | --- | --- | --- | --- | --- | --- | --- |
| 0.90  (0.78-1.03) | **ACEi** |  |  |  |  |  |  |  |  |  |  |  |  |
| 1.03  (0.71-1.52) | 1.15  (0.80-1.66) | **BB** |  |  |  |  |  |  |  |  |  |  |  |
| 0.79  (0.66-0.96) | 0.88  (0.75-1.05) | 0.77  (0.52-1.14) | **CCB** |  |  |  |  |  |  |  |  |  |  |
| 0.91  (0.79-0.96) | 1.01  (0.88-1.16) | 0.88  (0.61-1.26) | 1.15  (0.96-1.37) | **ARB** |  |  |  |  |  |  |  |  |  |
| 0.28  (0.01-2.04) | 0.31  (0.01-2.30) | 0.27  (0.01-1.96) | 0.36  (0.01-2..61) | 0.31  (0.01-2.28) | **ACEi+CCB** |  |  |  |  |  |  |  |  |
| 0.88  (0.68-1.17) | 0.97  (0.78-1.26) | 0.85  (0.56-1.33) | 1.11  (0.87-1.41) | 0.97  (0.75-1.27) | 3.14  (0.42-88.2) | **Diuretic** |  |  |  |  |  |  |  |
| 0.95  (0.75-1.22) | 1.05  (0.81-1.41) | 0.91  (0.59-1.44) | 1.20  (0.88-1.62) | 1.04  (0.80-1.39) | 3.40  (0.48-94.6) | 1.08  (0.75-1.52) | **ACEi+diuretic** |  |  |  |  |  |  |
| 0.67  (0.17-2.61) | 0.75  (0.19-2.90) | 0.65  (0.17-2.67) | 0.84  (0.22-3.32) | 0.74  (0.19-2.90) | 2.53  (0.21-79.7) | 0.76  (0.19-3.01) | 0.70  (0.18-2.82) | **ARB+diuretic** |  |  |  |  |  |
| **0.80**  **(0.62-0.98)** | 0.88  (0.71-1.08) | 0.77  (0.51-1.14) | 1.01  (0.76-1.27) | 0.87  (0.70-1.06) | 2.79  (0.38-83.3) | 0.91  (0.64-1.21) | 0.84  (0.58-1.14) | 1.18  (0.29-4.69) | **ACEi+ARB** |  |  |  |  |
| 1.07  (0.72-1.51) | 1.19  (0.82-1.67) | 1.03  (0.63-1.68) | 1.35  (0.91-1.95) | 1.18  (0.81-1.64) | 3.79  (0.50-117) | 1.22  (0.78-1.81) | 1.13  (0.71-1.70) | 1.60  (0.39-6.37) | 1.35  (0.90-1.98) | **DRi+ACEi** |  |  |  |
| 1.15  (0.81-1.61) | 1.28  (0.91-1.78) | 1.11  (0.68-1.79) | **1.46**  **(1.01-2.06)** | 1.27  (0.91-1.75) | 4.10  (0.53-123) | 1.31  (0.88-1.91) | 1.22  (0.79-1.82) | 1.74  (0.42-6.86) | 1.44  (1.00-2.13) | 1.08  (0.75-1.58) | **DRi+ARB** |  |  |
| 0.41  (0.14-1.11) | 0.45  (0.16-1.20) | 0.40  (0.13-1.13) | 0.51  (0.18-1.37) | 0.45  (0.15-1.23) | 1.53  (0.15-45.5) | 0.46  (0.16-1.24) | 0.43  (0.14-1.17) | 0.59  (0.11-3.08) | 0.52  (0.17-1.39) | 0.38  (0.12-1.08) | 0.35  (0.12-1.01) | **DRi+diuretic** |  |
| 1.09  (0.36-3.31) | 1.21  (0.40-3.67) | 1.04  (0.33-3.33) | 1.36  (0.45-4.20) | 1.19  (0.40-3.66) | 3.81  (0.37-107) | 1.23  (0.40-3.79) | 1.13  (0.36-3.58) | 1.56  (0.79-3.26) | 1.36  (0.43-4.27) | 1.00  (0.32-3.22) | 0.93  (0.31-2.99) | 2.71  (0.62-12.44) | **ARB+CCB** |

Comparisons between treatments should be read from left to right. The treatment at the top of each column is the reference group for comparisons in that column, while the comparator is the lower treatment in the step-ladder. For stroke, odds ratios (ORs) < than 1 suggests fewer outcomes with the comparator than with the reference group. Statistically significant results are in bold and underlined. Comparisons involving any renin-angiotensin receptor blocker are highlighted in light green. Comparisons of renin-angiotensin blockers against each other are highlighted in dark green.

Pbo/control = placebo or control; ACEi = Angiotensin converting enzyme inhibitor; BB = beta-blocker; CCB = Calcium channel blocker; ARB = Angiotensin receptor blocker; ACEi+CCB = Angiotensin converting enzyme inhibitor + calcium channel blocker; ACEi+CCB = Angiotensin converting enzyme inhibitor + diuretic; ACEi+ARB = Angiotensin converting enzyme inhibitor + angiotensin receptor blocker; DRi+ACEi = Direct renin inhibitor (aliskiren) + angiotensin converting enzyme inhibitor; DRi+ARB = Direct renin inhibitor (aliskiren) + angiotensin receptor blocker; DRi+diuretic = Direct renin inhibitor (aliskiren) + diuretic.

**Table 8e. Progression of renal disease (composite outcome) and all possible treatment comparisons.**

**Summary odds ratios (OR) and credible intervals from network meta-analysis.**

| **Pbo/control** |  |  |  |  |  |  |  |  |  |  |  |  |  |
| --- | --- | --- | --- | --- | --- | --- | --- | --- | --- | --- | --- | --- | --- |
| 0.92  (0.73-1.14) | **ACEi** |  |  |  |  |  |  |  |  |  |  |  |  |
| **9.00**  **(2.15-41.83)** | **9.80 (3.37-45.05)** | **BB** |  |  |  |  |  |  |  |  |  |  |  |
| 1.08  (0.81-1.50) | 1.17  (0.90-1.64) | **0.12**  **(0.03-0.50)** | **CCB** |  |  |  |  |  |  |  |  |  |  |
| 1.01  (0.84-1.24) | 1.10  (0.90-1.40) | **0.11**  **(0.02-0.47)** | 0.93  (0.69-1.24) | **ARB** |  |  |  |  |  |  |  |  |  |
| NA | NA | NA | NA | NA | **ACEi+CCB** |  |  |  |  |  |  |  |  |
| 1.05  (0.69-1.63) | 1.14  (0.78-1.74) | **0.12**  **(0.02-0.50)** | 0.98  (0.64-1.43) | 1.04  (0.67-1.59) | NA | **Diuretic** |  |  |  |  |  |  |  |
| 0.90  (0.58-1.39) | 0.98  (0.61-1.62) | **0.10**  **(0.02-0.44)** | 0.83  (0.48-1.39) | 0.89  (0.54-1.42) | NA | 0.85  (0.46-1.57) | **ACEi+diuretic** |  |  |  |  |  |  |
| NA | NA | NA | NA | NA | NA | NA | NA | **ARB+diuretic** |  |  |  |  |  |
| 0.90  (0.65-1.19) | 0.97  (0.72-1.29) | **0.10**  **(0.02-0.42)** | 0.83  (0.54-1.17) | 0.89  (0.66-1.13) | NA | 0.85  (0.51-1.34) | 1.00  (0.57-1.66) | NA | **ACEi+ARB** |  |  |  |  |
| 0.91  (0.58-1.45) | 0.99  (0.65-1.57) | **0.10**  **(0.02-0.45)** | 0.84  (0.50-1.38) | 0.90  (0.58-1.39) | NA | 0.86  (0.48-1.56) | 1.01  (0.54-1.92) | NA | 1.02  (0.63-1.71) | **DRi+ACEi** |  |  |  |
| 1.08  (0.69-1.70) | 1.18  (0.78-1.84) | **0.12**  **(0.02-0.53)** | 1.00  (0.60-1.62) | 1.07  (0.70-1.63) | NA | 1.03  (0.58-1.84) | 1.20  (0.65-2.27) | NA | 1.21  (0.76-2.02) | 1.19  (0.73-1.92) | **DRi+ARB** |  |  |
| NA | NA | NA | NA | NA | NA | NA | NA | NA | NA | NA | NA | **DRi+diuretic** |  |
| NA | NA | NA | NA | NA | NA | NA | NA | 0.99 (0.33-2.98) | NA | NA | **NA** | NA | **ARB+CCB** |

Comparisons between treatments should be read from left to right. The treatment at the top of each column is the reference group for comparisons in that column, while the comparator is the lower treatment in the step-ladder. For progression of renal disease, odds ratios (ORs) < than 1 suggests fewer outcomes with the comparator than with the reference group. Statistically significant results are in bold and underlined. Comparisons involving any renin-angiotensin receptor blocker are highlighted in light green. Comparisons of renin-angiotensin blockers against each other are highlighted in dark green.

NA = not available. Effect sizes for treatment comparisons including ARB+diuretic and/or ARB+CCB were generally unstable and uninterpretable owing to small sample sizes, rare events and lack of direct evidence. There were no studies evaluating ACEi+CCB and DRi+diuretic.

Pbo/control = placebo or control; ACEi = Angiotensin converting enzyme inhibitor; BB = beta-blocker; CCB = Calcium channel blocker; ARB = Angiotensin receptor blocker; ACEi+CCB = Angiotensin converting enzyme inhibitor + calcium channel blocker; ACEi+CCB = Angiotensin converting enzyme inhibitor + diuretic; ACEi+ARB = Angiotensin converting enzyme inhibitor + angiotensin receptor blocker; DRi+ACEi = Direct renin inhibitor (aliskiren) + angiotensin converting enzyme inhibitor; DRi+ARB = Direct renin inhibitor (aliskiren) + angiotensin receptor blocker; DRi+diuretic = Direct renin inhibitor (aliskiren) + diuretic.

**Table 8f. End-stage renal disease and all possible treatment comparisons.**

**Summary odds ratios (OR) and credible intervals from network meta-analysis.**

| **Pbo/control** |  |  |  |  |  |  |  |  |  |  |  |  |  |
| --- | --- | --- | --- | --- | --- | --- | --- | --- | --- | --- | --- | --- | --- |
| **0.68**  **(0.51-0.91)** | **ACEi** |  |  |  |  |  |  |  |  |  |  |  |  |
| 3.34  (0.78-18.56) | **4.88 (1.17-26.34)** | **BB** |  |  |  |  |  |  |  |  |  |  |  |
| 0.87  (0.61-1.21) | 1.27  (0.91-1.75) | 0.26  (0.05-1.11) | **CCB** |  |  |  |  |  |  |  |  |  |  |
| **0.74**  **(0.57-0.97)** | 1.09  (0.84-1.42) | **0.22**  **(0.04-0.94)** | 0.85  (0.63-1.18) | **ARB** |  |  |  |  |  |  |  |  |  |
| NA | NA | NA | NA | NA | **ACEi+CCB** |  |  |  |  |  |  |  |  |
| 0.65  (0.38-1.07) | 0.95  (0.59-1.50) | **0.19**  **(0.03-0.86)** | 0.74  (0.47-1.19) | 0.87  (0.53-1.42) | NA | **Diuretic** |  |  |  |  |  |  |  |
| 1.20  (0.59-2.47) | 1.75  (0.81-3.80) | 0.36  (0.06-1.84) | 1.37  (0.63-3.09) | 1.61  (0.75-3.47) | NA | 1.84  (0.78-4.49) | **ACEi+diuretic** |  |  |  |  |  |  |
| NA | NA | NA | NA | NA | NA | NA | NA | **ARB+diuretic** |  |  |  |  |  |
| **0.62**  **(0.42-0.90)** | 0.91  (0.63-1.27) | **0.19**  **(0.03-0.80)** | 0.72  (0.47-1.08) | 0.84  (0.59-1.14) | NA | 0.97  (0.54-1.66) | 0.52  (0.23-1.15) | **NA** | **ACEi+ARB** |  |  |  |  |
| 0.74  (0.43-1.31) | 1.09  (0.66-1.85) | 0.22  (0.04-1.03) | 0.85  (0.49-1.55) | 1.00  (0.61-1.69) | NA | 1.15  (0.59-2.31) | 0.62  (0.25-1.55) | **NA** | 1.19  (0.68-2.21) | **DRi+ACEi** |  |  |  |
| 0.79  (0.47-1.37) | 1.17  (0.72-1.93) | 0.24  (0.04-1.09) | 0.92  (0.53-1.62) | 1.07  (0.66-1.76) | NA | 1.23  (0.64-2.42) | 0.66  (0.27-1.65) | **NA** | 1.27  (0.74-2.32) | 1.07  (0.61-1.86) | **DRi+ARB** |  |  |
| NA | NA | NA | NA | NA | NA | NA | NA | NA | NA | NA | NA | **DRi+diuretic** |  |
| NA | NA | NA | NA | NA | NA | NA | NA | **NA** | NA | **NA** | **NA** | NA | **ARB+CCB** |

Comparisons between treatments should be read from left to right. The treatment at the top of each column is the reference group for comparisons in that column, while the comparator is the lower treatment in the step-ladder. For end-stage renal disease, odds ratios (ORs) < than 1 suggests fewer outcomes with the comparator than with the reference group. Statistically significant results are in bold and underlined. Comparisons involving any renin-angiotensin receptor blocker are highlighted in light green. Comparisons of renin-angiotensin blockers against each other are highlighted in dark green.

NA = not available. Effect sizes for treatment comparisons including ARB+diuretic and/or ARB+CCB were generally unstable and uninterpretable owing to small sample sizes, rare events and lack of direct evidence. There were no studies evaluating ACEi+CCB and DRi+diuretic.

Pbo/control = placebo or control; ACEi = Angiotensin converting enzyme inhibitor; BB = beta-blocker; CCB = Calcium channel blocker; ARB = Angiotensin receptor blocker; ACEi+CCB = Angiotensin converting enzyme inhibitor + calcium channel blocker; ACEi+CCB = Angiotensin converting enzyme inhibitor + diuretic; ACEi+ARB = Angiotensin converting enzyme inhibitor + angiotensin receptor blocker; DRi+ACEi = Direct renin inhibitor (aliskiren) + angiotensin converting enzyme inhibitor; DRi+ARB = Direct renin inhibitor (aliskiren) + angiotensin receptor blocker; DRi+diuretic = Direct renin inhibitor (aliskiren) + diuretic.

**Table 8g. Doubling of serum creatinine and all possible treatment comparisons.**

**Summary odds ratios (OR) and credible intervals from network meta-analysis.**

| **Pbo/control** |  |  |  |  |  |  |  |  |  |  |  |  |  |
| --- | --- | --- | --- | --- | --- | --- | --- | --- | --- | --- | --- | --- | --- |
| **0.70**  **(0.52-0.91)** | **ACEi** |  |  |  |  |  |  |  |  |  |  |  |  |
| 4.52  (0.84-27.15) | **6.49 (1.22-38.88)** | **BB** |  |  |  |  |  |  |  |  |  |  |  |
| 0.98  (0.65-1.49) | 1.40  (0.95-2.19) | 0.22  (0.04-1.15) | **CCB** |  |  |  |  |  |  |  |  |  |  |
| 0.88  (0.70-1.17) | 1.26  (0.97-1.79) | 0.20  (0.03-1.06) | 0.90  (0.61-1.37) | **ARB** |  |  |  |  |  |  |  |  |  |
| NA | NA | NA | NA | NA | **ACEi+CCB** |  |  |  |  |  |  |  |  |
| 0.82  (0.43-1.52) | 1.17  (0.65-2.15) | 0.18  (0.03-1.04) | 0.84  (0.45-1.49) | 0.93  (0.47-1.68) | NA | **Diuretic** |  |  |  |  |  |  |  |
| 1.22  (0.61-2.49) | 1.75  (0.84-3.84) | 0.27  (0.04-1.65) | 1.25  (0.55-2.81) | 1.39  (0.64-2.89) | NA | 1.50  (0.59-3.89) | **ACEi+diuretic** |  |  |  |  |  |  |
| NA | NA | NA | NA | NA | NA | NA | NA | **ARB+diuretic** |  |  |  |  |  |
| 0.70  (0.43-1.09) | 0.99  (0.65-1.56) | **0.15**  **(0.02-0.85)** | 0.71  (0.40-1.22) | 0.79  (0.48-1.19) | NA | 0.85  (0.41-1.73) | 0.57  (0.24-1.30) | NA | **ACEi+ARB** |  |  |  |  |
| 0.65  (0.36-1.21) | 0.93  (0.53-1.74) | 0.14  (0.02-0.84) | 0.67  (0.34-1.32) | 0.74  (0.40-1.31) | NA | 0.80  (0.36-1.85) | 0.54  (0.21-1.36) | NA | 0.94  (0.47-1.96) | **DRi+ACEi** |  |  |  |
| 0.89  (0.49-1.64) | 1.27  (0.73-2.35) | 0.20  (0.03-1.15) | 0.91  (0.46-1.79) | 1.01  (0.55-1.76) | NA | 1.09  (0.48-2.51) | 0.73  (0.29-1.84) | NA | 1.28  (0.65-2.63) | 1.36  (0.71-2.62) | **DRi+ARB** |  |  |
| NA | NA | NA | NA | NA | NA | NA | NA | NA | NA | NA | NA | **ARB+CCB** |  |
| 1.26  (0.48-3.23) | 1.81  (0.72-4.62) | 0.28  (0.04-1.84) | 1.29  (0.50-3.20) | 1.43  (0.53-3.57) | NA | 1.54  (0.76-3.14) | 1.03  (0.31-3.31) | 1.34  (0.39-4.74) | 1.81  (0.66-5.04) | 1.93  (0.64-5.64) | 1.41  (0.47-4.13**)** | **NA** | **DRi+diuretic** |

Comparisons between treatments should be read from left to right. The treatment at the top of each column is the reference group for comparisons in that column, while the comparator is the lower treatment in the step-ladder. For doubling serum creatinine, odds ratios (ORs) < than 1 suggests fewer outcomes with the comparator than with the reference group. Statistically significant results are in bold and underlined. Comparisons involving any renin-angiotensin receptor blocker are highlighted in light green. Comparisons of renin-angiotensin blockers against each other are highlighted in dark green.

NA = not available. Effect sizes for treatment comparisons including ARB+diuretic and/or ARB+CCB were generally unstable and uninterpretable owing to small sample sizes, rare events and lack of direct evidence. There were no studies evaluating ACEi+CCB.

Pbo/control = placebo or control; ACEi = Angiotensin converting enzyme inhibitor; BB = beta-blocker; CCB = Calcium channel blocker; ARB = Angiotensin receptor blocker; ACEi+CCB = Angiotensin converting enzyme inhibitor + calcium channel blocker; ACEi+CCB = Angiotensin converting enzyme inhibitor + diuretic; ACEi+ARB = Angiotensin converting enzyme inhibitor + angiotensin receptor blocker; DRi+ACEi = Direct renin inhibitor (aliskiren) + angiotensin converting enzyme inhibitor; DRi+ARB = Direct renin inhibitor (aliskiren) + angiotensin receptor blocker; DRi+diuretic = Direct renin inhibitor (aliskiren) + diuretic.

**Table 8h. All-cause mortality and all possible treatment comparisons.**

**Summary odds ratios (OR) and credible intervals from network meta-analysis.**

| **Pbo/control** |  |  |  |  |  |  |  |  |  |  |  |  |  |
| --- | --- | --- | --- | --- | --- | --- | --- | --- | --- | --- | --- | --- | --- |
| 0.96  (0.84-1.08) | **ACEi** |  |  |  |  |  |  |  |  |  |  |  |  |
| 1.32  (0.96-1.81) | **1.38**  **(1.02-1.88)** | **BB** |  |  |  |  |  |  |  |  |  |  |  |
| 0.93  (0.79-1.10) | 0.98  (0.84-1.14) | **0.71**  **(0.51-0.98)** | **CCB** |  |  |  |  |  |  |  |  |  |  |
| 0.99  (0.88-1.12) | 1.04  (0.92-1.18) | 0.75  (0.56-1.02) | 1.06  (0.91-1.24) | **ARB** |  |  |  |  |  |  |  |  |  |
| 0.44  (0.13-1.09) | 0.47  (0.13-1.14) | **0.34**  **(0.09-0.87)** | 0.48  (0.14-1.18) | 0.45  (0.13-1.11) | **ACEi+CCB** |  |  |  |  |  |  |  |  |
| 1.00  (0.76-1.30) | 1.04  (0.81-1.34) | 0.76  (0.51-1.11) | 1.07  (0.83-1.38) | 1.00  (0.77-1.31) | 2.24  (0.89-7.83) | **Diuretic** |  |  |  |  |  |  |  |
| 0.87  (0.65-1.16) | 0.90  (0.67-1.26) | 0.66  (0.43-1.02) | 0.93  (0.67-1.31) | 0.87  (0.64-1.20) | 1.96  (0.77-6.78) | 0.87  (0.59-1.30) | **ACEi+diuretic** |  |  |  |  |  |  |
| 1.14  (0.34-3.79) | 1.19  (0.35-3.97) | 0.86  (0.25-3.00) | 1.22  (0.36-4.06) | 1.15  (0.34-3.81) | 2.61 (0.56-12.93) | 1.14  (0.33-3.88) | 1.31  (0.37-4.52) | **ARB+diuretic** |  |  |  |  |  |
| 0.99  (0.82-1.18) | 1.03  (0.88-1.21) | 0.75  (0.54-1.04) | 1.06  (0.86-1.30) | 1.00  (0.84-1.17) | 2.22  (0.89-7.79) | 0.99  (0.74-1.32) | 1.14  (0.80-1.59) | 0.87  (0.26-2.94) | **ACEi+ARB** |  |  |  |  |
| 1.23  (0.90-1.66) | 1.29  (0.96-1.72) | 0.93  (0.61-1.40) | 1.32  (0.96-1.80) | 1.24  (0.92-1.65) | **2.77**  **(1.08-9.86)** | 1.23  (0.84-1.79) | 1.42  (0.92-2.14) | 1.08  (0.31-3.74) | 1.24  (0.90-1.72) | **DRi+ACEi** |  |  |  |
| 0.86  (0.63-1.17) | 0.90  (0.67-1.21) | **0.66**  **(0.43-0.99)** | 0.92  (0.67-1.27) | 0.87  (0.64-1.16) | 1.94  (0.76-6.92) | 0.87  (0.59-1.26) | 1.00  (0.65-1.51) | 0.76  (0.22-2.64) | 0.87  (0.63-1.21) | **0.70**  **(0.51-0.98)** | **DRi+ARB** |  |  |
| 1.46  (0.86-2.48) | 1.53  (0.91-2.76) | 1.11  (0.61-2.01) | 1.56  (0.93-2.64) | 1.47  (0.87-2.49) | **3.30 (1.17-12.36)** | 1.47  (0.93-2.32) | 1.69  (0.91-3.08) | 1.29  (0.35-4.79) | 1.48  (0.86-2.55) | 1.19 (0.66-2.15) | 1.69  (0.94-3.06) | **DRi+diuretic** |  |
| 0.84  (0.29-2.36) | 0.88  (0.31-2.47) | 0.64  (0.22-1.88) | 0.90  (0.32-2.53) | 0.85  (0.30-2.37) | 1.93  (0.48-8.77) | 0.85  (0.29-2.42) | 0.97  (0.33-2.85) | 0.74  (0.39-1.36) | 0.86  (0.30-2.41) | 0.69  (0.23-1.98) | 0.98  (0.33-2.83) | 0.57  (0.18-1.82) | **ARB+CCB** |

Comparisons between treatments should be read from left to right. The treatment at the top of each column is the reference group for comparisons in that column, while the comparator is the lower treatment in the step-ladder. For all-cause mortality, odds ratios (ORs) < than 1 suggests fewer outcomes with the comparator than with the reference group. Statistically significant results are in bold and underlined. Comparisons involving any renin-angiotensin receptor blocker are highlighted in light green. Comparisons of renin-angiotensin blockers against each other are highlighted in dark green.

Pbo/control = placebo or control; ACEi = Angiotensin converting enzyme inhibitor; BB = beta-blocker; CCB = Calcium channel blocker; ARB = Angiotensin receptor blocker; ACEi+CCB = Angiotensin converting enzyme inhibitor + calcium channel blocker; ACEi+CCB = Angiotensin converting enzyme inhibitor + diuretic; ACEi+ARB = Angiotensin converting enzyme inhibitor + angiotensin receptor blocker; DRi+ACEi = Direct renin inhibitor (aliskiren) + angiotensin converting enzyme inhibitor; DRi+ARB = Direct renin inhibitor (aliskiren) + angiotensin receptor blocker; DRi+diuretic = Direct renin inhibitor (aliskiren) + diuretic.

**Table 8i. Angina pectoris and all possible treatment comparisons.**

**Summary odds ratios (OR) and credible intervals from network meta-analysis.**

| **Pbo/control** |  |  |  |  |  |  |  |  |  |  |  |  |  |
| --- | --- | --- | --- | --- | --- | --- | --- | --- | --- | --- | --- | --- | --- |
| **0.81**  **(0.65-0.96)** | **ACEi** |  |  |  |  |  |  |  |  |  |  |  |  |
| 0.99  (0.62-1.57) | 1.22  (0.78-1.94) | **BB** |  |  |  |  |  |  |  |  |  |  |  |
| **0.76**  **(0.61-0.98)** | 0.94  (0.78-1.22) | 0.77  (0.48-1.26) | **CCB** |  |  |  |  |  |  |  |  |  |  |
| 0.92  (0.77-1.09) | 1.14  (0.98-1.37) | 0.93  (0.59-1.45) | 1.21  (0.96-1.47) | **ARB** |  |  |  |  |  |  |  |  |  |
| NA | NA | NA | NA | NA | **ACEi+CCB** |  |  |  |  |  |  |  |  |
| 0.78  (0.56-1.09) | 0.96  (0.74-1.34) | 0.79  (0.47-1.35) | 1.02  (0.76-1.36) | 0.84  (0.63-1.18) | **NA** | **Diuretic** |  |  |  |  |  |  |  |
| NA | NA | NA | NA | NA | NA | NA | **ACEi+diuretic** |  |  |  |  |  |  |
| 7.90  (0.22-452) | 9.81  (0.27-564) | 8.09  (0.21-470) | 10.3  (0.28-598) | 8.61  (0.24-492) | NA | 10.07  (0.28-591.20) | NA | **ARB+diuretic** |  |  |  |  |  |
| 0.81  (0.62-1.02) | 1.00  (0.82-1.23) | 0.82  (0.50-1.32) | 1.07  (0.78-1.36) | 0.88  (0.70-1.08) | NA | 1.05  (0.71-1.44) | NA | 0.10  (0.00-3.78) | **ACEi+ARB** |  |  |  |  |
| NA | NA | NA | NA | NA | NA | NA | NA | NA | NA | **DRi+ACEi** |  |  |  |
| NA | NA | NA | NA | NA | NA | NA | NA | NA | NA | NA | **DRi+ARB** |  |  |
| 1.07  (0.44-2.57) | 1.32  (0.56-3.17) | 1.08  (0.41-2.85) | 1.40  (0.59-3.31) | 1.16  (0.49-2.78) | NA | 1.37  (0.61-3.09) | NA | 0.14  (0.00-5.59) | 1.32  (0.55-3.22) | **NA** | **NA** | **DRi+diuretic** |  |
| 2.59  (0.22-75.83) | 3.25 (0.28-89.45) | 2.68  (0.22-77.81) | 3.40 (0.29-97.62) | 2.84 (0.24-78.51) | NA | 3.36  (0.28-100.90) | NA | 0.40  (0.01-4.27) | 3.23  (0.27-91.01) | **NA** | **NA** | 2.43  (0.18-74.47) | **ARB+CCB** |

Comparisons between treatments should be read from left to right. The treatment at the top of each column is the reference group for comparisons in that column, while the comparator is the lower treatment in the step-ladder. For angina pectoris, odds ratios (ORs) < than 1 suggests fewer outcomes with the comparator than with the reference group. Statistically significant results are in bold and underlined. Comparisons involving any renin-angiotensin receptor blocker are highlighted in light green. Comparisons of renin-angiotensin blockers against each other are highlighted in dark green.

NA = not available. There were no studies evaluating ACEi+CCB, ACEi+diuretic, DRi+diuretic, DRi+ACEi and DRi+ARB.

Pbo/control = placebo or control; ACEi = Angiotensin converting enzyme inhibitor; BB = beta-blocker; CCB = Calcium channel blocker; ARB = Angiotensin receptor blocker; ACEi+CCB = Angiotensin converting enzyme inhibitor + calcium channel blocker; ACEi+CCB = Angiotensin converting enzyme inhibitor + diuretic; ACEi+ARB = Angiotensin converting enzyme inhibitor + angiotensin receptor blocker; DRi+ACEi = Direct renin inhibitor (aliskiren) + angiotensin converting enzyme inhibitor; DRi+ARB = Direct renin inhibitor (aliskiren) + angiotensin receptor blocker; DRi+diuretic = Direct renin inhibitor (aliskiren) + diuretic.

**Table 8j. Heart failure and all possible treatment comparisons.**

**Summary odds ratios (OR) and credible intervals from network meta-analysis.**

| **Pbo/control** |  |  |  |  |  |  |  |  |  |  |  |  |  |
| --- | --- | --- | --- | --- | --- | --- | --- | --- | --- | --- | --- | --- | --- |
| 0.86  (0.73-1.01) | **ACEi** |  |  |  |  |  |  |  |  |  |  |  |  |
| 1.25  (0.78-2.00) | 1.45  (0.92-2.27) | **BB** |  |  |  |  |  |  |  |  |  |  |  |
| 1.11  (0.90-1.36) | **1.29**  **(1.08-1.54)** | 0.89  (0.55-1.44) | **CCB** |  |  |  |  |  |  |  |  |  |  |
| **0.86**  **(0.72-0.99)** | 0.99  (0.86-1.12) | 0.69  (0.44-1.06) | **0.77**  **(0.63-0.92)** | **ARB** |  |  |  |  |  |  |  |  |  |
| NA | NA | NA | NA | NA | **ACEi+CCB** |  |  |  |  |  |  |  |  |
| 0.81  (0.60-1.09) | 0.94  (0.72-1.23) | 0.65  (0.39-1.11) | **0.73**  **(0.56-0.96)** | 0.94  (0.72-1.28) | NA | **Diuretic** |  |  |  |  |  |  |  |
| 1.73  (0.19-19.48) | 2.02 (0.22-22.89) | 1.38  (0.14-16.51) | 1.57 (0.17-17.55) | 2.03 (0.22-22.70) | NA | 2.15  (0.23-24.53) | **ACEi+diuretic** |  |  |  |  |  |  |
| 0.32  (0.04-2.09) | 0.37  (0.05-2.43) | 0.26  (0.03-1.75) | 0.29  (0.04-1.90) | 0.38  (0.05-2.44) | NA | 0.40  (0.05-2.62) | 0.18  (0.01-3.53) | **ARB+diuretic** |  |  |  |  |  |
| **0.75**  **(0.60-0.91)** | 0.86  (0.73-1.01) | **0.60**  **(0.37-0.96)** | **0.67**  **(0.53-0.83)** | 0.87  (0.74-1.03) | NA | 0.92  (0.67-1.24) | 0.43  (0.04-3.99) | 2.32  (0.35-17.13) | **ACEi+ARB** |  |  |  |  |
| 0.91  (0.63-1.28) | 1.06  (0.75-1.45) | 0.73  (0.43-1.27) | 0.82  (0.57-1.16) | 1.07  (0.77-1.48) | NA | 1.13  (0.74-1.69) | 0.52  (0.04-4.95) | 2.85  (0.42-21.64) | 1.23  (0.86-1.74) | **DRi+ACEi** |  |  |  |
| 0.71  (0.50-1.00) | 0.83  (0.59-1.13) | **0.57**  **(0.33-0.99)** | **0.64**  **(0.45-0.91)** | 0.83  (0.60-1.16) | NA | 0.88  (0.58-1.32) | 0.41  (0.04-3.93) | 2.23  (0.33-16.94) | 0.96  (0.67-1.36) | 0.78  (0.54-1.14) | **DRi+ARB** |  |  |
| 0.47  (0.09-1.91) | 0.55  (0.10-2.23) | 0.38  (0.07-1.66) | 0.43  (0.08-1.74) | 0.56  (0.10-2.24) | NA | 0.59  (0.11-2.42) | 0.27  (0.02-3.82) | 1.46  (0.45-5.39) | 0.64  (0.12-2.59) | 0.52  (0.10-2.19) | 0.67  (0.12-2.81) | **ARB+CCB** |  |
| 0.90  (0.55-1.49) | 1.05  (0.65-1.70) | 0.72  (0.38-1.41) | 0.81  (0.50-1.33) | 1.06  (0.65-1.76) | NA | 1.11  (0.74-1.67) | 0.52  (0.04-5.07) | 2.82  (0.40-21.72) | 1.21  (0.73-2.03) | 0.99  (0.56-1.78) | 1.26  (0.72-2.27) | 1.90  (0.43-10.61) | **DRi+diuretic** |

Comparisons between treatments should be read from left to right. The treatment at the top of each column is the reference group for comparisons in that column, while the comparator is the lower treatment in the step-ladder. For heart failure, odds ratios (ORs) < than 1 suggests fewer outcomes with the comparator than with the reference group. Statistically significant results are in bold and underlined. Comparisons involving any renin-angiotensin receptor blocker are highlighted in light green. Comparisons of renin-angiotensin blockers against each other are highlighted in dark green.

NA = not available. There were no studies evaluating ACEi+CCB.

Pbo/control = placebo or control; ACEi = Angiotensin converting enzyme inhibitor; BB = beta-blocker; CCB = Calcium channel blocker; ARB = Angiotensin receptor blocker; ACEi+CCB = Angiotensin converting enzyme inhibitor + calcium channel blocker; ACEi+CCB = Angiotensin converting enzyme inhibitor + diuretic; ACEi+ARB = Angiotensin converting enzyme inhibitor + angiotensin receptor blocker; DRi+ACEi = Direct renin inhibitor (aliskiren) + angiotensin converting enzyme inhibitor; DRi+ARB = Direct renin inhibitor (aliskiren) + angiotensin receptor blocker; DRi+diuretic = Direct renin inhibitor (aliskiren) + diuretic.
