# Supplementary material for: Cardiovascular and Renal Outcomes of Renin–Angiotensin System Blockade in Adult Patients with Diabetes Mellitus: A Systematic Review with Network Meta-Analyses
Source: PLoS Med. 2016 Mar 8;13(3):e1001971. doi: 10.1371/journal.pmed.1001971 (PMC4783064; doi:10.1371/journal.pmed.1001971)
Supplement: S9 Table — (DOCX) [file pmed.1001971.s012.docx]

**Table S9. Summary of SUCRA with 95% credible intervals by outcome and treatment.**

|  | **Major CV outcome** | **CV mortality** | **Myocardial infarction** | **Stroke** | **Progression of renal disease** | **ESRD** | **Doubling CrS** | **All-cause mortality** | **Angina pectoris** | **Heart failure** |
| --- | --- | --- | --- | --- | --- | --- | --- | --- | --- | --- |
| **ACEi** | 0.54 (0.31-0.85) | 0.54 (0.23-0.85) | 0.61 (0.38-0.85) | 0.46 (0.23-0.77) | 0.64 (0.27-1.00) | 0.73 (0.45-0.91) | 0.75 (0.50-1.00) | 0.61 (0.31-0.85) | 0.67 (0.33-1.00) | 0.50 (0.25-0.75) |
| **BB** | 0.15 (0.00-0.69) | 0.23 (0.00-0.77) | 0.61 (0.15-0.92) | 0.23 (0.00-0.85) | 0.00 (0.00-0.18) | 0.09 (0.00-0.45) | 0.00 (0.00-0.58) | 0.15 (0.00-0.54) | 0.33 (0.00-1.00) | 0.08 (0.00-0.67) |
| **CCB** | 0.69 (0.38-0.92) | 0.61 (0.23-0.92) | 0.46 (0.23-0.85) | 0.69 (0.38-0.92) | 0.36 (0.09-0.82) | 0.36 (0.18-0.73) | 0.42 (0.08-0.83) | 0.69 (0.31-0.92) | 0.89 (0.44-1.00) | 0.17 (0.00-0.42) |
| **ARB** | 0.54 (0.23-0.77) | 0.39 (0.15-0.69) | 0.54 (0.23-0.77) | 0.46 (0.23-0.77) | 0.45 (0.18-0.82) | 0.64 (0.36-0.91) | 0.50 (0.25-0.83) | 0.46 (0.23-0.77) | 0.44 (0.22-0.78) | 0.50 (0.25-0.75) |
| **ACEi+CCB** | 0.85 (0.15-1.00) | 1.00 (0.77-1.00) | 1.00 (0.15-1.00) | 1.00 (0.00-1.00) | NA | NA | NA | 1.00 (0.31-1.00) | NA | NA |
| **Diuretic** | 0.46 (0.08-0.85) | 0.62 (0.08-0.92) | 0.46 (0.08-0.85) | 0.54 (0.08-0.85) | 0.36 (0.09-1.00) | 0.82 (0.27-1.00) | 0.58 (0.17-1.00) | 0.46 (0.15-0.92) | 0.78 (0.33-1.00) | 0.67 (0.25-0.92) |
| **ACEi+diuretic** | 0.46 (0.00-0.92) | 0.77 (0.08-0.92) | 0.00 (0.00-0.23) | 0.38 (0.08-0.85) | 0.45 (0.18-0.82) | 0.18 (0.09-0.91) | 0.25 (0.00-0.83) | 0.77 (0.15-1.00) | NA | 0.00 (0.00-1.00) |
| **ARB+diuretic** | 0.92 (0.00-1.00) | 0.85 (0.00-1.00) | 0.08 (0.00-0.92) | 0.85 (0.00-1.00) | 0.36 (0.09-1.00) | 0.00 (0.00-0.91) | 0.92 (0.00-1.00) | 0.23 (0.00-1.00) | 0.00 (0.00-1.00) | 1.00 (0.00-1.00) |
| **ACEi+ARB** | 0.62 (0.23-0.92) | 0.46 (0.08-0.85) | 0.61 (0.23-0.92) | 0.69 (0.31-0.92) | 0.73 (0.18-1.00) | 0.82 (0.45-1.00) | 0.75 (0.33-1.00) | 0.46 (0.15-0.85) | 0.67 (0.33-1.00) | 0.75 (0.50-1.00) |
| **DRi+ACEi** | 0.15 (0.00-0.62) | 0.08 (0.00-0.69) | 0.23 (0.08-0.77) | 0.15 (0.00-0.77) | 0.73 (0.09-1.00) | 0.64 (0.18-1.00) | 0.83 (0.25-1.00) | 0.15 (0.00-0.69) | NA | 0.42 (0.08-0.83) |
| **DRi+ARB** | 0.54 (0.08-0.92) | 0.61 (0.08-0.92) | 0.85 (0.31-1.00) | 0.15 (0.00-0.61) | 0.36 (0.09-0.91) | 0.45 (0.18-0.91) | 0.50 (0.08-0.92) | 0.77 (0.23-1.00) | NA | 0.83 (0.33-1.00) |
| **DRi+diuretic** | 0.38 (0.00-0.92) | 0.15 (0.00-0.85) | 0.92 (0.23-1.00) | 0.92 (0.15-1.00) | NA | NA | 0.67 (0.00-1.00) | 0.08 (0.00-0.77) | 0.22 (0.00-1.00) | 0.42 (0.00-0.92) |
| **ARB+CCB** | 0.92 (0.00-1.00) | 0.92 (0.00-1.00) | 0.23 (0.08-1.00) | 0.15 (0.00-0.92) | 0.91 (0.00-1.00) | 1.00 (0.09-1.00) | 0.25 (0.00-0.92) | 0.85 (0.08-1.00) | 0.11 (0.00-1.00) | 0.92 (0.00-1.00) |
| **Pblo/control** | 0.31 (0.07-0.54) | 0.38 (0.08-0.77) | 0.23 (0.08-0.54) | 0.31 (0.08-0.54) | 0.45 (0.18-0.91) | 0.27 (0.09-0.55) | 0.42 (0.17-0.67) | 0.46 (0.15-0.77) | 0.33 (0.11-0.56) | 0.25 (0.08-0.50) |

SUCRA = surface under the cumulative ranking (higher values are better while wider credible intervals show increased uncertainty). NA = not available.

Values for renin-angiotensin blockers (alone or in combination) are highlighted in dark green.

ACEi = Angiotensin converting enzyme inhibitor; BB = beta-blocker; CCB = Calcium channel blocker; ARB = Angiotensin receptor blocker; ACEi+CCB = Angiotensin converting enzyme inhibitor + calcium channel blocker; ACEi+CCB = Angiotensin converting enzyme inhibitor + diuretic; ACEi+ARB = Angiotensin converting enzyme inhibitor + angiotensin receptor blocker; DRi+ACEi = Direct renin inhibitor (aliskiren) + angiotensin converting enzyme inhibitor; DRi+ARB = Direct renin inhibitor (aliskiren) + angiotensin receptor blocker; DRi+diuretic = Direct renin inhibitor (aliskiren) + diuretic.
