# Supplementary material for: Cardiovascular and Renal Outcomes of Renin–Angiotensin System Blockade in Adult Patients with Diabetes Mellitus: A Systematic Review with Network Meta-Analyses
Source: PLoS Med. 2016 Mar 8;13(3):e1001971. doi: 10.1371/journal.pmed.1001971 (PMC4783064; doi:10.1371/journal.pmed.1001971)
Supplement: S10 Table — (DOCX) [file pmed.1001971.s013.docx]

**S10 Table. Sensitivity analyses.**

**Table 10a. Sensitivity analyses. Major cardiovascular outcome.**

|  | **Base case model** | **Exclusion of high RoB studies** | **Exclusion of small studies (<100 patients)** | **Exclusion of type 1 DM studies** | **Exclusion of normotension studies** | **Adjusted for publication year** | **Adjusted for mean age** | **Adjusted for % males** | **Adjusted for control group risk** | **Rate ratios based on patient-years** |
| --- | --- | --- | --- | --- | --- | --- | --- | --- | --- | --- |
| **ACEi** | reference | reference | reference | reference | reference | reference | reference | reference | reference | reference |
| **BB** | 1.29 (0.95-1.76) | 1.29 (0.92-1.83) | 1.29 (0.94-1.75) | 1.29 (0.94-1.77) | 1.29 (0.94-1.77) | 1.27 (0.94-1.73) | 1.29 (0.94-1.77) | 1.29 (0.94-1.79) | 1.29 (0.94-1.76) | 1.22 (0.94-1.59) |
| **CCB** | 0.93 (0.79-1.12) | 0.93 (0.78-1.16) | 0.92 (0.78-1-09) | 0.92 (0.78-1.10) | 0.92 (0.78-1.10) | 0.93 (0.79-1.11) | 0.93 (0.79-1.12) | 0.93 (0.78-1.13) | 0.94 (0.79-1.13) | 0.94 (0.82-1.09) |
| **ARB** | 1.02 (0.90-1.18) | 1.03 (0.89-1.21) | 1.02 (0.90-1.17) | 1.02 (0.90-1.18) | 1.02 (0.90-1.18) | 1.00 (0.89-1.16) | 1.03 (0.91-1.19) | 1.03 (0.90-1.19) | 1.02 (0.90-1.18) | 1.01 (0.92-1.14) |
| **ACEi+CCB** | 0.47 (0.12-1.41) | NA | 0.46 (0.11-1.38) | 0.47 (0.12-1.40) | 0.47 (0.12-1.40) | 0.45 (0.11-1.44) | 0.46 (0.12-1.43) | 0.45 (0.12-1.40) | 0.46 (0.12-1.33) | 0.47 (0.11-1.41) |
| **Diuretic** | 1.06 (0.82-1.47) | 1.08 (0.80-1.57) | 1.05 (0.81-1.44) | 1.05 (0.82-1.44) | 1.05 (0.82-1.44) | 1.04 (0.83-1.45) | 1.06 (0.82-1.49) | 1.06 (0.81-1.49) | 1.06 (0.82-1.48) | 1.03 (0.85-1.34) |
| **ACEi+diuretic** | 1.05 (0.75-1.52) | 1.06 (0.71-1.62) | 1.05 (0.75-1.50) | 1.04 (0.74-1.51) | 1.04 (0.74-1.51) | 0.98 (0.70-1.43) | 1.04 (0.73-1.50) | 1.05 (0.73-1.54) | 1.03 (0.72-1.50) | 1.02 (0.78-1.38) |
| **ARB+diuretic** | NA | NA | NA | NA | NA | NA | NA | NA | NA | NA |
| **ACEi+ARB** | 0.97 (0.79-1.19) | 1.00 (0.75-1.37) | 0.96 (0.79-1.19) | 0.97 (0.79-1.19) | 0.97 (0.79-1.19) | 0.95 (0.79-1.17) | 0.97 (0.79-1.21) | 0.96 (0.78-1.21) | 0.96 (0.79-1.20) | 0.97 (0.83-1.15) |
| **DRi+ACEi** | 1.32 (0.96-1.81) | 1.10 (0.86-1.43) | 1.31 (0.97-1.80) | 1.32 (0.97-1.81) | 1.32 (0.97-1.81) | 1.31 (0.97-1.77) | 1.32 (0.96-1.83) | 1.32 (0.95-1.84) | 1.32 (0.961.81) | 1.27 (0.99-1.65) |
| **DRi+ARB** | 1.00 (0.73-1.38) | 0.94 (0.67-1.34) | 1.00 (0.73-1.37) | 1.00 (0.73-1.38) | 1.00 (0.73-1.38) | 0.99 (0.73-1.35) | 1.00 (0.73-1.39) | 1.00 (0.72-1.39) | 1.00 (0.73-1.38) | 1.00 (0.77-1.30) |
| **DRi+diuretic** | 1.07 (0.63-1.91) | 1.07 (0.55-2.07) | 1.05 (0.62-1.86) | 1.06 (0.62-1.88) | 1.06 (0.62-1.88) | 1.06 (0.63-1.86) | 1.07 (0.63-1.92) | 1.07 (0.62-1.92) | 1.07 (0.62-1.92) | 1.04 (0.66-1.74) |
| **ARB+CCB** | NA | NA | NA | NA | NA | NA | NA | NA | NA | NA |
|  |  |  |  |  |  |  |  |  |  |  |
| **Residual deviance** | 82.70 | 78.62 | 79.22 | 75.42 | 75.42 | 83.77 | 81.99 | 82.44 | 83.57 | 83.47 |
| **DIC** | 579.25 | 564.04 | 568.11 | 549.07 | 549.07 | 580.38 | 579.55 | 580.17 | 581.26 | 589.97 |
| **# data points** | 76 | 73 | 74 | 70 | 70 | 76 | 76 | 76 | 76 | 76 |
| **SD** | 0.13 (0.05-0.25) | 0.16 (0.08-0.28) | 0.13 (0.05-0.25) | 0.13 (0.06-0.25) | 0.13 (0.06-0.25) | 0.12 (0.03-0.25) | 0.14 (0.06-0.26) | 0.14 (0.06-0.26) | 0.14 (0.05-0.25) | 0.10 (0.02-0.20) |

Values represent odds ratios with 95% credible intervals. Values for renin-angiotensin blockers (alone or in combination) are highlighted in dark green. RoB = risk of bias. DIC = deviance information criteria. SD = standard deviation.

ACEi = Angiotensin converting enzyme inhibitor; BB = beta-blocker; CCB = Calcium channel blocker; ARB = Angiotensin receptor blocker; ACEi+CCB = Angiotensin converting enzyme inhibitor + calcium channel blocker; ACEi+CCB = Angiotensin converting enzyme inhibitor + diuretic; ACEi+ARB = Angiotensin converting enzyme inhibitor + angiotensin receptor blocker; DRi+ACEi = Direct renin inhibitor (aliskiren) + angiotensin converting enzyme inhibitor; DRi+ARB = Direct renin inhibitor (aliskiren) + angiotensin receptor blocker; DRi+diuretic = Direct renin inhibitor (aliskiren) + diuretic.

**Table 10b. Sensitivity analyses. Cardiovascular mortality.**

|  | **Base case model** | **Exclusion of high RoB studies** | **Exclusion of small studies (<100 patients)** | **Exclusion of type 1 DM studies** | **Exclusion of normotension studies** | **Adjusted for publication year** | **Adjusted for mean age** | **Adjusted for % males** | **Adjusted for control group risk** | **Rate ratios based on patient-years** |
| --- | --- | --- | --- | --- | --- | --- | --- | --- | --- | --- |
| **ACEi** | reference | reference | reference | reference | reference | reference | reference | reference | reference | reference |
| **BB** | 1.26 (0.81-2.01) | 1.19 (0.76-1.89) | 1.14 (0.71-1.82) | 1.26 (0.81-2.01) | 1.26 (0.82-2.01) | 1.22 (0.81-1.91) | 1.27 (0.82-2.02) | 1.26 (0.81-2.06) | 1.25 (0.81-1.95) | 1.51 (0.71-3.49) |
| **CCB** | 0.96 (0.74-1.23) | 0.96 (0.74-1.24) | 0.94 (0.73-1.21) | 0.94 (0.73-1.20) | 0.97 (0.75-1.26) | 0.96 (0.76-1.21) | 0.95 (0.74-1.22) | 0.95 (0.73-1.23) | 0.96 (0.75-1.22) | 0.97 (0.62-1.54) |
| **ARB** | 1.07 (0.88-1.33) | 1.07 (0.88-1.33) | 1.06 (0.87-1.32) | 1.08 (0.89-1.34) | 1.08 (0.89-1.36) | 1.04 (0.86-1.28) | 1.08 (0.88-1.35) | 1.07 (0.88-1.35) | 1.06 (0.87-1.29) | 1.25 (0.84-1.96) |
| **ACEi+CCB** | 0.15 (0.01-0.88) | NA | 0.14 (0.00-0.88) | 0.15 (0.01-0.90) | 0.14 (0.01-0.89) | 0.15 (0.01-0.88) | 0.15 (0.01-0.92) | 0.14 (0.01-0.95) | 0.11 (0.00-0.71) | 0.16 (0.01-1.05) |
| **Diuretic** | 0.98 (0.64-1.52) | 0.98 (0.64-1.55) | 0.97 (0.63-1.54) | 0.97 (0.63-1.52) | 0.99 (0.64-1.56) | 0.98 (0.66-1.47) | 0.98 (0.63-1.55) | 0.98 (0.62-1.56) | 0.98 (0.66-1.51) | 1.06 (0.41-2.93) |
| **ACEi+diuretic** | 0.87 (0.51-1.52) | 0.87 (0.50-1.53) | 0.87 (0.50-1.53) | 0.87 (0.50-1.51) | 0.88 (0.50-1.53) | 0.78 (0.47-1.36) | 0.87 (0.50-1.54) | 0.87 (0.48-1.57) | 0.83 (0.49-1.41) | 0.84 (0.24-3.04) |
| **ARB+diuretic** | 0.77 (0.02-15.2) | 0.81 (0.02-14.9) | 0.83 (0.03-17.5) | 0.81 (0.02-15.1) | 0.73 (0.01-13.4) | 0.66 (0.01-11.9) | 0.72 (0.02-14.6) | 0.84 (0.02-17.4) | 0.88 (0.02-17.0) | 0.96 (0.02-27.5) |
| **ACEi+ARB** | 1.06 (0.79-1.49) | 1.06 (0.77-1.49) | 1.05 (0.78-1.49) | 1.06 (0.79-1.50) | 1.07 (0.79-1.52) | 1.03 (0.79-1.42) | 1.06 (0.79-1.52) | 1.06 (0.78-1.52) | 1.05 (0.79-1.46) | 1.25 (0.65-2.59) |
| **DRi+ACEi** | 1.45 (0.91-2.35) | 1.45 (0.90-2.35) | 1.45 (0.90-2.35) | 1.45 (0.91-2.37) | 1.46 (0.91-2.39) | 1.43 (0.93-2.22) | 1.46 (0.90-2.40) | 1.45 (0.89-2.40) | 1.45 (0.92-1.28) | 1.53 (0.53-4.60) |
| **DRi+ARB** | 0.98 (0.61-1.60) | 0.98 (0.61-1.60) | 0.98 (0.61-1.59) | 0.99 (0.61-1.60) | 0.99 (0.61-1.62) | 0.96 (0.62-1.51) | 0.99 (0.61-1.63) | 0.98 (0.60-1.63) | 0.98 (0.62-1.54) | 1.06 (0.37-3.14) |
| **DRi+diuretic** | 1.32 (0.63-2.84) | 1.34 (0.63-2.89) | 1.32 (0.62-2.86) | 1.32 (0.62-2.83) | 1.33 (0.62-2.91) | 1.32 (0.66-2.67) | 1.32 (0.61-2.90) | 1.32 (0.61-2.93) | 1.34 (0.66-2.79) | 1.36 (0.28-6.89) |
| **ARB+CCB** | 0.41 (0.02-5.75) | 0.44 (0.01-5.98) | 0.45 (0.02-6.48) | 0.44 (0.01-5.51) | 0.40 (0.01-5.29) | 0.37 (0.01-4.78) | 0.41 (0.01-5.78) | 0.45 (0.01-6.33) | 0.47 (0.02-6.52) | 0.52 (0.01-9.13) |
|  |  |  |  |  |  |  |  |  |  |  |
| **Residual deviance** | 107.40 | 101.2 | 99.25 | 98.53 | 96.66 | 109.50 | 107.40 | 107.40 | 109.30 | 106.60 |
| **DIC** | 577.66 | 554.71 | 552.43 | 556.74 | 545.34 | 579.01 | 578.49 | 578.73 | 580.12 | 592.29 |
| **# data points** | 96 | 90 | 89 | 90 | 88 | 96 | 96 | 96 | 96 | 96 |
| **SD** | 0.21 (0.09-0.40) | 0.21 (0.09-0.40) | 0.22 (0.10-0.40) | 0.21 (0.10-0.40) | 0.22 (0.10-0.40) | 0.19 (0.05-0.37) | 0.22 (0.10-0.41) | 0.22 (0.10-0.42) | 0.20 (0.08-0.38) | 0.57 (0.32-0.89) |

Values represent odds ratios with 95% credible intervals. Values for renin-angiotensin blockers (alone or in combination) are highlighted in dark green. RoB = risk of bias. DIC = deviance information criteria. SD = standard deviation.

ACEi = Angiotensin converting enzyme inhibitor; BB = beta-blocker; CCB = Calcium channel blocker; ARB = Angiotensin receptor blocker; ACEi+CCB = Angiotensin converting enzyme inhibitor + calcium channel blocker; ACEi+CCB = Angiotensin converting enzyme inhibitor + diuretic; ACEi+ARB = Angiotensin converting enzyme inhibitor + angiotensin receptor blocker; DRi+ACEi = Direct renin inhibitor (aliskiren) + angiotensin converting enzyme inhibitor; DRi+ARB = Direct renin inhibitor (aliskiren) + angiotensin receptor blocker; DRi+diuretic = Direct renin inhibitor (aliskiren) + diuretic.

**Table 10c. Sensitivity analyses. Myocardial infarction.**

|  | **Base case model** | **Exclusion of high RoB studies** | **Exclusion of small studies (<100 patients)** | **Exclusion of type 1 DM studies** | **Exclusion of normotension studies** | **Adjusted for publication year** | **Adjusted for mean age** | **Adjusted for % males** | **Adjusted for control group risk** | **Rate ratios based on patient-years** |
| --- | --- | --- | --- | --- | --- | --- | --- | --- | --- | --- |
| **ACEi** | reference | reference | reference | reference | reference | reference | reference | reference | reference | reference |
| **BB** | 1.01 (0.68-1.52) | 1.05 (0.70-1.63) | 1.02 (0.67-1.54) | 1.06 (0.71-1.63) | 1.03 (0.68-1.54) | 1.02 (0.68-1.52) | 1.03 (0.71-1.49) | 1.01 (0.66-1.54) | 1.02 (0.68-1.54) | 1.02 (0.70-1.48) |
| **CCB** | 1.09 (0.89-1.39) | 1.09 (0.89-1.40) | 1.09 (0.90-1.40) | 1.10 (0.91-1.43) | 1.10 (0.89-1.44) | 1.09 (0.89-1.40) | 1.08 (0.90-1.34) | 1.09 (0.88-1.41) | 1.09 (0.90-1.40) | 1.07 (0.89-1.35) |
| **ARB** | 1.07 (0.89-1.28) | 1.07 (0.89-1.29) | 1.07 (0.90-1.28) | 1.08 (0.91-1.31) | 1.08 (0.90-1.31) | 1.05 (0.88-1.28) | 1.09 (0.93-1.28) | 1.06 (0.87-1.28) | 1.06 (0.88-1.28) | 1.05 (0.90-1.24) |
| **ACEi+CCB** | 0.45 (0.06-2.18) | 12.51 (0.11-1977.0) | 0.45 (0.06-2.21) | 0.47 (0.05-2.26) | 0.45 (0.06-2.22) | 0.49 (0.06-2.27) | 0.44 (0.05-1.94) | 0.47 (0.05-2.17) | 0.47 (0.06-2.43) | 0.43 (0.05-1.95) |
| **Diuretic** | 1.12 (0.81-1.82) | 1.13 (0.81-1.86) | 1.08 (0.78-1.69) | 1.13 (0.82-1.84) | 1.12 (0.81-1.86) | 1.12 (0.81-1.84) | 1.09 (0.83-1.65) | 1.13 (0.81-1.88) | 1.13 (0.81-1.86) | 1.10 (0.83-1.68) |
| **ACEi+diuretic** | NA | NA | NA | NA | NA | NA | NA | NA | NA | NA |
| **ARB+diuretic** | 3.06 (0.37-39.8) | 11.48 (0.83-393.8) | 3.09 (0.39-34.8) | 3.39 (0.37-35.0) | 3.36 (0.36-38.8) | 3.22 (0.40-28.2) | 3.82 (0.46-116.7) | 3.28 (0.37-34.6) | 2.85 (0.33-38.7) | 2.96 (0.37-44.35) |
| **ACEi+ARB** | 1.00 (0.78-1.33) | 1.00 (0.77-1.34) | 1.00 (0.78-1.32) | 1.01 (0.78-1.35) | 1.01 (0.78-1.35) | 0.99 (0.76-1.33) | 1.01 (0.81-1.29) | 1.00 (0.76-1.35) | 0.99 (0.76-1.34) | 1.00 (0.79-1.28) |
| **DRi+ACEi** | 1.35 (0.85-2.12) | 1.36 (0.85-2.14) | 1.36 (0.87-2.11) | 1.37 (0.87-2.14) | 1.36 (0.86-2.15) | 1.35 (0.85-2.13) | 1.37 (0.91-2.03) | 1.34 (0.84-2.16) | 1.35 (0.85-2.12) | 1.32 (0.88-1.99) |
| **DRi+ARB** | 0.83 (0.52-1.31) | 0.83 (0.51-1.33) | 0.83 (0.52-1.31) | 0.84 (0.52-1.33) | 0.83 (0.52-1.33) | 0.82 (0.51-1.32) | 0.83 (0.54-1.27) | 0.82 (0.50-1.33) | 0.83 (0.51-1.33) | 0.83 (0.54-1.26) |
| **DRi+diuretic** | 0.61 (0.24-1.60) | 0.62 (0.24-1.62) | 0.59 (0.23-1.50) | 0.62 (0.24-1.61) | 0.62 (0.25-1.63) | 0.62 (0.24-1.61) | 0.60 (0.25-1.49) | 0.63 (0.24-1.67) | 0.62 (0.25-1.62) | 0.61 (0.26-1.53) |
| **ARB+CCB** | 1.45 (0.21-14.4) | 3.73 (0.42-94.8) | 1.45 (0.22-12.9) | 1.51 (0.22-13.2) | 1.57 (0.22-14.7) | 1.52 (0.23-11.4) | 1.76 (0.21-40.6) | 1.51 (0.23-12.7) | 1.36 (0.21-12.6) | 1.52 (0.22-15.4) |
|  |  |  |  |  |  |  |  |  |  |  |
| **Residual deviance** | 125.60 | 115.70 | 104.00 | 106.30 | 109.40 | 125.50 | 123.60 | 124.80 | 126.30 | 125.20 |
| **DIC** | 618.75 | 586.36 | 567.14 | 566.00 | 567.34 | 618.92 | 614.93 | 619.28 | 620.36 | 621.87 |
| **# data points** | 104 | 100 | 90 | 94 | 96 | 104 | 104 | 104 | 104 | 104 |
| **SD** | 0.18 (0.01-0.36) | 0.18 (0.02-0.37) | 0.17 (0.01-0.35) | 0.17 (0.01-0.36) | 0.18 (0.02-0.37) | 0.18 (0.01-0.37) | 0.13 (0.02-0.31) | 0.19 (0.03-0.38) | 0.18 (0.02-0.37) | 0.14 (0.02-0.32) |

Values represent odds ratios with 95% credible intervals. Values for renin-angiotensin blockers (alone or in combination) are highlighted in dark green. RoB = risk of bias. DIC = deviance information criteria. SD = standard deviation.

ACEi = Angiotensin converting enzyme inhibitor; BB = beta-blocker; CCB = Calcium channel blocker; ARB = Angiotensin receptor blocker; ACEi+CCB = Angiotensin converting enzyme inhibitor + calcium channel blocker; ACEi+CCB = Angiotensin converting enzyme inhibitor + diuretic; ACEi+ARB = Angiotensin converting enzyme inhibitor + angiotensin receptor blocker; DRi+ACEi = Direct renin inhibitor (aliskiren) + angiotensin converting enzyme inhibitor; DRi+ARB = Direct renin inhibitor (aliskiren) + angiotensin receptor blocker; DRi+diuretic = Direct renin inhibitor (aliskiren) + diuretic.

**Table 10d. Sensitivity analyses. Stroke.**

|  | **Base case model** | **Exclusion of high RoB studies** | **Exclusion of small studies (<100 patients)** | **Exclusion of type 1 DM studies** | **Exclusion of normotension studies** | **Adjusted for publication year** | **Adjusted for mean age** | **Adjusted for % males** | **Adjusted for control group risk** | **Rate ratios based on patient-years** |
| --- | --- | --- | --- | --- | --- | --- | --- | --- | --- | --- |
| **ACEi** | reference | reference | reference | reference | reference | reference | reference | reference | reference | reference |
| **BB** | 1.15 (0.80-1.66) | 1.15 (0.80-1.69) | 1.15 (0.78-1.66) | 1.19 (0.80-1.76) | 1.15 (0.79-1.70) | 1.14 (0.78-1.66) | 1.16 (0.78-1.71) | 1.15 (0.79-1.69) | 1.14 (0.78-1.68) | 1.13 (0.80-1.61) |
| **CCB** | 0.88 (0.75-1.05) | 0.89 (0.76-1.08) | 0.87 (0.74-1.04) | 0.87 (0.74-1.04) | 0.85 (0.72-1.01) | 0.88 (0.74-1.08) | 0.89 (0.75-1.07) | 0.88 (0.75-1.06) | 0.89 (0.75-1.07) | 0.89 (0.75-1.05) |
| **ARB** | 1.01 (0.88-1.16) | 1.02 (0.89-1.17) | 1.00 (0.88-1.15) | 1.00 (0.88-1.15) | 1.00 (0.87-1.15) | 0.98 (0.85-1.14) | 1.01 (0.88-1.17) | 1.01 (0.88-1.17) | 1.01 (0.88-1.17) | 1.01 (0.89-1.16) |
| **ACEi+CCB** | 0.31 (0.01-2.30) | NA | 0.26 (0.00-1.92) | 0.21 (0.02-3.90) | 0.32 (0.01-2.35) | 0.27 (0.03-1.62) | 0.25 (0.00-1.76) | 0.31 (0.01-2.02) | 0.27 (0.01-2.53) | 0.43 (0.05-2.32) |
| **Diuretic** | 0.97 (0.78-1.26) | 0.98 (0.79-1.28) | 0.97 (0.78-1.25) | 0.97 (0.78-1.21) | 0.96 (0.77-1.23) | 0.97 (0.78-1.26) | 0.98 (0.78-1.29) | 0.98 (0.79-1.29) | 0.98 (0.78-1.31) | 0.98 (0.79-1.25) |
| **ACEi+diuretic** | 1.05 (0.81-1.41) | 1.05 (0.80-1.42) | 1.04 (0.78-1.40) | 1.06 (0.81-1.42) | 1.06 (0.80-1.41) | 0.98 (0.73-1.36) | 1.06 (0.80-1.47) | 1.05 (0.79-1.44) | 1.05 (0.78-1.46) | 1.06 (0.81-1.43) |
| **ARB+diuretic** | 0.75 (0.19-2.90) | 0.80 (0.26-2.74) | 0.80 (0.24-2.88) | 0.78 (0.24-3.24) | 0.84 (0.24-2.55) | 0.82 (0.22-2.81) | 0.81 (0.26-2.71) | 0.84 (0.25-3.06) | 0.80 (0.24-2.70) | 0.83 (0.24-3.15) |
| **ACEi+ARB** | 0.88 (0.71-1.08) | 0.88 (0.71-1.08) | 0.88 (0.71-1.07) | 0.87 (0.71-1.07) | 0.88 (0.70-1.06) | 0.87 (0.69-1.07) | 0.88 (0.70-1.08) | 0.88 (0.70-1.08) | 0.87 (0.70-1.08) | 0.89 (0.72-1.07) |
| **DRi+ACEi** | 1.19 (0.82-1.67) | 1.19 (0.82-1.68) | 1.18 (0.83-1.66) | 1.19 (0.85-1.73) | 1.17 (0.83-1.63) | 1.15 (0.83-1.63) | 1.17 (0.82-1.72) | 1.19 (0.82-1.68) | 1.18 (0.81-1.69) | 1.15 (0.80-1.62) |
| **DRi+ARB** | 1.28 (0.91-1.78) | 1.28 (0.93-1.80) | 1.28 (0.92-1.77) | 1.30 (0.93-1.75) | 1.27 (0.92-1.76) | 1.27 (0.91-1.74) | 1.27 (0.91-1.80) | 1.28 (0.91-1.80) | 1.29 (0.91-1.80) | 1.26 (0.92-1.75) |
| **DRi+diuretic** | 0.45 (0.16-1.20) | 0.51 (0.18-1.36) | 0.45 (0.14-1.26) | 0.44 (0.16-1.19) | 0.47 (0.15-1.27) | 0.46 (0.14-1.18) | 0.46 (0.15-1.21) | 0.50 (0.16-1.32) | 0.46 (0.15-1.27) | 0.45 (0.14-1.19) |
| **ARB+CCB** | 1.21 (0.40-3.67) | 1.22 (0.48-3.53) | 1.21 (0.45-3.44) | 1.23 (0.46-3.58) | 1.27 (0.44-3.34) | 1.23 (0.43-4.19) | 1.23 (0.48-3.28) | 1.26 (0.46-3.73) | 1.20 (0.48-3.29) | 1.25 (0.47-3.62) |
|  |  |  |  |  |  |  |  |  |  |  |
| **Residual deviance** | 96.40 | 88.31 | 84.91 | 88.82 | 84.64 | 95.61 | 96.69 | 96.86 | 96.37 | 94.39 |
| **DIC** | 581.58 | 553.97 | 550.54 | 555.28 | 542.16 | 581.11 | 583.14 | 583.40 | 583.21 | 583.33 |
| **# data points** | 95 | 88 | 86 | 89 | 87 | 95 | 95 | 95 | 95 | 95 |
| **SD** | 0.06 (0.00-0.20) | 0.07 (0.00-0.21) | 0.07 (0.00-0.20) | 0.06 (0.00-0.19) | 0.06 (0.00-0.19) | 0.06 (0.00-0.20) | 0.08 (0.01-0.21) | 0.07 (0.00-0.22) | 0.08 (0.00-0.23) | 0.07 (0.01-0.19) |

Values represent odds ratios with 95% credible intervals. Values for renin-angiotensin blockers (alone or in combination) are highlighted in dark green. RoB = risk of bias. DIC = deviance information criteria. SD = standard deviation.

ACEi = Angiotensin converting enzyme inhibitor; BB = beta-blocker; CCB = Calcium channel blocker; ARB = Angiotensin receptor blocker; ACEi+CCB = Angiotensin converting enzyme inhibitor + calcium channel blocker; ACEi+CCB = Angiotensin converting enzyme inhibitor + diuretic; ACEi+ARB = Angiotensin converting enzyme inhibitor + angiotensin receptor blocker; DRi+ACEi = Direct renin inhibitor (aliskiren) + angiotensin converting enzyme inhibitor; DRi+ARB = Direct renin inhibitor (aliskiren) + angiotensin receptor blocker; DRi+diuretic = Direct renin inhibitor (aliskiren) + diuretic.

**Table 10e. Sensitivity analyses. Progression of renal disease.**

|  | **Base case model** | **Exclusion of high RoB studies** | **Exclusion of small studies (<100 patients)** | **Exclusion of type 1 DM studies** | **Exclusion of normotension studies** | **Adjusted for publication year** | **Adjusted for mean age** | **Adjusted for % males** | **Adjusted for control group risk** | **Rate ratios based on patient-years** |
| --- | --- | --- | --- | --- | --- | --- | --- | --- | --- | --- |
| **ACEi** | reference | reference | reference | reference | reference | reference | reference | reference | reference | reference |
| **BB** | 9.80 (3.37-45.05) | NA | NA | 9.57 (2.39-44.14) | 9.57 (2.39-44.14) | 9.84 (2.35-47.18) | 9.50 (2.26-45.27) | 9.23 (2.39-44.41) | 9.49 (2.46-45.65) | 4.09 (1.27-15.61) |
| **CCB** | 1.17 (0.90-1.64) | 1.17 (0.89-1.64) | 1.17 (0.89-1.64) | 1.17 (0.89-1.63) | 1.17 (0.89-1.63) | 1.19 (0.91-1.68) | 1.17 (0.89-1.65) | 1.13 (0.88-1.54) | 1.19 (0.94-1.58) | 0.97 (0.69-1.41) |
| **ARB** | 1.10 (0.90-1.40) | 1.09 (0.90-1.63) | 1.09 (0.90-1.63) | 1.08 (0.89-1.37) | 1.08 (0.89-1.37) | 1.09 (0.89-1.40) | 1.09 (0.88-1.39) | 1.01 (0.82-1.28) | 1.07 (0.90-1.30) | 1.25 (0.97-1.63) |
| **ACEi+CCB** | NA | NA | NA | NA | NA | NA | NA | NA | NA | NA |
| **Diuretic** | 1.14 (0.78-1.74) | 1.14 (0.77-1.75) | 1.14 (0.77-1.75) | 1.14 (0.78-1.74) | 1.14 (0.78-1.74) | 1.15 (0.78-1.78) | 1.14 (0.76-1.77) | 1.12 (0.79-1.66) | 1.15 (0.85-1.62) | 1.03 (0.63-1.72) |
| **ACEi+diuretic** | 0.98 (0.61-1.62) | 0.98 (0.60-1.63) | 0.98 (0.60-1.63) | 0.99 (0.62-1.65) | 0.99 (0.62-1.65) | 0.94 (0.58-1.59) | 1.03 (0.62-1.82) | 0.82 (0.50-1.35) | 0.87 (0.59-1.32) | 0.93 (0.51-1.73) |
| **ARB+diuretic** | NA | NA | NA | NA | NA | NA | NA | NA | NA | NA |
| **ACEi+ARB** | 0.97 (0.72-1.29) | 0.97 (0.72-1.29) | 0.97 (0.72-1.29) | 0.97 (0.72-1.27) | 0.97 (0.72-1.27) | 0.96 (0.71-1.28) | 0.96 (0.70-1.29) | 0.93 (0.70-1.21) | 0.99 (0.77-1.24) | 1.05 (0.75-1.48) |
| **DRi+ACEi** | 0.99 (0.65-1.57) | 0.99 (0.64-1.58) | 0.99 (0.64-1.58) | 0.98 (0.64-1.55) | 0.98 (0.64-1.55) | 0.99 (0.64-1.57) | 0.99 (0.63-1.59) | 0.94 (0.63-1.45) | 0.98 (0.67-1.44) | 1.07 (0.63-1.85) |
| **DRi+ARB** | 1.18 (0.78-1.84) | 1.18 (0.77-1.85) | 1.18 (0.77-1.85) | 1.17 (0.77-1.81) | 1.17 (0.77-1.81) | 1.18 (0.77-1.86) | 1.18 (0.76-1.86) | 1.12 (0.76-1.70) | 1.17 (0.82-1.69) | 1.26 (0.74-2.13) |
| **DRi+diuretic** | NA | NA | NA | NA | NA | NA | NA | NA | NA | NA |
| **ARB+CCB** | NA | NA | NA | NA | NA | NA | NA | NA | NA | NA |
|  |  |  |  |  |  |  |  |  |  |  |
| **Residual deviance** | 44.51 | 42.17 | 42.17 | 41.00 | 41.00 | 44.55 | 43.75 | 43.15 | 40.66 | 42.43 |
| **DIC** | 344.58 | 331.68 | 331.68 | 322.75 | 322.75 | 345.07 | 344.94 | 343.17 | 340.30 | 354.15 |
| **# data points** | 44 | 41 | 41 | 40 | 40 | 44 | 44 | 44 | 44 | 44 |
| **SD** | 0.18 (0.07-0.36) | 0.19 (0.07-0.37) | 0.19 (0.07-0.37) | 0.18 (0.06-0.37) | 0.18 (0.06-0.37) | 0.18 (0.07-0.38) | 0.20 (0.09-0.38) | 0.17 (0.06-0.34) | 0.15 (0.05-0.29) | 0.25 (0.15-0.43) |

Values represent odds ratios with 95% credible intervals. Values for renin-angiotensin blockers (alone or in combination) are highlighted in dark green. RoB = risk of bias. DIC = deviance information criteria. SD = standard deviation.

ACEi = Angiotensin converting enzyme inhibitor; BB = beta-blocker; CCB = Calcium channel blocker; ARB = Angiotensin receptor blocker; ACEi+CCB = Angiotensin converting enzyme inhibitor + calcium channel blocker; ACEi+CCB = Angiotensin converting enzyme inhibitor + diuretic; ACEi+ARB = Angiotensin converting enzyme inhibitor + angiotensin receptor blocker; DRi+ACEi = Direct renin inhibitor (aliskiren) + angiotensin converting enzyme inhibitor; DRi+ARB = Direct renin inhibitor (aliskiren) + angiotensin receptor blocker; DRi+diuretic = Direct renin inhibitor (aliskiren) + diuretic.

**Table 10f. Sensitivity analyses. End-stage renal disease.**

|  | **Base case model** | **Exclusion of high RoB studies** | **Exclusion of small studies (<100 patients)** | **Exclusion of type 1 DM studies** | **Exclusion of normotension studies** | **Adjusted for publication year** | **Adjusted for mean age** | **Adjusted for % males** | **Adjusted for control group risk** | **Rate ratios based on patient-years** |
| --- | --- | --- | --- | --- | --- | --- | --- | --- | --- | --- |
| **ACEi** | reference | reference | reference | reference | reference | reference | reference | reference | reference | reference |
| **BB** | 4.88 (1.17-26.34) | 1.01 (0.03-43.30) | NA | 15.96 (1.86-554.90) | 4.67 (1.12-28.26) | 5.01 (1.16-26.30) | 4.42 (1.14-26.40) | 3.94 (0.99-20.49) | 5.14 (1.11-32.63) | 3.66 (1.09-13.29) |
| **CCB** | 1.27 (0.91-1.75) | 1.29 (0.93-1.77) | 1.29 (0.92-1.79) | 1.25 (0.86-1.76) | 1.26 (0.88-1.73) | 1.27 (0.89-1.77) | 1.26 (0.88-1.73) | 1.25 (0.88-1.73) | 1.30 (0.94-1.82) | 1.25 (0.93-1.64) |
| **ARB** | 1.09 (0.84-1.42) | 1.08 (0.84-1.41) | 1.09 (0.84-1.42) | 1.06 (0.79-1.41) | 1.08 (0.82-1.40) | 1.08 (0.83-1.44) | 1.06 (0.80-1.40) | 1.09 (0.82-1.42) | 1.12 (0.86-1.47) | 1.09 (0.87-1.37) |
| **ACEi+CCB** | NA | NA | NA | NA | NA | NA | NA | NA | NA | NA |
| **Diuretic** | 0.95 (0.59-1.50) | 0.95 (0.60-1.50) | 0.95 (0.60-1.51) | 0.94 (0.56-1.54) | 0.95 (0.58-1.50) | 0.94 (0.57-1.55) | 0.95 (0.58-1.53) | 0.93 (0.56-1.52) | 0.96 (0.60-1.51) | 0.94 (0.63-1.41) |
| **ACEi+diuretic** | 1.75 (0.81-3.80) | 1.72 (0.80-3.69) | 1.73 (0.81-3.77) | 1.65 (0.73-3.69) | 1.71 (0.78-3.70) | 1.77 (0.76-4.07) | 1.54 (0.65-3.52) | 1.87 (0.79-4.51) | 1.21 (0.53-3.06) | 1.66 (0.81-3.43) |
| **ARB+diuretic** | NA | NA | NA | NA | NA | NA | NA | NA | NA | NA |
| **ACEi+ARB** | 0.91 (0.63-1.27) | 0.91 (0.63-1.26) | 0.91 (0.64-1.27) | 0.89 (0.60-1.26) | 0.90 (0.62-1.26) | 0.92 (0.62-1.31) | 0.90 (0.62-1.26) | 0.91 (0.62-1.28) | 0.95 (0.65-1.33) | 0.94 (0.68-1.26) |
| **DRi+ACEi** | 1.09 (0.66-1.85) | 1.10 (0.66-1.81) | 1.10 (0.66-1.83) | 1.07 (0.63-1.84) | 1.09 (0.65-1.84) | 1.09 (0.64-1.87) | 1.09 (0.64-1.81) | 1.11 (0.63-1.85) | 1.12 (0.67-1.85) | 1.11 (0.70-1.72) |
| **DRi+ARB** | 1.17 (0.72-1.93) | 1.17 (0.72-1.90) | 1.17 (0.72-1.93) | 1.15 (0.68-1.96) | 1.16 (0.71-1.92) | 1.16 (0.70-1.98) | 1.16 (0.70-1.90) | 1.16 (0.69-1.95) | 1.18 (0.74-1.95) | 1.17 (0.75-1.80) |
| **DRi+diuretic** | NA | NA | NA | NA | NA | NA | NA | NA | NA | NA |
| **ARB+CCB** | NA | NA | NA | NA | NA | NA | NA | NA | NA | NA |
|  |  |  |  |  |  |  |  |  |  |  |
| **Residual deviance** | 56.79 | 44.32 | 38.14 | 46.61 | 51.68 | 57.21 | 56.31 | 56.94 | 56.75 | 54.93 |
| **DIC** | 305.76 | 279.27 | 258.16 | 268.59 | 290.57 | 307.43 | 306.07 | 306.91 | 306.56 | 306.75 |
| **# data points** | 52 | 45 | 39 | 44 | 48 | 52 | 52 | 52 | 52 | 52 |
| **SD** | 0.16 (0.01-0.43) | 0.16 (0.01-0.41) | 0.16 (0.01-0.42) | 0.18 (0.02-0.47) | 0.16 (0.01-0.44) | 0.18 (0.02-0.46) | 0.16 (0.01-0.46) | 0.17 (0.00-0.48) | 0.16 (0.01-0.42) | 0.12 (0.01-0.36) |

Values represent odds ratios with 95% credible intervals. Values for renin-angiotensin blockers (alone or in combination) are highlighted in dark green. RoB = risk of bias. DIC = deviance information criteria. SD = standard deviation.

ACEi = Angiotensin converting enzyme inhibitor; BB = beta-blocker; CCB = Calcium channel blocker; ARB = Angiotensin receptor blocker; ACEi+CCB = Angiotensin converting enzyme inhibitor + calcium channel blocker; ACEi+CCB = Angiotensin converting enzyme inhibitor + diuretic; ACEi+ARB = Angiotensin converting enzyme inhibitor + angiotensin receptor blocker; DRi+ACEi = Direct renin inhibitor (aliskiren) + angiotensin converting enzyme inhibitor; DRi+ARB = Direct renin inhibitor (aliskiren) + angiotensin receptor blocker; DRi+diuretic = Direct renin inhibitor (aliskiren) + diuretic.

**Table 10g. Sensitivity analyses. Doubling of serum creatinine.**

|  | **Base case model** | **Exclusion of high RoB studies** | **Exclusion of small studies (<100 patients)** | **Exclusion of type 1 DM studies** | **Exclusion of normotension studies** | **Adjusted for publication year** | **Adjusted for mean age** | **Adjusted for % males** | **Adjusted for control group risk** | **Rate ratios based on patient-years** |
| --- | --- | --- | --- | --- | --- | --- | --- | --- | --- | --- |
| **ACEi** | reference | reference | reference | reference | reference | reference | reference | reference | reference | reference |
| **BB** | 6.49 (1.22-38.88) | NA | NA | 6.29 (1.10-38.64) | 6.59 (1.19-40.44) | 6.52 (1.20-38.11) | 6.75 (1.16-47.15) | 6.46 (1.13-40.83) | 6.81 (1.33-45.73) | 4.69 (0.98-21.54) |
| **CCB** | 1.40 (0.95-2.19) | 1.39 (0.92-2.19) | 1.39 (0.92-2.24) | 1.35 (0.93-2.07) | 1.33 (0.93-1.93) | 1.38 (0.95-2.08) | 1.41 (0.94-2.28) | 1.40 (0.93-2.24) | 1.45 (1.04-2.10) | 1.30 (0.97-1.84) |
| **ARB** | 1.26 (0.97-1.79) | 1.26 (0.97-1.81) | 1.27 (0.96-1.83) | 1.17 (0.90-1.63) | 1.14 (0.89-1.51) | 1.18 (0.91-1.61) | 1.27 (0.97-1.83) | 1.28 (0.97-1.86) | 1.22 (0.96-1.60) | 1.19 (0.97-1.56) |
| **ACEi+CCB** | NA | NA | NA | NA | NA | NA | NA | NA | NA | NA |
| **Diuretic** | 1.17 (0.65-2.15) | 1.16 (0.64-2.18) | 1.17 (0.63-2.24) | 1.15 (0.66-2.05) | 1.14 (0.69-1.90) | 1.16 (0.67-2.05) | 1.17 (0.64-2.26) | 1.17 (0.63-2.25) | 1.19 (0.74-1.95) | 1.13 (0.73-1.78) |
| **ACEi+diuretic** | 1.75 (0.84-3.84) | 1.75 (0.82-3.92) | 1.76 (0.81-3.98) | 1.74 (0.85-3.70) | 1.66 (0.85-3.28) | 1.45 (0.71-3.10) | 1.77 (0.80-4.29) | 1.81 (0.85-4.14) | 1.10 (0.55-2.20) | 1.60 (0.89-3.01) |
| **ARB+diuretic** | NA | NA | NA | NA | NA | NA | NA | NA | NA | NA |
| **ACEi+ARB** | 0.99 (0.65-1.56) | 1.00 (0.64-1.56) | 0.99 (0.63-1.59) | 0.97 (0.64-1.47) | 0.96 (0.65-1.39) | 0.93 (0.61-1.40) | 1.00 (0.64-1.60) | 0.99 (0.63-1.59) | 1.04 (0.72-1.52) | 1.02 (0.74-1.43) |
| **DRi+ACEi** | 0.93 (0.53-1.74) | 0.94 (0.53-1.77) | 0.94 (0.52-1.81) | 0.90 (0.52-1.63) | 0.88 (0.54-1.50) | 0.90 (0.52-1.61) | 0.94 (0.51-1.81) | 0.94 (0.52-1.82) | 0.92 (0.57-1.52) | 0.90 (0.59-1.47) |
| **DRi+ARB** | 1.27 (0.73-2.35) | 1.27 (0.73-2.38) | 1.27 (0.71-2.43) | 1.22 (0.72-2.17) | 1.20 (0.75-1.99) | 1.22 (0.73-2.18) | 1.28 (0.71-2.45) | 1.28 (0.72-2.45) | 1.25 (0.79-2.04) | 1.22 (0.81-1.94) |
| **DRi+diuretic** | NA | NA | NA | NA | NA | NA | NA | NA | NA | NA |
| **ARB+CCB** | 1.81 (0.72-4.62) | 1.79 (0.70-4.69) | 1.80 (0.69-4.84) | 1.79 (0.74-4.34) | 1.76 (0.79-3.92) | 1.80 (0.75-4.36) | 1.81 (0.69-4.90) | 1.80 (0.69-4.91) | 1.84 (0.84-4.03) | 1.62 (0.80-3.31) |
|  |  |  |  |  |  |  |  |  |  |  |
| **Residual deviance** | 57.83 | 54.26 | 51.56 | 48.54 | 44.04 | 55.73 | 57.95 | 57.69 | 54.52 | 58.48 |
| **DIC** | 371.68 | 352.26 | 336.31 | 319.51 | 316.38 | 369.58 | 372.96 | 372.65 | 367.46 | 376.91 |
| **# data points** | 56 | 51 | 47 | 46 | 46 | 56 | 56 | 56 | 56 | 56 |
| **SD** | 0.26 (0.08-0.51) | 0.26 (0.09-0.26) | 0.27 (0.09-0.55) | 0.23 (0.05-0.50) | 0.21 (0.05-0.43) | 0.24 (0.06-0.48) | 0.28 (0.10-0.55) | 0.28 (0.08-0.54) | 0.20 (0.04-0.40) | 0.17 (0.02-0.38) |

Values represent odds ratios with 95% credible intervals. Values for renin-angiotensin blockers (alone or in combination) are highlighted in dark green. RoB = risk of bias. DIC = deviance information criteria. SD = standard deviation.

ACEi = Angiotensin converting enzyme inhibitor; BB = beta-blocker; CCB = Calcium channel blocker; ARB = Angiotensin receptor blocker; ACEi+CCB = Angiotensin converting enzyme inhibitor + calcium channel blocker; ACEi+CCB = Angiotensin converting enzyme inhibitor + diuretic; ACEi+ARB = Angiotensin converting enzyme inhibitor + angiotensin receptor blocker; DRi+ACEi = Direct renin inhibitor (aliskiren) + angiotensin converting enzyme inhibitor; DRi+ARB = Direct renin inhibitor (aliskiren) + angiotensin receptor blocker; DRi+diuretic = Direct renin inhibitor (aliskiren) + diuretic.

**Table 10h. Sensitivity analyses. All-cause mortality.**

|  | **Base case model** | **Exclusion of high RoB studies** | **Exclusion of small studies (<100 patients)** | **Exclusion of type 1 DM studies** | **Exclusion of normotension studies** | **Adjusted for publication year** | **Adjusted for mean age** | **Adjusted for % males** | **Adjusted for control group risk** | **Rate ratios based on patient-years** |
| --- | --- | --- | --- | --- | --- | --- | --- | --- | --- | --- |
| **ACEi** | reference | reference | reference | reference | reference | reference | reference | reference | reference | reference |
| **BB** | 1.38 (1.02-1.88) | 1.32 (0.97-1.80) | 1.26 (0.92-1.73) | 1.36 (1.02-1.86) | 1.38 (1.02-1.91) | 1.33 (1.01-1.77) | 1.38 (1.02-1.89) | 1.37 (1.01-1.89) | 1.36 (1.02-1.86) | 1.30 (1.00-1.70) |
| **CCB** | 0.98 (0.84-1.14) | 0.98 (0.84-1.15) | 0.97 (0.83-1.13) | 0.97 (0.83-1.13) | 0.96 (0.81-1.14) | 0.97 (0.86-1.10) | 0.98 (0.84-1.14) | 0.97 (0.83-1.14) | 0.98 (0.85-1.15) | 0.98 (0.86-1.12) |
| **ARB** | 1.04 (0.92-1.18) | 1.04 (0.92-1.18) | 1.03 (0.91-1.16) | 1.03 (0.92-1.17) | 1.04 (0.91-1.19) | 1.00 (0.91-1.11) | 1.04 (0.92-1.18) | 1.04 (0.92-1.18) | 1.03 (0.91-1.16) | 1.03 (0.94-1.15) |
| **ACEi+CCB** | 0.47 (0.13-1.14) | 0.40 (0.08-1.35) | 0.46 (0.14-1.17) | 0.45 (0.14-1.15) | 0.47 (0.14-1.15) | 0.45 (0.13-1.05) | 0.46 (0.15-1.13) | 0.43 (0.16-1.14) | 0.39 (0.13-1.01) | 0.48 (0.14-1.01) |
| **Diuretic** | 1.04 (0.81-1.34) | 1.04 (0.81-1.35) | 1.04 (0.81-1.33) | 1.04 (0.81-1.35) | 1.03 (0.79-1.37) | 1.04 (0.87-1.25) | 1.04 (0.81-1.35) | 1.04 (0.80-1.37) | 1.05 (0.82-1.35) | 1.03 (0.85-1.26) |
| **ACEi+diuretic** | 0.90 (0.67-1.26) | 0.91 (0.67-1.27) | 0.89 (0.65-1.23) | 0.91 (0.67-1.26) | 0.92 (0.66-1.34) | 0.79 (0.62-1.03) | 0.91 (0.67-1.28) | 0.90 (0.65-1.28) | 0.86 (0.64-1.21) | 0.90 (0.71-1.18) |
| **ARB+diuretic** | 1.19 (0.35-3.97) | 1.24 (0.34-4.51) | 1.17 (0.32-4.27) | 1.21 (0.33-4.22) | 1.17 (0.31-4.23) | 1.17 (0.29-3.54) | 1.15 (0.31-4.35) | 1.16 (0.34-4.01) | 1.16 (0.32-4.20) | 1.04 (0.30-3.81) |
| **ACEi+ARB** | 1.03 (0.88-1.21) | 1.03 (0.88-1.22) | 1.03 (0.88-1.21) | 1.03 (0.88-1.21) | 1.03 (0.85-1.25) | 1.02 (0.90-1.15) | 1.04 (0.88-1.22) | 1.03 (0.87-1.22) | 1.03 (0.88-1.21) | 1.03 (0.90-1.18) |
| **DRi+ACEi** | 1.29 (0.96-1.72) | 1.29 (0.96-1.73) | 1.28 (0.96-1.71) | 1.28 (0.95-1.72) | 1.29 (0.94-1.76) | 1.27 (1.00-1.60) | 1.28 (0.95-1.73) | 1.29 (0.95-1.75) | 1.28 (0.95-1.72) | 1.26 (0.98-1.62) |
| **DRi+ARB** | 0.90 (0.67-1.21) | 0.90 (0.67-1.22) | 0.90 (0.67-1.20) | 0.90 (0.67-1.21) | 0.90 (0.66-1.24) | 0.89 (0.70-1.12) | 0.90 (0.67-1.22) | 0.90 (0.66-1.23) | 0.90 (0.67-1.20) | 0.91 (0.70-1.18) |
| **DRi+diuretic** | 1.53 (0.91-2.76) | 1.53 (0.91-2.60) | 1.52 (0.89-2.58) | 1.52 (0.90-2.58) | 1.52 (0.87-2.64) | 1.50 (0.96-2.40) | 1.53 (0.91-2.59) | 1.53 (0.90-2.62) | 1.53 (0.91-2.59) | 1.42 (0.90-2.20) |
| **ARB+CCB** | 0.88 (0.31-2.47) | 0.92 (0.29-2.76) | 0.88 (0.28-2.63) | 0.89 (0.28-2.61) | 0.86 (0.28-2.65) | 0.84 (0.25-2.36) | 0.85 (0.27-2.52) | 0.87 (0.29-2.55) | 0.86 (0.27-2.53) | 0.81 (0.21-2.48) |
|  |  |  |  |  |  |  |  |  |  |  |
| **Residual deviance** | 148.10 | 137.00 | 117.90 | 123.00 | 117.5 | 149.60 | 148.80 | 147.90 | 147.90 | 148.60 |
| **DIC** | 784.78 | 745.64 | 716.86 | 702.80 | 673.22 | 782.51 | 786.77 | 785.96 | 785.67 | 794.25 |
| **# data points** | 135 | 123 | 114 | 113 | 107 | 135 | 135 | 145 | 135 | 135 |
| **SD** | 0.11 (0.03-0.22) | 0.12 (0.03-0.22) | 0.11 (0.02-0.22) | 0.12 (0.03-0.22) | 0.13 (0.04-0.25) | 0.06 (0.00-0.16) | 0.12 (0.02-0.23) | 0.12 (0.03-0.23) | 0.11 (0.02-0.22) | 0.08 (0.01-0.18) |

Values represent odds ratios with 95% credible intervals. Values for renin-angiotensin blockers (alone or in combination) are highlighted in dark green. RoB = risk of bias. DIC = deviance information criteria. SD = standard deviation.

ACEi = Angiotensin converting enzyme inhibitor; BB = beta-blocker; CCB = Calcium channel blocker; ARB = Angiotensin receptor blocker; ACEi+CCB = Angiotensin converting enzyme inhibitor + calcium channel blocker; ACEi+CCB = Angiotensin converting enzyme inhibitor + diuretic; ACEi+ARB = Angiotensin converting enzyme inhibitor + angiotensin receptor blocker; DRi+ACEi = Direct renin inhibitor (aliskiren) + angiotensin converting enzyme inhibitor; DRi+ARB = Direct renin inhibitor (aliskiren) + angiotensin receptor blocker; DRi+diuretic = Direct renin inhibitor (aliskiren) + diuretic.

**Table 10i. Sensitivity analyses. Angina pectoris.**

|  | **Base case model** | **Exclusion of high RoB studies** | **Exclusion of small studies (<100 patients)** | **Exclusion of type 1 DM studies** | **Exclusion of normotension studies** | **Adjusted for publication year** | **Adjusted for mean age** | **Adjusted for % males** | **Adjusted for control group risk** | **Rate ratios based on patient-years** |
| --- | --- | --- | --- | --- | --- | --- | --- | --- | --- | --- |
| **ACEi** | reference | reference | reference | reference | reference | reference | reference | reference | reference | reference |
| **BB** | 1.12 (0.78-1.94) | 1.23 (0.79-1.95) | 1.23 (0.78-1.95) | 1.23 (0.78-1.97) | 1.24 (0.78-1.98) | 1.23 (0.78-1.98) | 1.23 (0.79-1.98) | 1.22 (0.78-1.96) | 1.24 (0.79-1.96) | 1.23 (0.80-1.90) |
| **CCB** | 0.94 (0.78-1.22) | 0.96 (0.79-1.25) | 0.95 (0.78-1.23) | 0.95 (0.78-1.23) | 0.96 (0.78-1.26) | 0.95 (0.78-1.24) | 0.95 (0.78-1.25) | 0.95 (0.78-1.24) | 0.94 (0.78-1.22) | 0.94 (0.79-1.20) |
| **ARB** | 1.14 (0.98-1.37) | 1.14 (0.98-1.38) | 1.14 (0.97-1.37) | 1.14 (0.98-1.38) | 1.15 (0.98-1.41) | 1.15 (0.97-1.41) | 1.15 (0.98-1.41) | 1.13 (0.95-1.38) | 1.15 (0.98-1.40) | 1.11 (0.97-1.32) |
| **ACEi+CCB** | NA | NA | NA | NA | NA | NA | NA | NA | NA | NA |
| **Diuretic** | 0.96 (0.74-1.34) | 0.96 (0.74-1.37) | 0.96 (0.74-1.36) | 0.96 (0.74-1.36) | 0.96 (0.74-1.40) | 0.96 (0.73-1.39) | 0.96 (0.73-1.38) | 0.96 (0.73-1.37) | 0.96 (0.74-1.35) | 0.96 (0.75-1.31) |
| **ACEi+diuretic** | NA | NA | NA | NA | NA | NA | NA | NA | NA | NA |
| **ARB+diuretic** | 9.81 (0.27-564) | 10.36 (0.22-1678) | 6.97 (0.11-1316) | 7.58 (0.14-2812) | 9.23 (0.15-1066) | 6.67 (0.20-1585) | 11.49 (0.19-1429) | 6.17 (0.18-609) | 8.18 (0.25-941) | 5.73 (0.14-294) |
| **ACEi+ARB** | 1.00 (0.82-1.23) | 1.01 (0.82-1.24) | 1.00 (0.82-1.23) | 1.01 (0.82-1.23) | 1.04 (0.84-1.34) | 1.01 (0.81-1.25) | 1.01 (0.82-1.25) | 1.00 (0.81-1.23) | 1.01 (0.82-1.24) | 1.00 (0.83-1.20) |
| **DRi+ACEi** | NA | NA | NA | NA | NA | NA | NA | NA | NA | NA |
| **DRi+ARB** | NA | NA | NA | NA | NA | NA | NA | NA | NA | NA |
| **DRi+diuretic** | 1.32 (0.56-3.17) | 1.32 (0.58-3.15) | 1.28 (0.56-3.12) | 1.31 (0.56-3.20) | 1.31 (0.57-3.19) | 1.32 (0.54-3.24) | 1.31 (0.55-3.21) | 1.30 (0.55-3.15) | 1.30 (0.55-3.20) | 1.26 (0.58-2.99) |
| **ARB+CCB** | 3.25 (0.28-89.45) | 3.20 (0.25-115.20) | 2.68 (0.17-94.99) | 2.87 (0.17-185.70) | 3.34 (0.20-128.70) | 2.55 (0.20-149.20) | 3.43 (0.21-123.90) | 2.60 (0.22-60.48) | 2.90 (0.25-78.76) | 2.49 (0.19-64.50) |
|  |  |  |  |  |  |  |  |  |  |  |
| **Residual deviance** | 73.23 | 69.17 | 70.40 | 66.34 | 63.52 | 73.72 | 73.40 | 73.66 | 72.65 | 73.18 |
| **DIC** | 407.85 | 400.46 | 402.44 | 374.39 | 358.87 | 409.71 | 409.95 | 409.50 | 408.04 | 411.38 |
| **# data points** | 66 | 64 | 64 | 58 | 66 | 66 | 66 | 66 | 66 | 66 |
| **SD** | 0.11 (0.01-0.27) | 0.12 (0.02-0.28) | 0.12 (0.01-0.28) | 0.12 (0.01-0.28) | 0.12 (0.01-0.30) | 0.12 (0.01-0.29) | 0.12 (0.02-0.29) | 0.12 (0.01-0.29) | 0.12 (0.01-0.28) | 0.10 (0.02-0.25) |

Values represent odds ratios with 95% credible intervals. Values for renin-angiotensin blockers (alone or in combination) are highlighted in dark green. RoB = risk of bias. DIC = deviance information criteria. SD = standard deviation.

ACEi = Angiotensin converting enzyme inhibitor; BB = beta-blocker; CCB = Calcium channel blocker; ARB = Angiotensin receptor blocker; ACEi+CCB = Angiotensin converting enzyme inhibitor + calcium channel blocker; ACEi+CCB = Angiotensin converting enzyme inhibitor + diuretic; ACEi+ARB = Angiotensin converting enzyme inhibitor + angiotensin receptor blocker; DRi+ACEi = Direct renin inhibitor (aliskiren) + angiotensin converting enzyme inhibitor; DRi+ARB = Direct renin inhibitor (aliskiren) + angiotensin receptor blocker; DRi+diuretic = Direct renin inhibitor (aliskiren) + diuretic.

**Table 10j. Sensitivity analyses. Heart failure.**

|  | **Base case model** | **Exclusion of high RoB studies** | **Exclusion of small studies (<100 patients)** | **Exclusion of type 1 DM studies** | **Exclusion of normotension studies** | **Adjusted for publication year** | **Adjusted for mean age** | **Adjusted for % males** | **Adjusted for control group risk** | **Rate ratios based on patient-years** |
| --- | --- | --- | --- | --- | --- | --- | --- | --- | --- | --- |
| **ACEi** | reference | reference | reference | reference | reference | reference | reference | reference | reference | reference |
| **BB** | 1.45 (0.92-2.27) | 1.44 (0.91-2.24) | 1.44 (0.90-2.26) | 1.43 (0.90-2.25) | 1.45 (0.91-2.35) | 1.43 (0.92-2.22) | 1.45 (0.92-2.30) | 1.42 (0.89-2.27) | 1.45 (0.91-2.35) | 1.42 (0.91-2.13) |
| **CCB** | 1.29 (1.08-1.54) | 1.29 (1.08-1.53) | 1.29 (1.08-1.56) | 1.29 (1.08-1.55) | 1.29 (1.08-1.55) | 1.29 (1.09-1.55) | 1.29 (1.09-1.53) | 1.29 (1.07-1.55) | 1.29 (1.08-1.55) | 1.27 (1.07-1.48) |
| **ARB** | 0.99 (0.86-1.12) | 0.99 (0.86-1.23) | 1.00 (0.85-1.14) | 0.99 (0.86-1.13) | 0.99 (0.85-1.13) | 0.98 (0.85-1.12) | 1.00 (0.87-1.13) | 0.99 (0.85-1.13) | 0.99 (0.85-1.13) | 1.00 (0.87-1.12) |
| **ACEi+CCB** | NA | NA | NA | NA | NA | NA | NA | NA | NA | NA |
| **Diuretic** | 0.94 (0.72-1.23) | 0.94 (0.72-1.24) | 0.94 (0.71-1.27) | 0.94 (0.71-1.24) | 0.94 (0.71-1.24) | 0.94 (0.73-1.23) | 0.94 (0.73-1.22) | 0.94 (0.71-1.25) | 0.94 (0.71-1.24) | 0.95 (0.75-1.20) |
| **ACEi+diuretic** | 2.02 (0.22-22.89) | 2.03 (0.24-22.35) | NA | 2.32 (0.21-27.08) | 1.95 (0.21-15.40) | 1.77 (0.19-14.76) | 2.02 (0.20-20.54) | 1.71 (0.17-15.99) | 1.95 (0.21-15.40) | 1.85 (0.21-14.19) |
| **ARB+diuretic** | 0.37 (0.05-2.43) | 0.38 (0.05-2.65) | 0.37 (0.05-2.11) | 0.35 (0.06-2.29) | 0.36 (0.04-2.42) | 0.35 (0.04-2.36) | 0.38 (0.04-2.51) | 0.42 (0.05-2.76) | 0.36 (0.04-2.42) | 0.40 (0.05-2.54) |
| **ACEi+ARB** | 0.86 (0.73-1.01) | 0.86 (0.73-1.01) | 0.87 (0.72-1.03) | 0.86 (0.73-1.01) | 0.86 (0.73-1.02) | 0.86 (0.73-1.00) | 0.87 (0.74-1.01) | 0.86 (0.72-1.02) | 0.86 (0.73-1.02) | 0.89 (0.76-1.02) |
| **DRi+ACEi** | 1.06 (0.75-1.45) | 1.06 (0.76-1.45) | 0.95 (0.72-1.24) | 1.06 (0.76-1.45) | 1.06 (0.75-1.45) | 1.05 (0.75-1.43) | 1.06 (0.76-1.44) | 1.05 (0.75-1.46) | 1.06 (0.75-1.45) | 1.05 (078-1.41) |
| **DRi+ARB** | 0.83 (0.59-1.14) | 0.83 (0.60-1.13) | 0.80 (0.57-1.11) | 0.82 (0.59-1.14) | 0.83 (0.59-1.15) | 0.83 (0.60-1.13) | 0.84 (0.60-1.14) | 0.83 (0.59-1.15) | 0.83 (0.59-1.15) | 0.85 (0.61-1.15) |
| **DRi+diuretic** | 1.05 (0.65-1.70) | 1.05 (0.65-1.70) | 1.06 (0.64-1.75) | 1.04 (0.64-1.71) | 1.04 (0.64-1.71) | 1.05 (0.66-1.69) | 1.05 (0.66-1.67) | 1.04 (0.64-1.72) | 1.04 (0.64-1.71) | 1.02 (0.67-1.53) |
| **ARB+CCB** | 0.55 (0.10-2.23) | 0.52 (0.11-2.42) | 0.55 (0.10-2.13) | 0.52 (0.11-2.17) | 0.54 (0.09-2.45) | 0.52 (0.09-2.24) | 0.54 (0.09-2.28) | 0.57 (0.11-2.54) | 0.54 (0.09-2.45) | 0.56 (0.13-2.69) |
|  |  |  |  |  |  |  |  |  |  |  |
| **Residual deviance** | 90.36 | 88.22 | 85.26 | 88.03 | 91.48 | 90.91 | 90.55 | 90.71 | 91.48 | 90.09 |
| **DIC** | 510.94 | 505.60 | 499.43 | 505.87 | 512.92 | 511.67 | 511.46 | 512.49 | 512.92 | 515.62 |
| **# data points** | 76 | 74 | 73 | 74 | 70 | 76 | 76 | 76 | 76 | 76 |
| **SD** | 0.10 (0.01-0.25) | 0.09 (0.00-0.24) | 0.11 (0.01-0.26) | 0.10 (0.00-0.25) | 0.09 (0.01-0.26) | 0.09 (0.00-0.24) | 0.08 (0.00-0.23) | 0.10 (0.01-0.26) | 0.09 (0.00-0.26) | 0.08 (0.01-0.21) |

Values represent odds ratios with 95% credible intervals. Values for renin-angiotensin blockers (alone or in combination) are highlighted in dark green. RoB = risk of bias. DIC = deviance information criteria. SD = standard deviation.

ACEi = Angiotensin converting enzyme inhibitor; BB = beta-blocker; CCB = Calcium channel blocker; ARB = Angiotensin receptor blocker; ACEi+CCB = Angiotensin converting enzyme inhibitor + calcium channel blocker; ACEi+CCB = Angiotensin converting enzyme inhibitor + diuretic; ACEi+ARB = Angiotensin converting enzyme inhibitor + angiotensin receptor blocker; DRi+ACEi = Direct renin inhibitor (aliskiren) + angiotensin converting enzyme inhibitor; DRi+ARB = Direct renin inhibitor (aliskiren) + angiotensin receptor blocker; DRi+diuretic = Direct renin inhibitor (aliskiren) + diuretic.
