# Supplementary material for: Cardiovascular and Renal Outcomes of Renin–Angiotensin System Blockade in Adult Patients with Diabetes Mellitus: A Systematic Review with Network Meta-Analyses
Source: PLoS Med. 2016 Mar 8;13(3):e1001971. doi: 10.1371/journal.pmed.1001971 (PMC4783064; doi:10.1371/journal.pmed.1001971)
Supplement: S11 Table — (DOCX) [file pmed.1001971.s014.docx]

**S11 Table. Summary of model fit statistics from network meta-analysis by outcome.**

|  | **Major CV outcome** | **CV mortality** | **Myocardial infarction** | **Stroke** | **Progression of renal disease** | **ESRD** | **Doubling CrS** | **All-cause mortality** | **Angina pectoris** | **Heart failure** |
| --- | --- | --- | --- | --- | --- | --- | --- | --- | --- | --- |
| **Consistency model** | | | | | | | | | | |
| **Number of data points** | 76 | 96 | 104 | 95 | 44 | 52 | 56 | 135 | 66 | 76 |
| **Residual deviance (posterior mean)** | 82.70 | 107.40 | 125.60 | 96.40 | 44.51 | 56.79 | 57.83 | 148.10 | 73.23 | 90.36 |
| **SD (posterior median) and 95% credible intervales** | 0.13 (0.05-0.25) | 0.21 (0.09-0.40) | 0.18 (0.01-0.36) | 0.06 (0.00-0.20) | 0.18 (0.07-0.36) | 0.16 (0.01-0.43) | 0.26 (0.08-0.51) | 0.11 (0.03-0.22) | 0.11 (0.01-0.27) | 0.10 (0.01-0.25) |
| **DIC** | 579.25 | 577.66 | 618.75 | 581.58 | 344.58 | 305.76 | 371.68 | 784.78 | 407.85 | 510.94 |
| **Inconsistency model** | | | | | | | | | | |
| **Number of data points** | 76 | 96 | 104 | 95 | 44 | 52 | 56 | 135 | 66 | 76 |
| **Residual deviance (posterior mean)** | 82.61 | 105.30 | 122.20 | 97.20 | 44.47 | 55.97 | 57.72 | 147.60 | 74.99 | 88.84 |
| **SD (posterior median) and 95% credible intervales** | 0.13 (0.04-0.26) | 0.16 (0.03-0.34) | 0.19 (0.03-0.41) | 0.09 (0.01-0.25) | 0.21 (0.06-0.49) | 0.19 (0.01-0.67) | 0.23 (0.02-0.56) | 0.10 (0.01-0.20) | 0.14 (0.01-0.35) | 0.16 (0.02-0.36) |
| **DIC** | 581.65 | 576.11 | 620.50 | 589.80 | 347.09 | 308.78 | 372.74 | 788.92 | 414.21 | 516.81 |

CV = cardiovascular. CrS = serum creatinine. DIC = deviance information criteria. ESRD = end-stage renal disease. SD = standard deviation.

Statistical note: Goodness of fit was measured using the posterior mean of the residual deviance and the deviance information criterion (DIC). In a well fitting model the posterior mean residual deviance should be close to the number of data points. Consistency between the different sources of evidence was explored statistically by comparing the fit of a model assuming consistency with a model which allowed for inconsistency. If the inconsistency model had the smallest posterior mean residual deviance, or deviance information criterion (DIC) value then this indicates potential inconsistency in the data. Differences of ≥ 5 points for deviance information criterion (DIC) were considered meaningful.
