# Supplementary material for: Cardiovascular and Renal Outcomes of Renin–Angiotensin System Blockade in Adult Patients with Diabetes Mellitus: A Systematic Review with Network Meta-Analyses
Source: PLoS Med. 2016 Mar 8;13(3):e1001971. doi: 10.1371/journal.pmed.1001971 (PMC4783064; doi:10.1371/journal.pmed.1001971)
Supplement: S12 Table — (DOCX) [file pmed.1001971.s015.docx]

**S12 Table. Methodological differences with previous reviews of cardiovascular and/or renal outcomes of RAS blockade in diabetic patients**

| **Characteristic** | **Present study** | **Cheng et al 2014** | **Wu et al 2013** | **Vejakama et al 2012** | **Nakao et al 2012** |
| --- | --- | --- | --- | --- | --- |
| Design | Systematic review and network meta-analysis | Systematic review and meta-analysis | Systematic review and network meta-analysis | Systematic review and network meta-analysis | Systematic review and meta-analysis |
| Interventions | ACEi, ARBs, DRi (aliskiren) and multiple combinations (e.g. ACEi combinations; ARBs combinations; DRi combinations) | ACEi and ARBs | ACEi, ARBs and multiple combinations (e.g. only ACEi combinations) | RAS blockers (ACEi/ARBs) | RAS blockers (ACEi/ARBs) |
| Number of studies (references) | 71 trials (88 references) | 35 trials (34 references) | 63 trials (67 references) | 28 trials (28 references) | 19 trials (17 references) |
| Last search (in PubMed) | September 2014 | December 2012 | December 2011 | July 2011 | June 2010 |
| Number of patients with diabetes mellitus | 103,120 | 56,444 | 36,917 | 17,939 | 44,639 |
| Outcomes (number of events) | All-cause mortality (11,199)  CV composite (12,328)  Renal composite (9,267)  CV mortality (6,166)  Myocardial infarction (4,593)  Stroke (4,591)  Angina (5,026)  Heart failure (5,272)  Doubling of CrS (2,645)  ESRD (1,786) | All cause-mortality (3,690)  CV mortality (not reported)  Myocardial infarction (2,359)  Stroke (2,839)  Heart failure (1,353)  Major CV event (7,995) | All-cause mortality (2,393)  Doubling of CrS (1,099)  ESRD (766) | Doubling of CrS (1,166)  ESRD (707)  Major microvascular complications (971)  Macroalbuminuria (445)  Microalbuminuria (916)  Albuminuria regression (916) | CV mortality (1,473)  Myocardial infarction (2,479)  Stroke (2,310)  MACE (6,278)  Treatment discontinuation (7,365) |
| Inclusion of trials with a sample size > 1000 diabetic patients | 24 trials: Micro-HOPE, RENAAL, IDNT*, Val-HeFT*, LIFE, VALIANT*, VALUE*, BENEDICT, DIABHYCAR, EUROPA/PERSUADE*, ALLHAT*, ADVANCE, DIRECT Prevent-1*, DIRECT Protect-1*, DIRECT Protect-2*, PRoFESS*, ONTARGET*, TRANSCEND*, ROADMAP*, CASE-J*, ALTITUDE*, NAGOYA-HEART, VA NEPHRON-D, COLM*  *Trials contributing with both published and unpublished data. | 14 trials: Micro-HOPE, RENAAL, IDNT, LIFE, DIABHYCAR, EUROPA/PERSUADE, ALLHAT, ADVANCE, DIRECT Prevent-1, DIRECT Protect-1, DIRECT Protect-2, PRoFESS, ROADMAP, CASE-J | 12 trials: IDNT, RENAAL, BENEDICT, DIABHYCAR, ADVANCE, EUROPA/PERSUADE, ALLHAT, ADVANCE, DIRECT Prevent-1, DIRECT Protect-1, DIRECT Protect-2, ROADMAP | 6 trials: Micro-HOPE, RENAAL, IDNT, DIABHYCAR, DIRECT Protect-2, ROADMAP | 10 trials: Micro-HOPE, RENAAL, IDNT, LIFE, DIABHYCAR, EUROPA/PERSUADE, ALLHAT, PRoFESS, ROADMAP, CASE-J |
| Conclusions related to comparative effects of RAS blockers | ‘In adults with diabetes, comparisons of different RAS blockers showed similar effects of ACEi and ARB on major cardiovascular and renal outcomes. Compared with monotherapy, combination of ACEi and ARB failed to provide significant benefits on major outcomes. Clinicians should discuss the balance between benefits, costs, and harms with the individual patient before starting treatment options.’ | ‘ACEi reduced all-cause mortality, CV mortality, and major CV events in patients with diabetes, whereas ARBs had no benefits on these outcomes. Thus, ACEi should be considered as first-line therapy to limit excess mortality and morbidity in this population.’ | ‘Our analyses show the renoprotective effects and superiority of using ACEi in patients with diabetes, and available evidence is not able to show a better effect for ARBs compared with ACEi. (…) Calcium channel blockers might be the preferred treatment in combination with ACEi if adequate blood pressure control cannot be achieved by ACEi alone.’ | ‘Our review suggests a consistent reno-protective effect of ACEi/ARB over other antihypertensive drugs, mainly [calcium channel blockers] CCBs, and placebo in type 2 diabetes. The lack of any differences in BP decrease between ACEi/ARB and active comparators suggest this benefit is not due simply to the antihypertensive effect.’ | ‘The available evidence shows that treatment with RAS blockade can routinely be considered for diabetic patients to reduce major cardiovascular events.’ |

**References**

1. Cheng J, Zhang W, Zhang X, Han F, Li X, He X, Li Q, Chen J. Effect of angiotensin-converting enzyme inhibitors and angiotensin II receptor blockers on all-cause mortality, cardiovascular deaths, and cardiovascular events in patients with diabetes mellitus: a meta-analysis. JAMA Intern Med. 2014;174(5):773-85. PubMed PMID: 24687000
2. Wu HY, Huang JW, Lin HJ, Liao WC, Peng YS, Hung KY, Wu KD, Tu YK, Chien KL. Comparative effectiveness of renin-angiotensin system blockers and other antihypertensive drugs in patients with diabetes: systematic review and bayesian network meta-analysis. BMJ. 2013;347:f6008. PubMed PMID: 24157497.
3. Vejakama P, Thakkinstian A, Lertrattananon D, Ingsathit A, Ngarmukos C, Attia J. Reno-protective effects of renin-angiotensin system blockade in type 2 diabetic patients: a systematic review and network meta-analysis. Diabetologia. 2012;55(3):566-78. PubMed PMID: 22189484.
4. Nakao YM, Teramukai S, Tanaka S, Yasuno S, Fujimoto A, Kasahara M, Ueshima K, Nakao K, Hinotsu S, Nakao K, Kawakami K. Effects of renin-angiotensin system blockades on cardiovascular outcomes in patients with diabetes mellitus: A systematic review and meta-analysis. Diabetes Res Clin Pract. 2012;96(1):68-75. PubMed PMID: 22197527.
