# Supplementary material for: Cardiovascular and Renal Outcomes of Renin–Angiotensin System Blockade in Adult Patients with Diabetes Mellitus: A Systematic Review with Network Meta-Analyses
Source: PLoS Med. 2016 Mar 8;13(3):e1001971. doi: 10.1371/journal.pmed.1001971 (PMC4783064; doi:10.1371/journal.pmed.1001971)
Supplement: S13 Table — (DOCX) [file pmed.1001971.s016.docx]

**S13 Table. Randomized controlled trials included in our systematic review versus previous reviews**

| **Trial name, year** | **Cheng et al 2014** | **Wu et al 2013** | **Vejakama et al 2012** | **Nakao et al 2012** | **No. of patients** |
| --- | --- | --- | --- | --- | --- |
| Parving et al 1989 | Yes | Yes | No | No | 32 |
| Bauer et al 1992 | Yes | Yes | No | No | 33 |
| Björck et al 1992 | No | Yes | No | No | 40 |
| Chan et al 1992 | No | Yes | No | No | 102 |
| Lacourcière et al 1993 | No | No | Yes | No | 109 |
| Lewis et al 1993 | Yes | Yes | No | No | 409 |
| Ravid et al 1993 | No | Yes | Yes | No | 108 |
| Elving et al 1994 | No | Yes | No | No | 30 |
| Sano et al 1994 | Yes | Yes | No | No | 52 |
| Laffel et al 1995 | Yes | Yes | No | No | 143 |
| Bakris et al 1996 | Yes | Yes | Yes | No | 52 |
| Viberti et al 1996 | No | Yes | No | No | 235 |
| Nielsen et al 1997 | Yes | Yes | Yes | No | 43 |
| ABCD-Hypertension 1998 | Yes | Yes | No | Yes | 470 |
| ABCD-normo 2002 | No | No | No | Yes | 480 |
| Crepaldi et al 1998 | No | Yes | No | No | 92 |
| FACET 1998 | Yes | Yes | No | Yes | 380 |
| Nankervis et al 1998 | Yes | Yes | No | No | 40 |
| Ravid et al 1998 | Yes | Yes | Yes | No | 194 |
| UKPDS-39 1998 | Yes | Yes | Yes | Yes | 758 |
| Fogari et al 1999 | No | Yes | Yes | No | 107 |
| ATLANTIS 2000 | No | Yes | No | No | 140 |
| Tarnow et al 2000 | No | Yes | No | No | 52 |
| Chan et al 2000 | No | No | Yes | No | 102 |
| STOP Hypertension-2 2000 | Yes | No | No | Yes | 719 |
| Micro-HOPE 2000 | Yes | No | Yes | Yes | 3577 |
| J-MIND 2001 | Yes | No | No | No | 436 |
| IDNT 2001^a^ | Yes | Yes | Yes | Yes | 1715 |
| IRMA-2 2001 ^a^ | Yes | Yes | No | No | 608 |
| Jerums et al 2001 | No | Yes | No | No | 42 |
| RENAAL 2001 | Yes | Yes | Yes | Yes | 1513 |
| CAPPP 2001 | Yes | No | No | Yes | 572 |
| **Val-HeFT 2001 ^a^** | **No** | **No** | **No** | **No** | **1185** |
| Fogari et al 2002 | Yes | Yes | Yes | No | 309 |
| JAPAN-IDDM 2002 | No | Yes | No | No | 79 |
| LIFE 2002 | Yes | No | No | Yes | 1195 |
| **VALIANT 2003 ^a^** | **No** | **No** | **No** | **No** | **3400** |
| **VALUE 2004 ^a^** | **No** | **No** | **No** | **No** | **4823** |
| BENEDICT 2004 | No | Yes | Yes | No | 1204 |
| DETAIL 2004 | Yes | Yes | No | No | 250 |
| DIABHYCAR 2004 | Yes | Yes | Yes | Yes | 4912 |
| NESTOR 2004 | No | Yes | Yes | No | 570 |
| JMIC-B 2004 | Yes | No | No | Yes | 372 |
| Ko et al 2005 | No | Yes | No | No | 42 |
| Schram et al 2005 | No | Yes | No | No | 70 |
| PERSUADE 2005 | Yes | No | No | Yes | 1502 |
| ALLHAT 2005 ^a^ | Yes | No | No | Yes | 13168 |
| SCOPE 2005 | Yes | No | No | Yes | 599 |
| ABCD-2V 2006 | No | Yes | No | No | 129 |
| Tong et al 2006 | No | Yes | No | No | 38 |
| ADVANCE 2007 | Yes | Yes | No | No | 11140 |
| DIRECT-Prevent 1 2008 ^a^ | Yes | Yes | No | No | 1421 |
| DIRECT-Protect 1 2008 ^a^ | Yes | Yes | No | No | 1905 |
| DIRECT-Protect 2 2008 ^a^ | Yes | Yes | Yes | No | 1905 |
| GUARD 2008 | No | Yes | No | No | 332 |
| PRoFESS 2008 ^a^ | Yes | No | No | Yes | 5743 |
| **ONTARGET 2008 ^a^** | **No** | **No** | **No** | **No** | **9612** |
| **TRANSCEND 2008 ^a^** | **No** | **No** | **No** | **No** | **2118** |
| Kohlmann Jr et al 2009 | No | Yes | No | No | 110 |
| Mehdi et al 2009 | No | Yes | No | No | 80 |
| RAAS 2009 | No | Yes | No | No | 285 |
| CASE-J 2010 ^a^ | Yes | No | No | Yes | 2018 |
| ROADMAP 2011 ^a^ | No | Yes | Yes | Yes | 4447 |
| ORIENT 2011 | Yes | No | No | Yes | 563 |
| **DEMAND 2011** | **No** | **No** | **No** | **No** | **380** |
| **ALTITUDE 2012 ^a^** | **No** | **No** | **No** | **No** | **8579** |
| **NAGOYA HEART 2012** | **No** | **No** | **No** | **No** | **1150** |
| **VA NEPHRON-D 2013** | **No** | **No** | **No** | **No** | **1448** |
| **ASTRONAUT 2013 ^a^** | **No** | **No** | **No** | **No** | **662** |
| **COLM 2014 ^a^** | **No** | **No** | **No** | **No** | **1362** |
| **OSCAR 2014 ^a^** | **No** | **No** | **No** | **No** | **628** |
