# Supplementary material for: Cardiovascular and Renal Outcomes of Renin–Angiotensin System Blockade in Adult Patients with Diabetes Mellitus: A Systematic Review with Network Meta-Analyses
Source: PLoS Med. 2016 Mar 8;13(3):e1001971. doi: 10.1371/journal.pmed.1001971 (PMC4783064; doi:10.1371/journal.pmed.1001971)
Supplement: S14 Table — (DOCX) [file pmed.1001971.s017.docx]

**S14 Table. Randomized controlled trials excluded in our systematic review that were included in previous reviews.**

| **Trial name, year** | **Cheng et al 2014** | **Wu et al 2013** | **Vejakama et al 2012** | **Nakao et al 2012** | **No. of patients** | **Reason for exclusion in our systematic review** |
| --- | --- | --- | --- | --- | --- | --- |
| Chase et al 1993 | No | Yes | No | No | 16 | No outcome data (all-cause mortality with zero events) |
| Hallab et al 1993 | No | Yes | No | No | 25 | No outcome data (all-cause mortality with zero events) |
| O’Donnell et al 1993 | No | Yes | No | No | 32 | No outcome data (all-cause mortality with zero events) |
| Bakris et al 1994 | No | Yes | No | No | 15 | No outcome data (all-cause mortality with zero events) |
| Capek et al 1994 | No | Yes | No | No | 20 | No outcome data (all-cause mortality, ESRD and doubling CrS with zero events) |
| Lebovitz et al 1994 | No | No | Yes | No | 68 | No relevant outcome (macroalbuminuria) |
| Mosconi et al 1996 | No | No | Yes | No | 13 | No relevant outcome (albuminuria regression) |
| Ahmad et al 1997 | No | No | Yes | No | 103 | No relevant outcome (macroalbuminuria) |
| Bakris et al 1998 | No | Yes | No | No | 44 | No outcome data (all-cause mortality with zero events) |
| Garg et al 1998 | No | Yes | No | No | 11 | No outcome data (all-cause mortality with zero events) |
| Mathiesen et al 1999 | No | Yes | No | No | 44 | No outcome data (all-cause mortality with zero events) |
| Shiba et al 2000 | No | No | Yes | No | 39 | No relevant outcome (macroalbuminuria) |
| Estacio et al 2000 | No |  | Yes |  | 230 | No relevant outcome (microalbuminuria, macroalbuminuria) |
| Lacourcière et al 2000 | No | Yes | No | No | 103 | No outcome data (all-cause mortality with zero events) |
| Bojestig et al 2001 | No | Yes | No | No | 55 | No outcome data (all-cause mortality with zero events) |
| Deerochanawong et al 2001 | No | Yes | No | No | 60 | No outcome data (all-cause mortality with zero events) |
| ESPRIT 2001 | No | Yes | No | No | 54 | No outcome data (all-cause mortality with zero events) |
| Kopft et al 2001 | No | Yes | No | No | 46 | No outcome data (all-cause mortality with zero events) |
| Kventy et al 2001 | No | Yes | No | No | 89 | No outcome data (all-cause mortality with zero events) |
| Baba et al 2001 | No | No | Yes | No | 117 | No relevant outcome (microalbuminuria, macroalbuminuria, albuminuria regression) |
| Jerums et al 2004 | No | No | Yes | No | 22 | No relevant outcome (macroalbuminuria, albuminuria regression) |
| Rizzoni et al 2005 | No | Yes | No | No | 15 | No outcome data (all-cause mortality with zero events) |
| Fogari et al 2005 | No | No | Yes | No | 99 | No relevant outcome (albuminuria regression) |
| Katayama et al 2006 | No | Yes | No | No | 87 | No outcome data (all-cause mortality with zero events) |
| Ogawa et al 2007 | No | No | Yes | No | 52 | No relevant outcome (albuminuria regression) |
| Kashiwagi et al 2007 | No | No | Yes | No | 150 | No relevant outcome (albuminuria regression)  Follow-up < 1 year |
| Perrin et al 2008 | No | Yes | No | No | 13 | No outcome data (all-cause mortality with zero events) |
| MITEC 2009 | No | Yes | No | No | 209 | No outcome data (all-cause mortality with zero events) |
