# Supplementary material for: Cardiovascular and Renal Outcomes of Renin–Angiotensin System Blockade in Adult Patients with Diabetes Mellitus: A Systematic Review with Network Meta-Analyses
Source: PLoS Med. 2016 Mar 8;13(3):e1001971. doi: 10.1371/journal.pmed.1001971 (PMC4783064; doi:10.1371/journal.pmed.1001971)
Supplement: S1 Text — (DOCX) [file pmed.1001971.s018.docx]

**S1 Text. PubMed search terms.**

(("renin-angiotensin system"[MeSH Terms] OR ("renin-angiotensin"[All Fields] AND "system"[All Fields]) OR "renin-angiotensin system"[All Fields] OR ("renin"[All Fields] AND "angiotensin"[All Fields] AND "system"[All Fields]) OR "renin angiotensin system"[All Fields]) OR ("renin-angiotensin system"[MeSH Terms] OR ("renin-angiotensin"[All Fields] AND "system"[All Fields]) OR "renin-angiotensin system"[All Fields] OR ("renin"[All Fields] AND "angiotensin"[All Fields] AND "aldosterone"[All Fields] AND "system"[All Fields]) OR "renin angiotensin aldosterone system"[All Fields]) OR ("angiotensin-converting enzyme inhibitors"[Pharmacological Action] OR "angiotensin-converting enzyme inhibitors"[MeSH Terms] OR ("angiotensin-converting"[All Fields] AND "enzyme"[All Fields] AND "inhibitors"[All Fields]) OR "angiotensin-converting enzyme inhibitors"[All Fields] OR ("angiotensin"[All Fields] AND "converting"[All Fields] AND "enzyme"[All Fields] AND "inhibitor"[All Fields]) OR "angiotensin converting enzyme inhibitor"[All Fields]) OR (("receptors, angiotensin"[MeSH Terms] OR ("receptors"[All Fields] AND "angiotensin"[All Fields]) OR "angiotensin receptors"[All Fields] OR ("angiotensin"[All Fields] AND "receptor"[All Fields]) OR "angiotensin receptor"[All Fields]) AND blocker[All Fields]) OR (("renin"[MeSH Terms] OR "renin"[All Fields]) AND inhibitor[All Fields]) OR (direct[All Fields] AND ("renin"[MeSH Terms] OR "renin"[All Fields]) AND inhibitor[All Fields]) OR ("benazepril"[Supplementary Concept] OR "benazepril"[All Fields]) OR ("captopril"[MeSH Terms] OR "captopril"[All Fields]) OR ("enalapril"[MeSH Terms] OR "enalapril"[All Fields]) OR ("cilazapril"[MeSH Terms] OR "cilazapril"[All Fields]) OR ("delapril"[Supplementary Concept] OR "delapril"[All Fields]) OR ("fosinopril"[MeSH Terms] OR "fosinopril"[All Fields]) OR ("imidapril"[Supplementary Concept] OR "imidapril"[All Fields]) OR (("lisinopril"[MeSH Terms] OR "lisinopril"[All Fields]) AND ("moexipril"[Supplementary Concept] OR "moexipril"[All Fields])) OR (("perindopril"[MeSH Terms] OR "perindopril"[All Fields]) AND ("quinapril"[Supplementary Concept] OR "quinapril"[All Fields])) OR ("ramipril"[MeSH Terms] OR "ramipril"[All Fields]) OR ("spirapril"[Supplementary Concept] OR "spirapril"[All Fields]) OR ("temocapril hydrochloride"[Supplementary Concept] OR "temocapril hydrochloride"[All Fields] OR "temocapril"[All Fields]) OR ("trandolapril"[Supplementary Concept] OR "trandolapril"[All Fields]) OR ("zofenopril"[Supplementary Concept] OR "zofenopril"[All Fields]) OR ("candesartan"[Supplementary Concept] OR "candesartan"[All Fields]) OR ("irbesartan"[Supplementary Concept] OR "irbesartan"[All Fields]) OR ("losartan"[MeSH Terms] OR "losartan"[All Fields]) OR ("telmisartan"[Supplementary Concept] OR "telmisartan"[All Fields]) OR ("valsartan"[Supplementary Concept] OR "valsartan"[All Fields]) OR ("olmesartan"[Supplementary Concept] OR "olmesartan"[All Fields]) OR ("eprosartan"[Supplementary Concept] OR "eprosartan"[All Fields]) OR ("azilsartan"[Supplementary Concept] OR "azilsartan"[All Fields]) OR ("aliskiren"[Supplementary Concept] OR "aliskiren"[All Fields])) AND (("review literature as topic"[MeSH Terms] OR "systematic review"[All Fields]) OR ("meta-analysis"[Publication Type] OR "meta-analysis as topic"[MeSH Terms] OR "meta-analysis"[All Fields])) AND (renal[All Fields] OR (renoprotecting[All Fields] OR renoprotection[All Fields] OR renoprotectiv[All Fields] OR renoprotective[All Fields] OR renoprotective'[All Fields]) OR (cardiovascu[All Fields] OR cardiovascuaire[All Fields] OR cardiovascualar[All Fields] OR cardiovascualr[All Fields] OR cardiovascuar[All Fields] OR cardiovascugratedar[All Fields] OR cardiovascuire[All Fields] OR cardiovascul[All Fields] OR cardiovascula[All Fields] OR cardiovasculae[All Fields] OR cardiovasculaere[All Fields] OR cardiovasculair[All Fields] OR cardiovasculaire[All Fields] OR cardiovasculaire'[All Fields] OR cardiovasculaires[All Fields] OR cardiovasculaires'[All Fields] OR cardiovasculairs[All Fields] OR cardiovasculairs'[All Fields] OR cardiovasculan[All Fields] OR cardio-renal[All Fields] OR ("cardiovascular system"[MeSH Terms] OR ("cardiovascular"[All Fields] AND "system"[All Fields]) OR "cardiovascular system"[All Fields] OR "cardiovascular"[All Fields]) OR cardiovascular'[All Fields] OR cardiovascular's[All Fields] OR cardiovascular,[All Fields] OR cardiovasculara[All Fields] OR cardiovascularand[All Fields] OR cardiovascularcare[All Fields] OR cardiovascularcause[All Fields] OR cardiovasculardisease[All Fields] OR cardiovasculardiseases[All Fields] OR cardiovasculardysfunction[All Fields] OR cardiovasculare[All Fields] OR cardiovasculared[All Fields] OR cardiovascularem[All Fields] OR cardiovascularen[All Fields] OR cardiovascularer[All Fields] OR cardiovasculares[All Fields] OR cardiovascularevents[All Fields] OR cardiovasculari[All Fields] OR cardiovascularie[All Fields] OR cardiovascularies[All Fields] OR cardiovascularily[All Fields] OR cardiovascularire[All Fields] OR cardiovascularires[All Fields] OR cardiovascularis[All Fields] OR cardiovascularl[All Fields] OR cardiovascularload[All Fields] OR cardiovascularly[All Fields] OR cardiovascularm[All Fields] OR cardiovascularmedicine[All Fields] OR cardiovascularortality[All Fields] OR cardiovascularpathology[All Fields] OR cardiovascularpatients[All Fields] OR cardiovascularprogram[All Fields] OR cardiovascularprophylaxis[All Fields] OR cardiovascularr[All Fields] OR cardiovascularrelated[All Fields] OR cardiovascularrenal[All Fields] OR cardiovascularresearch[All Fields] OR cardiovascural[All Fields] OR cardiovascurlar[All Fields] OR cardiovascutaires[All Fields] OR cardiovascuular[All Fields]) OR (cardioprotectice[All Fields] OR cardioprotectie[All Fields] OR cardioprotectieve[All Fields] OR cardioprotecting[All Fields] OR cardioprotectio[All Fields] OR cardioprotection[All Fields] OR cardioprotection'[All Fields] OR cardioprotections[All Fields] OR cardioprotectiv[All Fields] OR cardioprotective[All Fields] OR cardioprotective'[All Fields] OR cardioprotectiveeffect[All Fields] OR cardioprotectively[All Fields] OR cardioprotectiveness[All Fields] OR cardioprotectives[All Fields] OR cardioprotectivity[All Fields]) OR ("myocardium"[MeSH Terms] OR "myocardium"[All Fields] OR "myocardial"[All Fields])) AND (hasabstract[text] AND ("2004/01/01"[PDAT] : "2014/07/17"[PDAT]) AND "humans"[MeSH Terms]) AND (hasabstract[text] AND ("2004/01/01"[PDAT] : "2014/07/17"[PDAT]) AND "humans"[MeSH Terms] AND English[lang]) AND (hasabstract[text] AND ("2004/01/01"[PDAT] : "2014/07/17"[PDAT]) AND "humans"[MeSH Terms] AND English[lang])
