# Supplementary material for: Cardiovascular and Renal Outcomes of Renin–Angiotensin System Blockade in Adult Patients with Diabetes Mellitus: A Systematic Review with Network Meta-Analyses
Source: PLoS Med. 2016 Mar 8;13(3):e1001971. doi: 10.1371/journal.pmed.1001971 (PMC4783064; doi:10.1371/journal.pmed.1001971)
Supplement: S2 Text — (DOCX) [file pmed.1001971.s019.docx]

**S2 Text. List of screened systematic reviews and meta-analyses**

1. Cheng J, Zhang W, Zhang X, Han F, Li X, He X, Li Q, Chen J. Effect of angiotensin-converting enzyme inhibitors and angiotensin II receptor blockers on all-cause mortality, cardiovascular deaths, and cardiovascular events in patients with diabetes mellitus: a meta-analysis. JAMA Intern Med. 2014;174(5):773-85. PubMed PMID: 24687000.
2. Wu HY, Huang JW, Lin HJ, Liao WC, Peng YS, Hung KY, Wu KD, Tu YK, Chien KL. Comparative effectiveness of renin-angiotensin system blockers and other antihypertensive drugs in patients with diabetes: systematic review and bayesian network meta-analysis. BMJ. 2013;347:f6008. PubMed PMID: 24157497.
3. Blood Pressure Lowering Treatment Trialists' Collaboration, Ninomiya T, Perkovic V, Turnbull F, Neal B, Barzi F, Cass A, Baigent C, Chalmers J, Li N, Woodward M, MacMahon S. Blood pressure lowering and major cardiovascular events in people with and without chronic kidney disease: meta-analysis of randomized controlled trials. BMJ. 2013;347:f5680. PubMed PMID: 24092942.
4. Savarese G, Costanzo P, Cleland JG, Vassallo E, Ruggiero D, Rosano G, Perrone-Filardi P. A meta-analysis reporting effects of angiotensin-converting enzyme inhibitors and angiotensin receptor blockers in patients without heart failure. J Am Coll Cardiol. 2013;61(2):131-42. PubMed PMID: 23219304.
5. Makani H, Bangalore S, Desouza KA, Shah A, Messerli FH. Efficacy and safety of dual blockade of the renin-angiotensin system: meta-analysis of randomized trials. BMJ. 2013;346:f360. PubMed PMID: 23358488.
6. Lv J, Ehteshami P, Sarnak MJ, Tighiouart H, Jun M, Ninomiya T, Foote C, Rodgers A, Zhang H, Wang H, Strippoli GF, Perkovic V. Effects of intensive blood pressure lowering on the progression of chronic kidney disease: a systematic review and meta-analysis. CMAJ. 2013;185(11):949-57. PubMed PMID: 23798459.
7. van Vark LC, Bertrand M, Akkerhuis KM, Brugts JJ, Fox K, Mourad JJ, Boersma E. Angiotensin-converting enzyme inhibitors reduce mortality in hypertension: a meta-analysis of randomized clinical trials of renin-angiotensin-aldosterone system inhibitors involving 158,998 patients. Eur Heart J. 2012;33(16):2088-97. PubMed PMID: 22511654.
8. Vejakama P, Thakkinstian A, Lertrattananon D, Ingsathit A, Ngarmukos C, Attia J. Reno-protective effects of renin-angiotensin system blockade in type 2 diabetic patients: a systematic review and network meta-analysis. Diabetologia. 2012;55(3):566-78. PubMed PMID: 22189484
9. Nakao YM, Teramukai S, Tanaka S, Yasuno S, Fujimoto A, Kasahara M, Ueshima K, Nakao K, Hinotsu S, Nakao K, Kawakami K. Effects of renin-angiotensin system blockades on cardiovascular outcomes in patients with diabetes mellitus: A systematic review and meta-analysis. Diabetes Res Clin Pract. 2012;96(1):68-75.
10. McAlister FA; Renin Angiotension System Modulator Meta-Analysis Investigators. Angiotensin-converting enzyme inhibitors or angiotensin receptor blockers are beneficial in normotensive atherosclerotic patients: a collaborative meta-analysis of randomized trials. Eur Heart J. 2012;33(4):505-14. PubMed PMID: 22041554.
11. Fretheim A, Odgaard-Jensen J, Brørs O, Madsen S, Njølstad I, Norheim OF, Svilaas A, Kristiansen IS, Thürmer H, Flottorp S. Comparative effectiveness of antihypertensive medication for primary prevention of cardiovascular disease: systematic review and multiple treatments meta-analysis. BMC Med. 2012;10:33. PubMed PMID: 22480336
12. Lv J, Perkovic V, Foote CV, Craig ME, Craig JC, Strippoli GF. Antihypertensive agents for preventing diabetic kidney disease. Cochrane Database Syst Rev. 2012;12:CD004136. PubMed PMID: 23235603.
13. Sanders GD, Coeytaux R, Dolor RJ, Hasselblad V, Patel UD, Powers B, Yancy WS, Gray RN, Irvine RJ, Kendrick A. Angiotensin-Converting Enzyme Inhibitors (ACEIs), Angiotensin II Receptor Antagonists (ARBs), and Direct Renin Inhibitors for Treating Essential Hypertension: An Update [Internet]. Rockville (MD): Agency for Healthcare Research and Quality (US); 2011 Jun. Available from <http://www.ncbi.nlm.nih.gov/books/NBK61789/> PubMed PMID: 21977520
14. Maione A, Navaneethan SD, Graziano G, Mitchell R, Johnson D, Mann JF, Gao P, Craig JC, Tognoni G, Perkovic V, Nicolucci A, De Cosmo S, Sasso A, Lamacchia O, Cignarelli M, Manfreda VM, Gentile G, Strippoli GF. Angiotensin-converting enzyme inhibitors, angiotensin receptor blockers and combined therapy in patients with micro- and macroalbuminuria and other cardiovascular risk factors: a systematic review of randomized controlled trials. Nephrol Dial Transplant. 2011;26(9):2827-47. PubMed PMID: 21372254.
15. Bangalore S, Kumar S, Wetterslev J, Messerli FH. Angiotensin receptor blockers and risk of myocardial infarction: meta-analyses and trial sequential analyses of 147 020 patients from randomised trials. BMJ. 2011;342:d2234. PubMed PMID: 21521728;
16. Sciarretta S, Palano F, Tocci G, Baldini R, Volpe M. Antihypertensive treatment and development of heart failure in hypertension: a Bayesian network meta-analysis of studies in patients with hypertension and high cardiovascular risk. Arch Intern Med. 2011;171(5):384-94. PubMed PMID: 21059964.
17. Kuenzli A, Bucher HC, Anand I, Arutiunov G, Kum LC, McKelvie R, Afzal R, White M, Nordmann AJ. Meta-analysis of combined therapy with angiotensin receptor antagonists versus ACE inhibitors alone in patients with heart failure. PLoS One. 2010;5(4):e9946. PubMed PMID: 20376345.
18. Baker WL, Coleman CI, Kluger J, Reinhart KM, Talati R, Quercia R, Phung OJ, White CM. Systematic review: comparative effectiveness of angiotensin-converting enzyme inhibitors or angiotensin II-receptor blockers for ischemic heart disease. Ann Intern Med. 2009;151(12):861-71. PubMed PMID: 20008762.
19. Wright JM, Musini VM. First-line drugs for hypertension. Cochrane Database Syst Rev. 2009 Jul 8;(3):CD001841. PubMed PMID: 19588327.
20. Law MR, Morris JK, Wald NJ. Use of blood pressure lowering drugs in the prevention of cardiovascular disease: meta-analysis of 147 randomised trials in the context of expectations from prospective epidemiological studies. BMJ. 2009;338:b1665. PubMed PMID: 19454737.
21. Volpe M, Tocci G, Sciarretta S, Verdecchia P, Trimarco B, Mancia G. Angiotensin II receptor blockers and myocardial infarction: an updated analysis of randomized clinical trials. J Hypertens. 2009;27(5):941-6. PubMed PMID: 19381108.
22. Sarafidis PA, Stafylas PC, Kanaki AI, Lasaridis AN. Effects of renin-angiotensin system blockers on renal outcomes and all-cause mortality in patients with diabetic nephropathy: an updated meta-analysis. Am J Hypertens. 2008;21(8):922-9. PubMed PMID: 18535536.
23. Reboldi G, Angeli F, Cavallini C, Gentile G, Mancia G, Verdecchia P. Comparison between angiotensin-converting enzyme inhibitors and angiotensin receptor blockers on the risk of myocardial infarction, stroke and death: a meta-analysis. J Hypertens. 2008;26(7):1282-9. PubMed PMID: 18550998.
24. Kunz R, Friedrich C, Wolbers M, Mann JF. Meta-analysis: effect of monotherapy and combination therapy with inhibitors of the renin angiotensin system on proteinuria in renal disease. Ann Intern Med. 2008;148(1):30-48. PubMed PMID: 17984482.
25. Bangalore S, Kumar S, Lobach I, Messerli FH. Blood pressure targets in subjects with type 2 diabetes mellitus/impaired fasting glucose: observations from traditional and bayesian random-effects meta-analyses of randomized trials. Circulation. 2011;123(24):2799-810, 9 p following 810. PubMed PMID: 21632497
26. Strippoli GF, Bonifati C, Craig M, Navaneethan SD, Craig JC. Angiotensin converting enzyme inhibitors and angiotensin II receptor antagonists for preventing the progression of diabetic kidney disease. Cochrane Database Syst Rev. 2006;(4):CD006257. PubMed PMID: 17054288.
27. Casas JP, Chua W, Loukogeorgakis S, Vallance P, Smeeth L, Hingorani AD, MacAllister RJ. Effect of inhibitors of the renin-angiotensin system and other antihypertensive drugs on renal outcomes: systematic review and meta-analysis. Lancet. 2005;366(9502):2026-33. PubMed PMID: 16338452.
28. McDonald MA, Simpson SH, Ezekowitz JA, Gyenes G, Tsuyuki RT. Angiotensin receptor blockers and risk of myocardial infarction: systematic review. BMJ. 2005;331(7521):873. PubMed PMID: 16183653.
29. Strippoli GF, Craig M, Schena FP, Craig JC. Antihypertensive agents for primary prevention of diabetic nephropathy. J Am Soc Nephrol. 2005;16(10):3081-91. PubMed PMID: 16135776.
30. Turnbull F, Neal B, Algert C, Chalmers J, Chapman N, Cutler J, Woodward M, MacMahon S; Blood Pressure Lowering Treatment Trialists' Collaboration. Effects of different blood pressure-lowering regimens on major cardiovascular events in individuals with and without diabetes mellitus: results of prospectively designed overviews of randomized trials. Arch Intern Med. 2005;165(12):1410-9. PubMed PMID: 15983291.
31. Strippoli GF, Craig M, Deeks JJ, Schena FP, Craig JC. Effects of angiotensin converting enzyme inhibitors and angiotensin II receptor antagonists on mortality and renal outcomes in diabetic nephropathy: systematic review. BMJ. 2004;329(7470):828. PubMed PMID: 15459003.
32. Psaty BM, Lumley T, Furberg CD, Schellenbaum G, Pahor M, Alderman MH, Weiss NS. Health outcomes associated with various antihypertensive therapies used as first-line agents: a network meta-analysis. JAMA. 2003;289(19):2534-44. PubMed PMID: 12759325.
