# Supplementary material for: Cardiovascular and Renal Outcomes of Renin–Angiotensin System Blockade in Adult Patients with Diabetes Mellitus: A Systematic Review with Network Meta-Analyses
Source: PLoS Med. 2016 Mar 8;13(3):e1001971. doi: 10.1371/journal.pmed.1001971 (PMC4783064; doi:10.1371/journal.pmed.1001971)
Supplement: S3 Text — (DOCX) [file pmed.1001971.s020.docx]

**S3 Text. Example of WinBUGS code for main analyses**

model{

for(i in 1:ns){ # Loop through studies

# Adjustment for multi-arm trials is zero for control arm

w[i,1] <- 0

# Treatment effect is zero for control arm

delta[i,1] <- 0

# Vague priors for trial baselines

mu[i] ~ dnorm(0,.0001)

# Binomial likelihood

# Loop through arms

for (k in 1:na[i]) {

r[i,k] ~ dbin(p[i,k],n[i,k])

#parametization of the model for NMA

logit(p[i,k]) <- mu[i] + delta[i,k] # Model for linear predictor

rhat[i,k] <- p[i,k] * n[i,k] # Expected value of the numerators

dev[i,k] <- 2 * (r[i,k] * (log(r[i,k])-log(rhat[i,k])) # Deviance contribution

+ (n[i,k]-r[i,k]) * (log(n[i,k]-r[i,k])-log(n[i,k]-rhat[i,k])))

}

# Summed residual deviance

resdev[i] <- sum(dev[i, 1:na[i]])

# contribution for this trial

# Loop through arms

for (k in 2:na[i]) {

delta[i,k] ~ dnorm(md[i,k],taud[i,k]) # Trial-specific LOR distributions

md[i,k] <- d[t[i,k]] - d[t[i,1]] + sw[i,k] # Mean of LOR distributions

# (with multi-arm trial correction)

taud[i,k] <- tau *2*(k-1)/k # Precision of LOR distributions

# (with multi-arm trial correction)

w[i,k] <- (delta[i,k] - d[t[i,k]] + d[t[i,1]]) # Adjustment for multi-arm RCTs

sw[i,k] <- sum(w[i,1:k-1])/(k-1) # Cumulative adjustment for multi-arm trials

}

}

# Total Residual Deviance

totresdev <- sum(resdev[])

# treatment effect is zero for reference treatment

d[1]<-0

#priors#

for (k in 2:nt){ d[k] ~ dnorm(0,.0001) } # vague priors for treatment effects

sd ~ dunif(0,5) # vague prior for between-trial SD

tau <- pow(sd,-2) # between-trial precision = (1/between-trial variance)

# Pairwise ORs and LORs for all possible pair-wise comparisons, if nt>2

for (c in 1:(nt-1)) {

for (k in (c+1):nt) {

lor[c,k] <- (d[k]-d[c])

or[c,k] <- exp(lor[c,k])

}

}

# Surface Under the Cumulative Ranking curve (SUCRA)

for(k in 1:nt) {

sucra[k] <- sum(cumeffectiveness[k,1:(nt-1)])/ (nt-1)

}

# Predictive distribution for future trial is multivariate normal

delta.new[1] <- 0 # Treatment effect is zero for reference treatment

w.new[1] <- 0 # Adjustment for conditional mean is zero for reference treatment

# Loop through treatments

for (k in 2:nt) {

delta.new[k] ~ dnorm(m.new[k],tau.new[k]) # Conditional distribution of each delta.new

m.new[k] <- d[k] + sw.new[k] # Conditional mean of delta.new

tau.new[k] <- tau *2*(k-1)/k # Conditional precision of delta.new

w.new[k] <- delta.new[k] - d[k] # Adjustment for conditional mean

sw.new[k] <- sum(w.new[1:k-1])/(k-1) # Cumulative adjustment for conditional mean

}

p.base ~ dbeta(a,b) # Draw baseline (control group) effect

a <- r[ns,1] # No. of events in control group

b <- n[ns,1] - r[ns,1] # No. of non-events in control group

# Loop through treatments or studies?

for (k in 2:na[ns]) {

# Predictive probability of event for each treatment arm of the new trial

logit(p.new[k]) <- logit(p.base) + (delta.new[t[ns,k]]- delta.new[t[ns,1]])

r.new[k] ~ dbin(p.new[k], n[ns,k]) # draw predicted number of events for each arm of the new trial

# Bayesian p-value: probability of obtaining a value as extreme as the

# value observed (r[ns,2]), given the model and the remaining data

p.cross[k] <- step(r[ns,2] - r.new[k]) - 0.5*equals(r.new[k],r[ns,2]) # extreme value “smaller”

}

# pairwise ORs and LORs for all possible pair-wise comparisons, if nt>2

for (c in 1:(nt-1)) {

for (k in (c+1):nt) {

lor.new[c,k] <- delta.new[k]- delta.new[c]

or.new[c,k] <- exp(lor.new[c,k])

}

}

} # End of model
