# Supplementary material for: Cardiovascular and Renal Outcomes of Renin–Angiotensin System Blockade in Adult Patients with Diabetes Mellitus: A Systematic Review with Network Meta-Analyses
Source: PLoS Med. 2016 Mar 8;13(3):e1001971. doi: 10.1371/journal.pmed.1001971 (PMC4783064; doi:10.1371/journal.pmed.1001971)
Supplement: S4 Text — (DOCX) [file pmed.1001971.s021.docx]

**S4 Text. List of included clinical trials (71 studies described in 88 publications)**

1. Parving HH, Hommel E, Damkjaer Nielsen M, Giese J. Effect of captopril on blood pressure and kidney function in normotensive insulin dependent diabetics with nephropathy. BMJ. 1989;299(6698):533-6. PubMed PMID: 2507061.
2. Parving HH, Hommel E, Jensen BR, Hansen HP. Long-term beneficial effect of ACE inhibition on diabetic nephropathy in normotensive type 1 diabetic patients. Kidney Int. 2001;60(1):228-34. PubMed PMID: 11422755.
3. Bauer JH, Reams GP, Hewett J, Klachko D, Lau A, Messina C, Knaus V. A randomized, double-blind, placebo-controlled trial to evaluate the effect of enalapril in patients with clinical diabetic nephropathy. Am J Kidney Dis. 1992;20:443-57. PubMed PMID: 1442757.
4. Björck S, Mulec H, Johnsen SA, Nordén G, Aurell M. Renal protective effect of enalapril in diabetic nephropathy. BMJ. 1992 Feb 8;304(6823):339-43. PubMed PMID: 1540729.
5. Chan JC, Cockram CS, Nicholls MG, Cheung CK, Swaminathan R. Comparison of enalapril and nifedipine in treating non-insulin dependent diabetes associated with hypertension: one year analysis. BMJ. 1992 Oct 24;305(6860):981-5. PubMed PMID: 1458144.
6. Lacourcière Y, Nadeau A, Poirier L, Tancrède G. Captopril or conventional therapy in hypertensive type II diabetics. Three-year analysis. Hypertension. 1993;21(6 Pt 1):786-94. PubMed PMID: 8500859.
7. Lewis EJ, Hunsicker LG, Bain RP, Rohde RD. The effect of angiotensin-converting-enzyme inhibition on diabetic nephropathy. The Collaborative Study Group. N Engl J Med. 1993;329(20):1456-62. Erratum in: N Engl J Med 1993;330(2):152. PubMed PMID: 8413456.
8. Ravid M, Savin H, Jutrin I, Bental T, Katz B, Lishner M. Long-term stabilizing effect of angiotensin-converting enzyme inhibition on plasma creatinine and on proteinuria in normotensive type II diabetic patients. Ann Intern Med. 1993;118(8):577-81. PubMed PMID: 8452322.
9. Elving LD, Wetzels JF, van Lier HJ, de Nobel E, Berden JH. Captopril and atenolol are equally effective in retarding progression of diabetic nephropathy. Results of a 2-year prospective, randomized study. Diabetologia. 1994;37(6):604-9. PubMed PMID: 7926346.
10. Sano T, Kawamura T, Matsumae H, Sasaki H, Nakayama M, Hara T, Matsuo S, Hotta N, Sakamoto N. Effects of long-term enalapril treatment on persistent micro-albuminuria in well-controlled hypertensive and normotensive NIDDM patients. Diabetes Care. 1994;17(5):420-4. PubMed PMID: 8062609.
11. Laffel LM, McGill JB, Gans DJ. The beneficial effect of angiotensin-converting enzyme inhibition with captopril on diabetic nephropathy in normotensive IDDM patients with microalbuminuria. North American Microalbuminuria Study Group. Am J Med. 1995 Nov;99(5):497-504. PubMed PMID: 7485207.
12. Bakris GL, Copley JB, Vicknair N, Sadler R, Leurgans S. Calcium channel blockers versus other antihypertensive therapies on progression of NIDDM associated nephropathy. Kidney Int. 1996 Nov;50(5):1641-50. PubMed PMID: 8914031.
13. Captopril reduces the risk of nephropathy in IDDM patients with microalbuminuria. The Microalbuminuria Captopril Study Group. Diabetologia. 1996;39(5):587-93. PubMed PMID: 8739919.
14. Nielsen FS, Rossing P, Gall MA, Skøtt P, Smidt UM, Parving HH. Long-term effect of lisinopril and atenolol on kidney function in hypertensive NIDDM subjects with diabetic nephropathy. Diabetes. 1997 Jul;46(7):1182-8. PubMed PMID: 9200654.
15. Nielsen FS, Rossing P, Gall MA, Skøtt P, Smidt UM, Parving HH. Impact of lisinopril and atenolol on kidney function in hypertensive NIDDM subjects with diabetic nephropathy. Diabetes. 1994 Sep;43(9):1108-13. PubMed PMID: 8070610.
16. Estacio RO, Jeffers BW, Hiatt WR, Biggerstaff SL, Gifford N, Schrier RW. The effect of nisoldipine as compared with enalapril on cardiovascular outcomes in patients with non-insulin-dependent diabetes and hypertension. N Engl J Med. 1998;338(10):645-52. PubMed PMID: 9486993.
17. Schrier RW, Estacio RO. Additional follow-up from the ABCD trial in patients with type 2 diabetes and hypertension. N Engl J Med. 2000;343(26):1969. PubMed PMID: 11186674.
18. Schrier RW, Estacio RO, Esler A, Mehler P. Effects of aggressive blood pressure control in normotensive type 2 diabetic patients on albuminuria, retinopathy and strokes. Kidney Int. 2002;61(3):1086-97. PubMed PMID:11849464.
19. Crepaldi G, Carta Q, Deferrari G, Mangili R, Navalesi R, Santeusanio F, Spalluto A, Vanasia A, Villa GM, Nosadini R. Effects of lisinopril and nifedipine on the progression to overt albuminuria in IDDM patients with incipient nephropathy and normal blood pressure. The Italian Microalbuminuria Study Group in IDDM. Diabetes Care. 1998;21(1):104-10. PubMed PMID: 9538979.
20. Tatti P, Pahor M, Byington RP, Di Mauro P, Guarisco R, Strollo G, Strollo F. Outcome results of the Fosinopril Versus Amlodipine Cardiovascular Events Randomized Trial (FACET) in patients with hypertension and NIDDM. Diabetes Care. 1998;21(4):597-603. PubMed PMID: 9571349.
21. Nankervis A, Nicholls K, Kilmartin G, Allen P, Ratnaike S, Martin FI. Effects of perindopril on renal histomorphometry in diabetic subjects with microalbuminuria: a 3-year placebo-controlled biopsy study. Metabolism. 1998;47(12 Suppl 1):12-5. PubMed PMID: 9867064.
22. Ravid M, Brosh D, Levi Z, Bar-Dayan Y, Ravid D, Rachmani R. Use of enalapril to attenuate decline in renal function in normotensive, normoalbuminuric patients with type 2 diabetes mellitus. A randomized, controlled trial. Ann Intern Med. 1998;128(12 Pt 1):982-8. PubMed PMID: 9625684.
23. Efficacy of atenolol and captopril in reducing risk of macrovascular and microvascular complications in type 2 diabetes: UKPDS 39. UK Prospective Diabetes Study Group. BMJ. 1998;317(7160):713-20. PubMed PMID: 9732338.
24. Fogari R, Zoppi A, Corradi L, Mugellini A, Lazzari P, Preti P, Lusardi P. Long-term effects of ramipril and nitrendipine on albuminuria in hypertensive patients with type II diabetes and impaired renal function. J Hum Hypertens. 1999;13(1):47-53. PubMed PMID: 9928752.
25. O'Hare P, Bilbous R, Mitchell T, O' Callaghan CJ, Viberti GC; Ace-Inhibitor Trial to Lower Albuminuria in Normotensive Insulin-Dependent Subjects Study Group. Low-dose ramipril reduces microalbuminuria in type 1 diabetic patients without hypertension: results of a randomized controlled trial. Diabetes Care. 2000;23(12):1823-9. PubMed PMID: 11128360.
26. Tarnow L, Rossing P, Jensen C, Hansen BV, Parving HH. Long-term renoprotective effect of nisoldipine and lisinopril in type 1 diabetic patients with diabetic nephropathy. Diabetes Care. 2000;23(12):1725-30. PubMed PMID: 11128341.
27. Chan JC, Ko GT, Leung DH, Cheung RC, Cheung MY, So WY, Swaminathan R, Nicholls MG, Critchley JA, Cockram CS. Long-term effects of angiotensin-converting enzyme inhibition and metabolic control in hypertensive type 2 diabetic patients. Kidney Int. 2000;57(2):590-600. PubMed PMID: 10652036.
28. Lindholm LH, Hansson L, Ekbom T, Dahlöf B, Lanke J, Linjer E, Scherstén B, Wester PO, Hedner T, de Faire U. Comparison of antihypertensive treatments in preventing cardiovascular events in elderly diabetic patients: results from the Swedish Trial in Old Patients with Hypertension-2. STOP Hypertension-2 Study Group. J Hypertens. 2000;18(11):1671-5. PubMed PMID: 11081782.
29. Effects of ramipril on cardiovascular and microvascular outcomes in people with diabetes mellitus: results of the HOPE study and MICRO-HOPE substudy. Heart Outcomes Prevention Evaluation Study Investigators. Lancet. 2000;355(9200):253-9. Erratum in: Lancet 2000;356(9232):860. PubMed PMID: 10675071.
30. Baba S; J-MIND Study Group. Nifedipine and enalapril equally reduce the progression of nephropathy in hypertensive type 2 diabetics. Diabetes Res Clin Pract. 2001;54(3):191-201. PubMed PMID: 11689274.
31. Lewis EJ, Hunsicker LG, Clarke WR, Berl T, Pohl MA, Lewis JB, Ritz E, Atkins RC, Rohde R, Raz I; Collaborative Study Group. Renoprotective effect of the angiotensin-receptor antagonist irbesartan in patients with nephropathy due to type 2 diabetes. N Engl J Med. 2001;345(12):851-60. PubMed PMID: 11565517.
32. Berl T, Hunsicker LG, Lewis JB, Pfeffer MA, Porush JG, Rouleau JL, Drury PL, Esmatjes E, Hricik D, Parikh CR, Raz I, Vanhille P, Wiegmann TB, Wolfe BM, Locatelli F, Goldhaber SZ, Lewis EJ; Irbesartan Diabetic Nephropathy Trial. Collaborative Study Group. Cardiovascular outcomes in the Irbesartan Diabetic Nephropathy Trial of patients with type 2 diabetes and overt nephropathy. Ann Intern Med. 2003;138(7):542-9. PubMed PMID: 12667024.
33. Parving HH, Lehnert H, Bröchner-Mortensen J, Gomis R, Andersen S, Arner P; Irbesartan in Patients with Type 2 Diabetes and Microalbuminuria Study Group. The effect of irbesartan on the development of diabetic nephropathy in patients with type 2 diabetes. N Engl J Med. 2001 Sep 20;345(12):870-8. PubMed PMID: 11565519.
34. Jerums G, Allen TJ, Campbell DJ, Cooper ME, Gilbert RE, Hammond JJ, Raffaele J, Tsalamandris C. Long-term comparison between perindopril and nifedipine in normotensive patients with type 1 diabetes and microalbuminuria. Am J Kidney Dis. 2001;37(5):890-9. PubMed PMID: 11325669.
35. Brenner BM, Cooper ME, de Zeeuw D, Keane WF, Mitch WE, Parving HH, Remuzzi G, Snapinn SM, Zhang Z, Shahinfar S; RENAAL Study Investigators. Effects of losartan on renal and cardiovascular outcomes in patients with type 2 diabetes and nephropathy. N Engl J Med. 2001;345(12):861-9. PubMed PMID: 11565518.
36. Merck Research Laboratories. FDA Advisory Committee Background Information: NDA 20-386/S-028 COZAAR (losartan potasium). Cardiovascular and Renal Drugs Advisory Committee, 12 April 2002. Available at: <http://www.fda.gov/ohrms/dockets/ac/02/briefing/3849b1_01_Merck.pdf> [consulted October 9, 2014]
37. Niskanen L, Hedner T, Hansson L, Lanke J, Niklason A; CAPPP Study Group. Reduced cardiovascular morbidity and mortality in hypertensive diabetic patients on first-line therapy with an ACE inhibitor compared with a diuretic/beta-blocker-based treatment regimen: a subanalysis of the Captopril Prevention Project. Diabetes Care. 2001;24(12):2091-6. PubMed PMID: 11723089.
38. Cohn JN, Tognoni G; Valsartan Heart Failure Trial Investigators. A randomized trial of the angiotensin-receptor blocker valsartan in chronic heart failure. N Engl J Med. 2001;345(23):1667-75. PubMed PMID: 11759645.
39. Fogari R, Preti P, Zoppi A, Rinaldi A, Corradi L, Pasotti C, Poletti L, Marasi G, Derosa G, Mugellini A, Voglini C, Lazzari P. Effects of amlodipine fosinopril combination on microalbuminuria in hypertensive type 2 diabetic patients. Am J Hypertens. 2002;15(12):1042-9. PubMed PMID: 12460699.
40. Katayama S, Kikkawa R, Isogai S, Sasaki N, Matsuura N, Tajima N, Urakami T, Uchigata Y, Ohashi Y. Effect of captopril or imidapril on the progression of diabetic nephropathy in Japanese with type 1 diabetes mellitus: a randomized controlled study (JAPAN-IDDM). Diabetes Res Clin Pract. 2002;55(2):113-21. Erratum in: Diabetes Res Clin Pract 2002 Jul;57(1):71. PubMed PMID: 11796177.
41. Lindholm LH, Ibsen H, Dahlöf B, Devereux RB, Beevers G, de Faire U, Fyhrquist F, Julius S, Kjeldsen SE, Kristiansson K, Lederballe-Pedersen O, Nieminen MS, Omvik P, Oparil S, Wedel H, Aurup P, Edelman J, Snapinn S; LIFE Study Group. Cardiovascular morbidity and mortality in patients with diabetes in the Losartan Intervention For Endpoint reduction in hypertension study (LIFE): a randomised trial against atenolol. Lancet. 2002;359(9311):1004-10. PubMed PMID: 11937179.
42. Pfeffer MA, McMurray JJ, Velazquez EJ, Rouleau JL, Køber L, Maggioni AP, Solomon SD, Swedberg K, Van de Werf F, White H, Leimberger JD, Henis M, Edwards S, Zelenkofske S, Sellers MA, Califf RM; Valsartan in Acute Myocardial Infarction Trial Investigators. Valsartan, captopril, or both in myocardial infarction complicated by heart failure, left ventricular dysfunction, or both. N Engl J Med. 2003;349(20):1893-906. Erratum in: N Engl J Med. 2004;350(2):203. PubMed PMID: 14610160.
43. Julius S, Kjeldsen SE, Weber M, Brunner HR, Ekman S, Hansson L, Hua T, Laragh J, McInnes GT, Mitchell L, Plat F, Schork A, Smith B, Zanchetti A; VALUE trial group. Outcomes in hypertensive patients at high cardiovascular risk treated with regimens based on valsartan or amlodipine: the VALUE randomised trial. Lancet. 2004;363(9426):2022-31. PubMed PMID: 15207952.
44. Ruggenenti P, Fassi A, Ilieva AP, Bruno S, Iliev IP, Brusegan V, Rubis N, Gherardi G, Arnoldi F, Ganeva M, Ene-Iordache B, Gaspari F, Perna A, Bossi A, Trevisan R, Dodesini AR, Remuzzi G; Bergamo Nephrologic Diabetes Complications Trial (BENEDICT) Investigators. Preventing microalbuminuria in type 2 diabetes. N Engl J Med. 2004;351(19):1941-51. PubMed PMID: 15516697.
45. Barnett AH, Bain SC, Bouter P, Karlberg B, Madsbad S, Jervell J, Mustonen J; Diabetics Exposed to Telmisartan and Enalapril Study Group. Angiotensin-receptor blockade versus converting-enzyme inhibition in type 2 diabetes and nephropathy. N Engl J Med. 2004;351(19):1952-61. Erratum in: N Engl J Med. 2005 Apr 21;352(16)1731. PubMed PMID: 15516696.
46. Barnett A. Preventing renal complications in type 2 diabetes: results of the diabetics exposed to telmisartan and enalapril trial. J Am Soc Nephrol. 2006;17(4 Suppl 2):S132-5. PubMed PMID: 16565237.
47. Marre M, Lievre M, Chatellier G, Mann JF, Passa P, Ménard J; DIABHYCAR Study Investigators. Effects of low dose ramipril on cardiovascular and renal outcomes in patients with type 2 diabetes and raised excretion of urinary albumin: randomised, double blind, placebo controlled trial (the DIABHYCAR study). BMJ. 2004;328(7438):495. Epub 2004 Feb 11. Erratum in: BMJ. 2004;328(7441):686. PubMed PMID: 14960504.
48. Marre M, Puig JG, Kokot F, Fernandez M, Jermendy G, Opie L, Moyseev V, Scheen A, Ionescu-Tirgoviste C, Saldanha MH, Halabe A, Williams B, Mion Júnior D, Ruiz M, Hermansen K, Tuomilehto J, Finizola B, Gallois Y, Amouyel P, Ollivier JP, Asmar R. Equivalence of indapamide SR and enalapril on microalbuminuria reduction in hypertensive patients with type 2 diabetes: the NESTOR Study. J Hypertens. 2004;22(8):1613-22. PubMed PMID: 15257186.
49. Yui Y, Sumiyoshi T, Kodama K, Hirayama A, Nonogi H, Kanmatsuse K, Origasa H, Iimura O, Ishii M, Saruta T, Arakawa K, Hosoda S, Kawai C. Nifedipine retard was as effective as angiotensin converting enzyme inhibitors in preventing cardiac events in high-risk hypertensive patients with diabetes and coronary artery disease: the Japan Multicenter Investigation for Cardiovascular Diseases-B (JMIC-B) subgroup analysis. Hypertens Res. 2004;27(7):449-56. Erratum in: Hypertens Res. 2004;27(9):695. PubMed PMID: 15302980.
50. Ko GT, Tsang CC, Chan HC. Stabilization and regression of albuminuria in Chinese patients with type 2 diabetes: a one-year randomized study of valsartan versus enalapril. Adv Ther. 2005 Mar-Apr;22(2):155-62. PubMed PMID: 16020405.
51. Schram MT, van Ittersum FJ, Spoelstra-de Man A, van Dijk RA, Schalkwijk CG, Ijzerman RG, Twisk JW, Stehouwer CD. Aggressive antihypertensive therapy based on hydrochlorothiazide, candesartan or lisinopril as initial choice in hypertensive type II diabetic individuals: effects on albumin excretion, endothelial function and inflammation in a double-blind, randomized clinical trial. J Hum Hypertens. 2005;19(6):429-37. PubMed PMID: 15647778.
52. Daly CA, Fox KM, Remme WJ, Bertrand ME, Ferrari R, Simoons ML; EUROPA Investigators. The effect of perindopril on cardiovascular morbidity and mortality in patients with diabetes in the EUROPA study: results from the PERSUADE substudy. Eur Heart J. 2005;26(14):1369-78. PMID: 15860521.
53. Whelton PK, Barzilay J, Cushman WC, Davis BR, Iiamathi E, Kostis JB, Leenen FH, Louis GT, Margolis KL, Mathis DE, Moloo J, Nwachuku C, Panebianco D, Parish DC, Pressel S, Simmons DL, Thadani U; ALLHAT Collaborative Research Group. Clinical outcomes in antihypertensive treatment of type 2 diabetes, impaired fasting glucose concentration, and normoglycemia: Antihypertensive and Lipid-Lowering Treatment to Prevent Heart Attack Trial (ALLHAT). Arch Intern Med. 2005;165(12):1401-9. PubMed PMID: 15983290.
54. Rahman M, Pressel S, Davis BR, Nwachuku C, Wright JT Jr, Whelton PK, Barzilay J, Batuman V, Eckfeldt JH, Farber M, Henriquez M, Kopyt N, Louis GT, Saklayen M, Stanford C, Walworth C, Ward H, Wiegmann T. Renal outcomes in high-risk hypertensive patients treated with an angiotensin-converting enzyme inhibitor or a calcium channel blocker vs a diuretic: a report from the Antihypertensive and Lipid-Lowering Treatment to Prevent Heart Attack Trial (ALLHAT). Arch Intern Med. 2005;165(8):936-46. PubMed PMID: 15851647.
55. Trenkwalder P, Elmfeldt D, Hofman A, Lithell H, Olofsson B, Papademetriou V, Skoog I, Zanchetti A; Study on COgnition and Prognosis in the Elderly (SCOPE). The Study on COgnition and Prognosis in the Elderly (SCOPE) - major CV events and stroke in subgroups of patients. Blood Press. 2005;14(1):31-7. PubMed PMID: 15823945.
56. Estacio RO, Coll JR, Tran ZV, Schrier RW. Effect of intensive blood pressure control with valsartan on urinary albumin excretion in normotensive patients with type 2 diabetes. Am J Hypertens. 2006;19(12):1241-8. PubMed PMID: 17161769.
57. Tong PC, Ko GT, Chan WB, Ma RC, So WY, Lo MK, Lee KF, Ozaki R, Chow CC, Cockram CS, Chan JC. The efficacy and tolerability of fosinopril in Chinese type 2 diabetic patients with moderate renal insufficiency. Diabetes Obes Metab. 2006;8(3):342-7. PubMed PMID: 16634995.
58. Patel A; ADVANCE Collaborative Group, MacMahon S, Chalmers J, Neal B, Woodward M, Billot L, Harrap S, Poulter N, Marre M, Cooper M, Glasziou P, Grobbee DE, Hamet P, Heller S, Liu LS, Mancia G, Mogensen CE, Pan CY, Rodgers A, Williams B. Effects of a fixed combination of perindopril and indapamide on macrovascular and microvascular outcomes in patients with type 2 diabetes mellitus (the ADVANCE trial): a randomised controlled trial. Lancet. 2007;370(9590):829-40. PubMed PMID: 17765963.
59. de Galan BE, Perkovic V, Ninomiya T, Pillai A, Patel A, Cass A, Neal B, Poulter N, Harrap S, Mogensen CE, Cooper M, Marre M, Williams B, Hamet P, Mancia G, Woodward M, Glasziou P, Grobbee DE, MacMahon S, Chalmers J; ADVANCE Collaborative Group. Lowering blood pressure reduces renal events in type 2 diabetes. J Am Soc Nephrol. 2009;20(4):883-92. PubMed PMID: 19225038.
60. Chaturvedi N, Porta M, Klein R, Orchard T, Fuller J, Parving HH, Bilous R, Sjølie AK; DIRECT Programme Study Group. Effect of candesartan on prevention (DIRECT-Prevent 1) and progression (DIRECT-Protect 1) of retinopathy in type 1 diabetes: randomised, placebo-controlled trials. Lancet. 2008;372(9647):1394-402. PubMed PMID: 18823656.
61. Bilous R, Chaturvedi N, Sjølie AK, Fuller J, Klein R, Orchard T, Porta M, Parving HH. Effect of candesartan on microalbuminuria and albumin excretion rate in diabetes: three randomized trials. Ann Intern Med. 2009;151(1):11-20, W3-4. PubMed PMID: 19451554.
62. Sjølie AK, Klein R, Porta M, Orchard T, Fuller J, Parving HH, Bilous R, Chaturvedi N; DIRECT Programme Study Group. Effect of candesartan on progression and regression of retinopathy in type 2 diabetes (DIRECT-Protect 2): a randomized placebo-controlled trial. Lancet. 2008;372(9647):1385-93. PubMed PMID: 18823658.
63. Tillin T, Orchard T, Malm A, Fuller J, Chaturvedi N. The role of antihypertensive therapy in reducing vascular complications of type 2 diabetes. Findings from the DIabetic REtinopathy Candesartan Trials-Protect 2 study. J Hypertens. 2011;29(7):1457-62. PubMed PMID: 21602709.
64. Bakris GL, Toto RD, McCullough PA, Rocha R, Purkayastha D, Davis P; GUARD (Gauging Albuminuria Reduction With Lotrel in Diabetic Patients With Hypertension) Study Investigators. Effects of different ACE inhibitor combinations on albuminuria: results of the GUARD study. Kidney Int. 2008;73(11):1303-9. PubMed PMID: 18354383.
65. Yusuf S, Diener HC, Sacco RL, Cotton D, Ounpuu S, Lawton WA, Palesch Y, Martin RH, Albers GW, Bath P, Bornstein N, Chan BP, Chen ST, Cunha L, Dahlöf B, De Keyser J, Donnan GA, Estol C, Gorelick P, Gu V, Hermansson K, Hilbrich L, Kaste M, Lu C, Machnig T, Pais P, Roberts R, Skvortsova V, Teal P, Toni D, VanderMaelen C, Voigt T, Weber M, Yoon BW; PRoFESS Study Group. Telmisartan to prevent recurrent stroke and cardiovascular events. N Engl J Med. 2008;359(12):1225-37. PubMed PMID: 18753639.
66. Mann JF, Anderson C, Gao P, Gerstein HC, Boehm M, Rydén L, Sleight P, Teo KK, Yusuf S; ONTARGET investigators. Dual inhibition of the renin-angiotensin system in high-risk diabetes and risk for stroke and other outcomes: results of the ONTARGET trial. J Hypertens. 2013;31(2):414-21. PubMed PMID: 23249829.
67. Mann JF, Schmieder RE, McQueen M, Dyal L, Schumacher H, Pogue J, Wang X, Maggioni A, Budaj A, Chaithiraphan S, Dickstein K, Keltai M, Metsärinne K, Oto A, Parkhomenko A, Piegas LS, Svendsen TL, Teo KK, Yusuf S; ONTARGET investigators. Renal outcomes with telmisartan, ramipril, or both, in people at high vascular risk (the ONTARGET study): a multicentre, randomised, double-blind, controlled trial. Lancet. 2008;372(9638):547-53. PubMed PMID: 18707986.
68. ONTARGET Investigators, Yusuf S, Teo KK, Pogue J, Dyal L, Copland I, Schumacher H, Dagenais G, Sleight P, Anderson C. Telmisartan, ramipril, or both in patients at high risk for vascular events. N Engl J Med. 2008;358(15):1547-59. PubMed PMID: 18378520.
69. Telmisartan Randomised AssessmeNt Study in ACE iNtolerant subjects with cardiovascular Disease (TRANSCEND) Investigators, Yusuf S, Teo K, Anderson C, Pogue J, Dyal L, Copland I, Schumacher H, Dagenais G, Sleight P. Effects of the angiotensin-receptor blocker telmisartan on cardiovascular events in high-risk patients intolerant to angiotensin-converting enzyme inhibitors: a randomised controlled trial. Lancet. 2008;372(9644):1174-83. Erratum in: Lancet. 2008;372(9647):1384. PubMed PMID: 18757085.
70. Mann JF, Schmieder RE, Dyal L, McQueen MJ, Schumacher H, Pogue J, Wang X, Probstfield JL, Avezum A, Cardona-Munoz E, Dagenais GR, Diaz R, Fodor G, Maillon JM, Rydén L, Yu CM, Teo KK, Yusuf S; TRANSCEND (Telmisartan Randomised Assessment Study in ACE Intolerant Subjects with Cardiovascular Disease) Investigators. Effect of telmisartan on renal outcomes: a randomized trial. Ann Intern Med. 2009;151(1):1-10, W1-2. PubMed PMID: 19451556.
71. Foulquier S, Böhm M, Schmieder R, Sleight P, Teo K, Yusuf S, Schumacher H, Unger T. Impact of telmisartan on cardiovascular outcome in hypertensive patients at high risk: a Telmisartan Randomised AssessmeNt Study in ACE iNtolerant subjects with cardiovascular Disease subanalysis. J Hypertens. 2014;32(6):1334-41. PubMed PMID: 24621807.
72. Kohlmann O Jr, Roca-Cusachs A, Laurent S, Schmieder RE, Wenzel RR, Fogari R. Fixed-dose manidipine/delapril versus losartan/hydrochlorothiazide in hypertensive patients with type 2 diabetes and microalbuminuria. Adv Ther. 2009;26(3):313-24. PubMed PMID: 19330493.
73. Mehdi UF, Adams-Huet B, Raskin P, Vega GL, Toto RD. Addition of angiotensin receptor blockade or mineralocorticoid antagonism to maximal angiotensin-converting enzyme inhibition in diabetic nephropathy. J Am Soc Nephrol. 2009;20(12):2641-50. PubMed PMID: 19926893.
74. Mauer M, Zinman B, Gardiner R, Suissa S, Sinaiko A, Strand T, Drummond K, Donnelly S, Goodyer P, Gubler MC, Klein R. Renal and retinal effects of enalapril and losartan in type 1 diabetes. N Engl J Med. 2009;361(1):40-51. PubMed PMID: 19571282.
75. Nakao K, Hirata M, Oba K, Yasuno S, Ueshima K, Fujimoto A, Ogihara T, Saruta T; CASE-J Trial Group. Role of diabetes and obesity in outcomes of the candesartan antihypertensive survival evaluation in Japan (CASE-J) trial. Hypertens Res. 2010;33(6):600-6. PubMed PMID: 20379187.
76. Haller H, Ito S, Izzo JL Jr, Januszewicz A, Katayama S, Menne J, Mimran A, Rabelink TJ, Ritz E, Ruilope LM, Rump LC, Viberti G; ROADMAP Trial Investigators. Olmesartan for the delay or prevention of microalbuminuria in type 2 diabetes. N Engl J Med. 2011;364(10):907-17. PubMed PMID: 21388309.
77. Imai E, Chan JC, Ito S, Yamasaki T, Kobayashi F, Haneda M, Makino H; ORIENT study investigators. Effects of olmesartan on renal and cardiovascular outcomes in type 2 diabetes with overt nephropathy: a multicentre, randomised, placebo-controlled study. Diabetologia. 2011 Dec;54(12):2978-86. PubMed PMID: 21993710.
78. Imai E, Haneda M, Yamasaki T, Kobayashi F, Harada A, Ito S, Chan JC, Makino H. Effects of dual blockade of the renin-angiotensin system on renal and cardiovascular outcomes in type 2 diabetes with overt nephropathy and hypertension in the ORIENT: a post-hoc analysis (ORIENT-Hypertension). Hypertens Res. 2013;36(12):1051-9. PubMed PMID: 24026038.
79. Ruggenenti P, Lauria G, Iliev IP, Fassi A, Ilieva AP, Rota S, Chiurchiu C, Barlovic DP, Sghirlanzoni A, Lombardi R, Penza P, Cavaletti G, Piatti ML, Frigeni B, Filipponi M, Rubis N, Noris G, Motterlini N, Ene-Iordache B, Gaspari F, Perna A, Zaletel J, Bossi A, Dodesini AR, Trevisan R, Remuzzi G; DEMAND Study Investigators. Effects of manidipine and delapril in hypertensive patients with type 2 diabetes mellitus: the delapril and manidipine for nephroprotection in diabetes (DEMAND) randomized clinical trial. Hypertension. 2011;58(5):776-83. PubMed PMID: 21931073.
80. Parving HH, Brenner BM, McMurray JJ, de Zeeuw D, Haffner SM, Solomon SD, Chaturvedi N, Persson F, Desai AS, Nicolaides M, Richard A, Xiang Z, Brunel P, Pfeffer MA; ALTITUDE Investigators. Cardiorenal end points in a trial of aliskiren for type 2 diabetes. N Engl J Med. 2012;367(23):2204-13. PubMed PMID: 23121378.
81. Muramatsu T, Matsushita K, Yamashita K, Kondo T, Maeda K, Shintani S, Ichimiya S, Ohno M, Sone T, Ikeda N, Watarai M, Murohara T; NAGOYA HEART Study Investigators. Comparison between valsartan and amlodipine regarding cardiovascular morbidity and mortality in hypertensive patients with glucose intolerance: NAGOYA HEART Study. Hypertension. 2012;59(3):580-6. PubMed PMID: 22232134.
82. Yamashita K, Kondo T, Muramatsu T, Matsushita K, Nagahiro T, Maeda K, Shintani S, Murohara T. Effects of valsartan versus amlodipine in diabetic hypertensive patients with or without previous cardiovascular disease. Am J Cardiol. 2013;112(11):1750-6. PubMed PMID: 24035165.
83. Fried LF, Emanuele N, Zhang JH, Brophy M, Conner TA, Duckworth W, Leehey DJ, McCullough PA, O'Connor T, Palevsky PM, Reilly RF, Seliger SL, Warren SR, Watnick S, Peduzzi P, Guarino P; VA NEPHRON-D Investigators. Combined angiotensin inhibition for the treatment of diabetic nephropathy. N Engl J Med. 2013;369(20):1892-903. PubMed PMID: 24206457.
84. Maggioni AP, Greene SJ, Fonarow GC, Böhm M, Zannad F, Solomon SD, Lewis EF, Baschiera F, Hua TA, Gimpelewicz CR, Lesogor A, Gheorghiade M; ASTRONAUT Investigators and Coordinators. Effect of aliskiren on post-discharge outcomes among diabetic and non-diabetic patients hospitalized for heart failure: insights from the ASTRONAUT trial. Eur Heart J. 2013;34(40):3117-27. PubMed PMID: 23999456.
85. Gheorghiade M, Böhm M, Greene SJ, Fonarow GC, Lewis EF, Zannad F, Solomon SD, Baschiera F, Botha J, Hua TA, Gimpelewicz CR, Jaumont X, Lesogor A, Maggioni AP; ASTRONAUT Investigators and Coordinators. Effect of aliskiren on postdischarge mortality and heart failure readmissions among patients hospitalized for heart failure: the ASTRONAUT randomized trial. JAMA. 2013;309(11):1125-35. Erratum in: JAMA. 2013;309(14):1461. PubMed PMID: 23478743.
86. Ogihara T, Saruta T, Rakugi H, Saito I, Shimamoto K, Matsuoka H, Shimada K, Ito S, Horiuchi M, Imaizumi T, Takishita S, Higaki J, Katayama S, Kimura G, Umemura S, Ura N, Hayashi K, Odawara M, Tanahashi N, Ishimitsu T, Kashihara N, Morita S, Teramukai S; COLM Investigators. Combinations of olmesartan and a calcium channel blocker or a diuretic in elderly hypertensive patients: a randomized, controlled trial1. J Hypertens. 2014;32(10):2054-63. PubMed PMID: 24999799.
87. Matsui K, Kim-Mitsuyama S, Ogawa H, Jinnouchi T, Jinnouchi H, Arakawa K; OlmeSartan Calcium Antagonists Randomized (OSCAR) Study Group. Sex differences in response to angiotensin II receptor blocker-based therapy in elderly, high-risk, hypertensive Japanese patients: a subanalysis of the OSCAR study. Hypertens Res. 2014;37(6):526-32. PubMed PMID: 24599010.
88. Kim-Mitsuyama S, Ogawa H, Matsui K, Jinnouchi T, Jinnouchi H, Arakawa K. An angiotensin II receptor blocker-calcium channel blocker combination prevents cardiovascular events in elderly high-risk hypertensive patients with chronic kidney disease better than high-dose angiotensin II receptor blockade alone. Kidney Int. 2013;83(1):167-76. PubMed PMID: 23051740.
